# Giant coercivity and high magnetic blocking temperatures for N_2_^3−^ radical-bridged dilanthanide complexes upon ligand dissociation

**DOI:** 10.1038/s41467-017-01553-w

**Published:** 2017-12-15

**Authors:** Selvan Demir, Miguel I. Gonzalez, Lucy E. Darago, William J. Evans, Jeffrey R. Long

**Affiliations:** 1 Department of Chemistry, University of California, Berkeley, CA 94720 USA; 2 University of Goettingen, Institute of Inorganic Chemistry, Tammannstrasse 4, 37077 Goettingen, Germany; 3 Department of Chemistry, University of California, Irvine, CA 92697 USA; 4 Department of Chemical and Biomolecular Engineering, University of California, Berkeley, CA 94720 USA; 5 Materials Sciences Division, Lawrence Berkeley National Laboratory, Berkeley, CA 94720 USA

## Abstract

Increasing the operating temperatures of single-molecule magnets—molecules that can retain magnetic polarization in the absence of an applied field—has potential implications toward information storage and computing, and may also inform the development of new bulk magnets. Progress toward these goals relies upon the development of synthetic chemistry enabling enhancement of the thermal barrier to reversal of the magnetic moment, while suppressing alternative relaxation processes. Herein, we show that pairing the axial magnetic anisotropy enforced by tetramethylcyclopentadienyl (Cp^Me4H^) capping ligands with strong magnetic exchange coupling provided by an N_2_^3−^ radical bridging ligand results in a series of dilanthanide complexes exhibiting exceptionally large magnetic hysteresis loops that persist to high temperatures. Significantly, reducing the coordination number of the metal centers appears to increase axial magnetic anisotropy, giving rise to larger magnetic relaxation barriers and 100-s magnetic blocking temperatures of up to 20 K, as observed for the complex [K(crypt-222)][(Cp^Me4H^_2_Tb)_2_(μ−${\rm{N}}_2^ \cdot$)].

## Abstract (web summary)

Single-molecule magnets typically only retain information in the presence of an applied magnetic field and at very low temperatures. Here, Demir, Long and co-workers design N_2_^3–^ radical-bridged dilanthanide complexes that exhibit giant coercivities and 100-s magnetic blocking temperatures of up to 20 K.

## Introduction

Single-molecule magnets are molecules in which a strong axial anisotropy, imparted by the ligand field surrounding one or more metal centers, acts to create a bistable ground state, for which an activation barrier *U* must be overcome to reverse the orientation of the magnetic moment. The potential application of such species in high-density information storage^1^, as well as quantum computing^2^ and spin-based electronics^3^, hinges upon improving not just the defining thermal energy barrier to magnetization reversal, but also the magnetic blocking temperature and coercive field—metrics that determine the ability of the molecules to retain information upon removal of an applied magnetic field 4. The use of selected 4f metal ions, particularly Tb^III^, Dy^III^, and Er^III^, has recently dominated this area of research due to the advantageous combination of high magnetic moment and intrinsic magnetic anisotropy engendered by large spin–orbit coupling^4–11^. Progress in increasing blocking temperatures and coercive fields has been challenging mainly owing to the prevalence of through-barrier relaxation pathways, such as quantum tunneling of the magnetization, though recent reports on a molecule containing a single Dy^III^ ion demonstrate Orbach relaxation proceeding through nearly the entire effective magnetization reversal barrier (*U*_eff_) of greater than 1200 cm^−1^, with a correspondingly high 100-s blocking temperature^12, 13^.

Coordination chemistry can be utilized to yield electronic structures designed to prevent through-barrier relaxation pathways, with the goal of attaining magnetic blocking temperatures that scale with the magnitude of *U*_eff_^14^. One strategy for suppressing quantum tunneling of the magnetization is to establish rigorously symmetry-protected pure *M*_J_ states^15^, a condition as-yet only experimentally achieved in adatom-surface experiments^16^, though reports of the high-blocking temperature [Dy(Cp^ttt^)_2_]^+^ (Cp^ttt^ = 1,2,4-tri(*tert*-butyl)cyclopentadienide) complex suggest that rigorous symmetry may not be crucial to achieve high performance in single-ion magnetic molecules^12, 13^. Another promising route is the design of systems with strong intramolecular magnetic coupling between two or more metal centers. In addition to creating a more classical coupled system with large total angular momentum, magnetic coupling has been postulated to generate an exchange bias field that impedes quantum tunneling of the magnetization^17–20^. In recent years, a particularly successful strategy to promote strong magnetic coupling between lanthanide centers has been the employment of paramagnetic bridging ligands^21–28^. Significantly, the diffuse spin orbitals of anionic radical ligands are better able to penetrate into the core electron density of the deeply buried 4f orbitals. Our strategy toward high-performance single-molecule magnets therefore targets systems in which radical ligands facilitate a direct exchange coupling with lanthanide ions constrained in coordination environments that promote strong axial magnetic anisotropy. Here we show that this can be achieved in two new series of organometallic N_2_^3−^ radical-bridged dilanthanide complex salts: [K(crypt-222)(THF)][(Cp^Me4H^_2_Ln(THF))_2_(μ−${\rm{N}}_2^ \cdot$)] (crypt-222 = 2.2.2-cryptand, THF = tetrahydrofuran, Cp^Me4H^ = tetramethylcyclopentadienyl, Ln = Gd (**1-Gd**), Tb (**1-Tb**), Dy (**1-Dy**)) and [K(crypt-222)][(Cp^Me4H^_2_Ln)_2_(μ−${\rm{N}}_2^ \cdot$)], (Ln = Tb (**2-Tb**), Dy (**2-Dy**)). Notably, **2-Tb** exhibits the highest coercive field yet observed for any molecular magnet, substantially larger even than those of commercial permanent magnets, as well as the highest 100-s blocking temperature for a terbium single-molecule magnet.

## Results

### Synthesis and structural studies

**Fig. 1:**
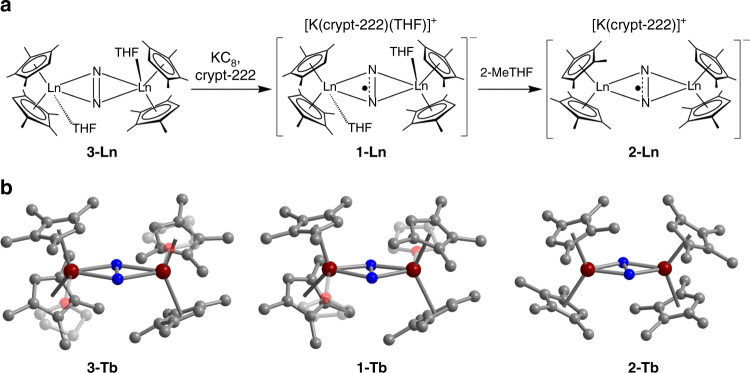
Synthesis and molecular structures of radical complexes. **a** Synthetic scheme for **1-Ln** and **2-Ln**. **b** Structure of the non-radical N_2_^2–^-bridged complex **3-Ln**, and structure of the N_2_^3−^ radical-bridged anions in crystals of **1-Ln** and **2-Ln**. Dark red, red, blue, and gray spheres represent Tb, O, N, and C atoms, respectively; H atoms have been omitted and THF groups faded for clarity. Selected interatomic distances (Å) and angles (deg) for **1-Gd**, **1-Tb**, and **1-Dy**, respectively: N–N = 1.362(9), 1.371(6), 1.374(3); mean Ln–N = 2.288(8), 2.257(4), 2.236(2); Ln···Ln = 4.344(1), 4.294(3), 4.244(1); Ln–N–N–Ln = 173.3(3), 173.4(3), 173.1(1). Selected interatomic distances (Å) for **2-Tb** and **2-Dy**, respectively: N–N = 1.392(9), 1.389(12); mean Ln–N = 2.221(6), 2.226(8); Ln···Ln = 4.216(1), 4.230(1); Ln–N–N–Ln = 178.3(3), 178.7(3)

The dilanthanide precursor complexes (Cp^Me4H^_2_Ln(THF))_2_(µ-N_2_) (Ln = Gd (**3-Gd**), Tb (**3-Tb**), Dy (**3-Dy**)) are readily obtained via treatment of Cp^Me4H^_2_Ln(BPh_4_) with KC_8_, which prompts the reduction of dinitrogen^29, 30^. Subsequent syntheses of compounds of type **1-Ln** employ KC_8_ in the presence of 2.2.2-cryptand in THF to reduce the N_2_^2−^-bridged molecules of **3-Ln** (Fig. 1). The first series of radical-bridged complex salts then comprises the isostructural compounds **1-Gd**, **1-Tb**, and **1-Dy**, which feature two crystallographically inequivalent lanthanide centers that are each ligated by two tetramethylcyclopentadienyl rings, one THF molecule, and a radical N_2_^3−^ bridging ligand that is coordinated in a side-on fashion (Fig. 1 and Supplementary Figs. 1 and 2). The THF ligands in compounds **1-Tb** and **1-Dy** can be readily dissociated by dissolution in 2-methyltetrahydrofuran (2-MeTHF), with the more sterically encumbered donor solvent enabling **2-Tb** and **2-Dy** to be crystallized from concentrated solutions. The isostructural compounds **2-Tb** and **2-Dy** are THF-free, resulting in decreased metal coordination numbers and increased Ln–N_2_^3−^–Ln dihedral angles of 178.5(2)° and 178.9(3)°, compared to 173.45(16)° and 173.14(8)° in **1-Tb** and **1-Dy**, respectively (Fig. 1 and Supplementary Fig. 3). The analogous reaction with **1-Gd** instead affords [K(crypt-222)][(Cp^Me4H^_2_Gd(2-MeTHF))_2_(μ−${\rm{N}}_2^ \cdot$)] (**4**), wherein the coordination of 2-MeTHF is a consequence of the larger radius of the Gd^III^ ion (Supplementary Fig. 4).

### Magnetic susceptibility measurements

**Fig. 2:**
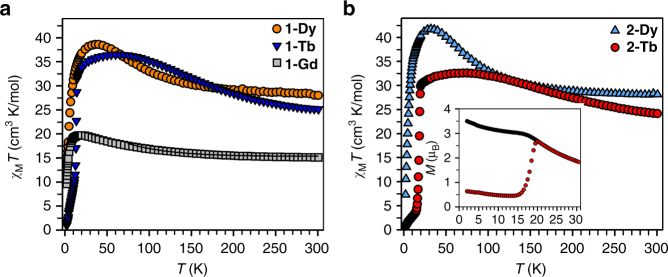
Temperature dependence of the *χ*_M_ *T* product for radical complexes. **a** Variable-temperature dc magnetic susceptibility data for restrained polycrystalline samples of **1-Dy** (orange circles), **1-Tb** (blue triangles), and **1-Gd** (gray squares) collected under a 1 T applied dc field. The black line represents a fit to the data for **1-Gd**, as discussed in the main text. **b** Variable-temperature dc magnetic susceptibility data for restrained polycrystalline samples of **2-Dy** (pale blue triangles) and **2-Tb** (red circles) collected under a 1 T applied dc field. Inset: plot of magnetization vs. temperature for **2-Tb** during field-cooled (black circles) and zero-field-cooled (red circles) measurements displaying the thermoremanent magnetization

Magnetic exchange coupling in the new compounds was first probed through measurement of the temperature dependence of the product of magnetic susceptibility and temperature (*χ*_M_ *T*). Complex **1-Gd** exhibits a room temperature *χ*_M_ *T* value of 15.3 emu·K/mol, somewhat lower than the value of 16.13 emu·K/mol expected for two magnetically isolated *S =* ^7^/_2_ Gd^III^ centers and an *S* = ^1^/_2_ radical spin. With decreasing temperature, a distinct rise in *χ*_M_ *T* is observed, up to a maximum value of 20.4 emu·K/mol at 10 K, owing to the presence of antiferromagnetic gadolinium-radical coupling which creates an *S* = ^13^/_2_ ground state that becomes isolated at low temperature. The magnetic coupling strength for **1-Gd** could be quantified using the spin-only Hamiltonian *Ĥ* = −2*J*_Gd–rad_ *Ŝ*_rad_·(*Ŝ*_Gd(1)_ *+ Ŝ*_Gd(2)_), which reveals a Gd–N_2_^3−^ coupling constant of *J*_Gd–rad_ = –20 cm^−1^ (Fig. 2 and Supplementary Figs. 5, 6 and 7). This represents the second largest coupling constant yet observed for a gadolinium-containing compound, following that of a similar N_2_^3−^-bridged complex with *J*_Gd–rad_ = –27 cm^−1^ ^21^, and is significantly greater than the *J*_Gd–rad_ of –11 cm^−1^ observed for both a mononuclear gadolinium–nitronyl nitroxide radical complex^31^ and an indigo radical-bridged digadolinium complex^28^. Such strong antiferromagnetic coupling reflects the diffuse character of the radical spin of the compact, highly anionic N_2_^3−^ bridging unit, which is localized in the π* orbital perpendicular to the Ln_2_(µ−η^2^:η^2^−N_2_) plane^32^.

Compounds **1-Dy**, **1-Tb**, **2-Dy**, and **2-Tb** exhibit similar increases in *χ*_M_ *T* with decreasing temperature, which are attributed to the presence of strongly coupled, higher-angular momentum ground states. Room temperature *χ*_M_ *T* values of 28.0 (**1-Dy**), 25.1 (**1-Tb)**, 28.1 (**2-Dy**), and 24.2 (**2-Tb**) emu·K/mol are observed under an applied field of 1 T. The *χ*_M_ *T* values expected for magnetically isolated Dy^III^_2_–N_2_^3−^ and Tb^III^_2_–N_2_^3−^ complexes are 28.7 and 24.0 emu·K/mol, respectively. The observed values then increase to local maxima of 38.6 emu·K/mol at 40 K (**1-Dy**), 36.5 emu·K/mol at 65 K (**1-Tb)**, 41.8 emu·K/mol at 32 K (**2-Dy**), and 32.6 emu·K/mol at 75 K (**2-Tb**) (Fig. 2 and Supplementary Figs. 5–25). Such dramatic increases in *χ*_M_ *T* again indicate strong coupling between the lanthanide centers and the N_2_^3−^ radical ligand, where the higher temperature of the maxima of **1-Tb** and **2-Tb** compared to those of **1-Dy** and **2-Dy** additionally suggest that the Ln–N_2_^3−^ coupling is stronger in the terbium congeners. While a subsequent decline in *χ*_M_ *T* with further decreasing temperature could be attributed to depopulation of Zeeman-split, coupled *M*_*J*_ states, the sudden, steep plummet observed for these complexes more likely signifies magnetic blocking due to a pinning of the moment along the easy axis within the immobilized crystallites at low temperatures. Indeed, magnetic blocking behavior is confirmed by the sharp divergence of the field-cooled and zero-field-cooled magnetic susceptibility data collected at 1 T, which occurs at 14.5 K for **1-Tb**, 7.5 K for **2-Dy**, and 20 K for **2-Tb** (Fig. 2 and Supplementary Figs. 26 and 27).

**Fig. 3:**
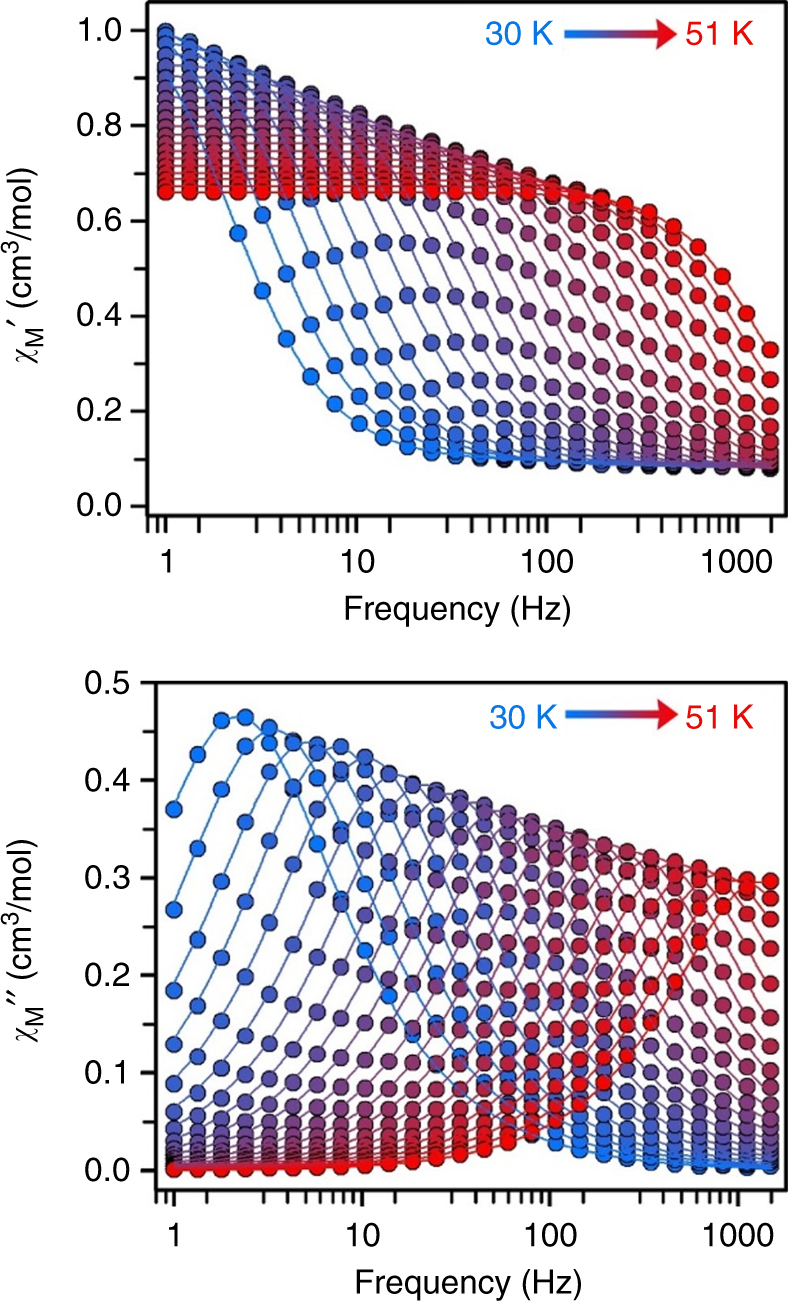
Dynamic magnetic susceptibility data. Variable-temperature, variable-frequency in-phase (*χ*_M_′) and out-of-phase (*χ*_M_″) ac magnetic susceptibility data collected for **2-Tb** under a zero-applied dc field from 30 to 51 K. A non-zero *χ*_M_″ out-of-phase signal suggests the presence of an energy barrier to spin reversal. Fits of a generalized Debye function to the *χ*_M_′ and *χ*_M_″ data afforded the relaxation times; solid lines represent fits to the data. Low and high-frequency ac magnetic susceptibility data are shown in the Supplementary Figs. 46 and 47

**Fig. 4:**
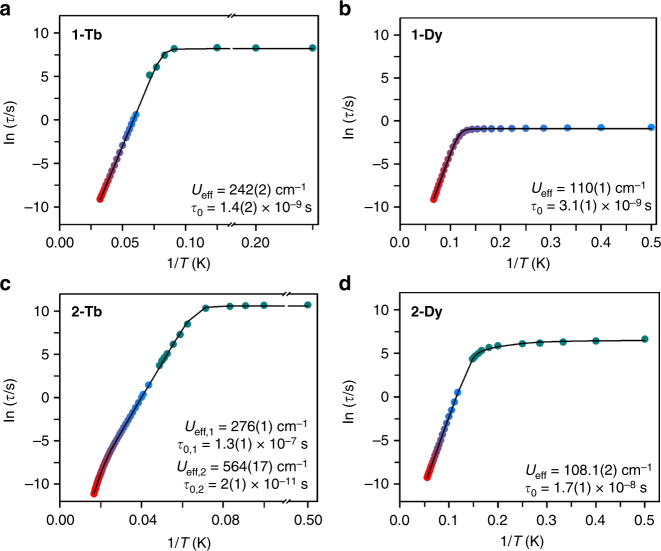
Arrhenius plots for radical complexes. Plots of the natural log of the relaxation time, *τ* (blue to red circles), vs. the inverse temperature for **a 1-Tb**, **b 1-Dy**, **c 2-Tb**, and **d 2-Dy**. Cyan circles represent data extracted from dc susceptibility measurements. Standard deviations of the relaxation times were determined from a nonlinear least-squares analysis employing the program SolverAid (Version 7) by R. de Levie (Microsoft Excel Macro, 2007); error bars are omitted as they are within the radius of the symbols. The black line corresponds to a fit of the data in the temperature range of 4–31 K for **1-Tb**, 2–15 K for **1-Dy**, 2–60 K for **2-Tb**, and 2–18 K for **2-Dy** to multiple relaxation processes (see Supplementary Methods and Supplementary Figs. 32, 39, 45, and 56)

Variable-frequency, variable-temperature in-phase (*χ*_M_′) and out-of-phase (*χ*_M_″) ac magnetic susceptibility data were collected in order to probe whether the magnetic blocking arises from single-molecule magnet behavior (Fig. 3 and Supplementary Figs. 28–49). Peaks were indeed observed in *χ*_M_″ for **1-Dy**, **1-Tb**, **2-Dy**, and **2-Tb**, indicative of slow magnetic relaxation. Magnetic relaxation times, *τ*, were extracted from fitting plots of *χ*_M_″ vs. *χ*_M_′ using a generalized Debye equation, and were then used to construct Arrhenius plots (Fig. 4 and Supplementary Fig. 49). These plots enable analysis of the temperature dependence of the magnetic relaxation times, which yields detailed insights into what magnetic relaxation pathways are operational. In particular, a thermally activated Orbach process shows an exponential dependence of *τ* upon temperature, while a quantum tunneling process is temperature independent. The various types of relaxation pathways observed reveal key correlations between structure and magnetic relaxation behavior in lanthanide–radical molecular magnets. Moreover, the magnetic blocking temperature, *T*_b_, for a single-molecule magnet is best evaluated using such relaxation time data, which, unlike hysteresis or field-cooled/zero-field-cooled measurements, are not sweep rate dependent. In particular, the temperature associated with a magnetic relaxation time of 100 s has been suggested as the standard for comparison of single-molecule magnets^33^.

**Fig. 5:**
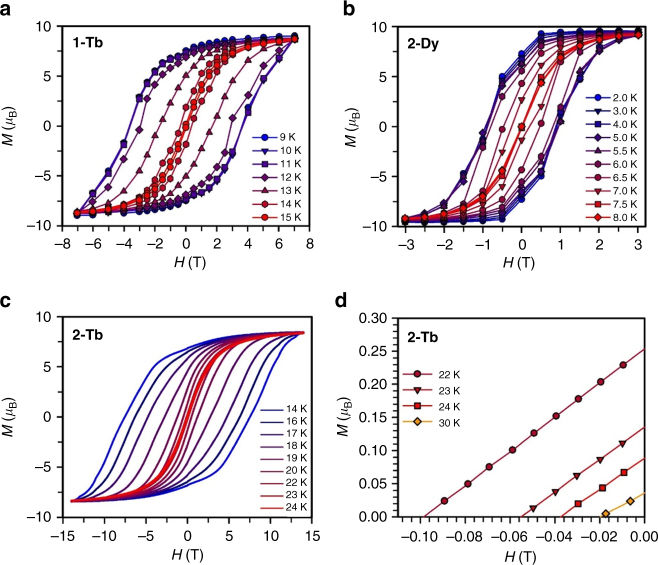
Magnetic hysteresis data for radical complexes. Plot of magnetization (*M*) vs. dc magnetic field (*H*) at an average sweep rate of 0.01 T/s for **a 1-Tb** from 9 to 15 K, **b 2-Dy** from 2 to 8 K, **c 2-Tb** from 14 to 24 K, **d 2-Tb** from 22 to 30 K. The magnetic hysteresis loops are effectively closed at 15 K for **1-Tb**, 7.5 K for **2-Dy**, and 30 K for **2-Tb**, and are open for lower temperatures. For **2-Tb**, the coercive fields observed at 22, 23, and 24 K are ~0.1, 0.06, and 0.04 T, respectively

The relaxation time data for **1-Dy** were fit using two relaxation processes, an Orbach process, and a quantum tunneling process to yield a thermal relaxation barrier of *U*_eff_ = 110(1) cm^−1^, together with a relaxation attempt time of *τ*_0_ = 3.1(1) × 10^−9^ s (Fig. 4b, Supplementary Figs. 28–32, and Supplementary Table 1). The presence of quantum tunneling typically precludes magnetic blocking. Consequently, **1-Dy** exhibits substantially waist-restricted hysteresis loops even at 2 K, at which temperature the complex achieves a maximum magnetic relaxation time of just 0.41 s (Supplementary Fig. 33). In contrast, the thermally activated relaxation regime, in which the relaxation time increases exponentially as the temperature is lowered, persists over the entire range of temperatures accessible in the ac susceptibility measurements for **1-Tb** and **2-Dy**, yielding effective spin-reversal barriers, *U*_eff,_ of 242(2) and 108.1(2) cm^−1^, respectively (Supplementary Figs. 34–45, and Supplementary Tables 2 and 3). Variable-field magnetization measurements on the latter two complexes reveal wide magnetic hysteresis loops with no evidence of tunneling, and 100-s magnetic blocking temperatures of 14 and 6.6 K, respectively, were determined from dc relaxation measurements (Figs. 4 and 5 and Supplementary Figs. 38 and 44).

The prevalence of quantum tunneling for **1-Dy** likely can be ascribed to a reduction in the axiality of the Dy^III^ ground states due to THF coordination. Indeed, this explanation is supported by the enhanced relaxation times and wide magnetic hysteresis exhibited by **2-Dy** below 7.5 K (Fig. 5b), in conjunction with the removal of coordinated THF and enhanced axial symmetry.

In the case of **1-Tb**, the substantially stronger exchange coupling with the radical N_2_^3−^ ligand, first noted in the relatively high-temperature maximum in *χ*_M_ *T*, coincides with relaxation times as long as 1.11 h and wide magnetic hysteresis, in spite of the presence of coordinated THF. This result suggests that an enhancement of axial anisotropy of the Tb^III^ ions in **2-Tb** via loss of coordinated THF should further improve upon this outstanding slow magnetic relaxation behavior, in analogy to the improvement in the magnetic behavior of **2-Dy** relative to **1-Dy**.

Magnetic relaxation time data for **2-Tb** were collected over an extended temperature range of 2–60 K using dc relaxation and high-frequency ac susceptibility techniques (Fig. 4c, Supplementary Figs. 46–56, and Supplementary Table 4). The data reveal relaxation times as long as 12.6 h at low temperatures, and, significantly, a deviation from linearity at the highest temperatures probed. Consequently, fitting the data required implementation of not just one, but two Orbach processes in addition to a quantum tunneling term, yielding relaxation barriers of *U*_eff,1_ = 276(1) cm^-^^1^ and *U*_eff,2_ = 564(17) cm^−1^. The first barrier of 276 cm^−1^ represents to the best of our knowledge the highest value yet observed for a radical-bridged single-molecule magnet, followed by barriers of *U*_eff_ = 242 cm^−1^ for **1-Tb** and *U*_eff_ = 227 cm^−1^ for [(((Me_3_Si)_2_N)_2_Tb(THF))_2_(μ−${\rm{N}}_2^ \cdot$)]^−^^22^. Consistent with this activation barrier, the 100-s blocking temperature for **2-Tb** is measured to be 20 K, setting a record for both terbium-based and multinuclear single-molecule magnets. Dramatic improvements in magnetic behavior for **2-Tb** and **2-Dy** compared to **1-Tb** and **1-Dy** appear to reflect a reduction in transverse anisotropy of the Ln^III^ ions, although an additional factor may be the increase in planarity of the Ln_2_N_2_ unit upon removal of THF. This reduction in transverse anisotropy appears to facilitate an unperturbed, strong antiferromagnetic lanthanide–radical coupling for **2-Tb** and **2-Dy**, while also likely suppressing unfavorable antiferromagnetic lanthanide–lanthanide interactions^34^. Together, a reduction of both transverse anisotropy and detrimental exchange coupling pathways should increase not only the axiality of the ground state, but also that of exchange-coupled excited states, possibly enabling observation of magnetic relaxation through excited states^20, 35^.

With this in mind, one point of note regarding the two thermal activation barriers to reversal of magnetization observed for **2-Tb** is that the *U*_eff,2_ barrier of 564 cm^−1^ is close to double that of the *U*_eff,1_ barrier of 276 cm^−1^. These results may thus constitute the first evidence of magnetic relaxation through a “spin ladder” for a lanthanide–radical single-molecule magnet, in which the energy of each rung is determined by a multiple of 2*J*_Ln–rad_ (Supplementary Fig. 57). Computational studies of N_2_^3−^-bridged complexes have suggested that if crystal field splitting of the Ln^III^ ions is sufficiently high compared to the magnitude of exchange coupling, mixing of exchange- and crystal field-split doublets is reduced, promoting axiality of exchange doublets^36^. Under this condition, magnetic relaxation may proceed through multiple exchange doublets, rather than being limited by the energy of the first excited state, as in the case of an excited doublet with mixed exchange and crystal field character.

### Modeling dc magnetic susceptibility data

A model of the electronic structure of these complexes using the following Hamiltonian reveals a more explicit correlation between lanthanide–radical coupling strength and the thermal barriers to magnetic relaxation^37^.

1 $$\hat H = - 2{J_{{\rm{Ln}} - {\rm{rad}}}}{\hat S_{{\rm{rad}}}} \cdot \left( {{{\hat J}_{{\rm{Ln}}\left( 1 \right)}} + {{\hat J}_{{\rm{Ln}}\left( 2 \right)}}} \right) + \mathop {\sum }\limits_{i = {\rm{Ln}}\left( 1 \right),{\rm{Ln}}\left( 2 \right)} B_2^0O_2^0\left( i \right)$$

This model assumes a large axial anisotropy, represented by the $B_2^0$ parameter, as well as an isotropic exchange interaction between the angular momenta of the Ln^III^ centers and the N_2_^3−^ radical spin^38, 39^. The *χ*_M_ *T* vs. *T* data may be reasonably modeled using lanthanide–radical exchange coupling values of *J*_Ln–rad_ = –7.2 and –23.1 cm^−1^ for **2-Dy** and **2-Tb**, respectively, along with slight modulation of *g* values around the expected *g*_*J*_ values for Dy^III^ and Tb^III^ ions (further details are provided in Supplementary Methods, Supplementary Figs. 58 and 59, and Supplementary Tables 5 and 6). Excited state energies defined by Ising exchange coupling correspond to multiples of *J*_Ln–rad_ based on the magnitude of Δ*J* resulting from a spin flip. For instance, in **2-Tb**, the first excited state, corresponding to a flip of a single Tb^III^ spin, has an energy of 12*J*_Tb–rad_, while the second state, corresponding to a flip of the N_2_^3-^ radical spin, has an energy of 24*J*_Tb–rad_. Using this formalism, *U*_eff_ estimates of 108 and 277 cm^−1^ can be obtained for **2-Dy** and **2-Tb**, respectively, in excellent agreement with the experimentally observed values of 108 and 276 cm^−1^. This model also estimates a second excited state energy double that of the first, or 554 cm^−1^ for **2-Tb**, which is in reasonable agreement with the experimentally observed value of *U*_eff,2_ = 564 cm^−1^.

The excited states defined by exchange coupling within these molecules will certainly mix with crystal field-split *M*_J_ states to generate a perturbed excited state spectrum, and a more accurate assessment of the electronic structure would therefore entail utilizing multiple exchange parameters^36, 40^. However, this simple model seems to have surprising utility for predicting excited state energies, and thereby magnetic relaxation barriers for lanthanide–radical molecules. In particular, it provides insight into the much lower *U*_eff_ values observed for **1-Dy** and **2-Dy** compared to **1-Tb** and **2-Tb**, which can be attributed to the substantially weaker exchange coupling in the dysprosium complexes. This model further provides a rationale for the very similar relaxation barriers for each pair of compounds, **1-Ln** and **2-Ln**: upon dissociation of THF, the geometry of the Ln_2_(μ−${\rm{N}}_2^ \cdot$) core unit is scarcely perturbed, leading to similar exchange constants and comparable *U*_eff_ values.

### Analysis of magnetic hysteresis and coercivity

Finally, single-molecule magnet performance in storage applications mandates retention of a non-zero magnetization under zero-applied field. Variable-field magnetization measurements were therefore performed using an average sweep rate of 0.01 T/s (Fig. 5). While **1-Dy** does not display a remanent magnetization at any temperature, hysteresis loops for **2-Dy** remain open up to 8 K, with a maximum *H*_c_ of 1 T at 5.5 K. The stark difference in hysteresis behavior between **1-Dy** and **2-Dy** again emphasizes the importance of THF removal from the metal centers as a means of enhancing axiality. Complex **1-Tb** exhibits open hysteresis curves up to 15 K, with a maximum *H*_c_ of 3.7 T obtained at 11 K. In contrast, open magnetic hysteresis loops were observed for **2-Tb** at temperatures as high as 30 K, and with a substantial *H*_c_ value of 0.48 T at 20 K. The coercive field steadily increases as the temperature is lowered to a giant coercive field of 7.9 T at 10 K, which then remains constant down to 2 K (Supplementary Figs. 60–61). Significantly, this is the largest coercive field yet observed for any molecule or coordination solid, and indeed is substantially larger than those of commercial permanent magnets, including Nd_14_Fe_80_B_6_ (_i_ *H*_c = _1.39 T at 298 K and 3.90 T at 77 K) and SmCo_5_ (_i_ *H*_c = _2.9 T at 298 K and _i_ *H*_c_ = 4.3 T at 4.2 K)^41–45^.

## Discussion

Modulation of the ligand field of the lanthanide ions in N_2_^3‒^-radical-bridged dilanthanide complexes has produced valuable insights into the magnetic relaxation behavior for this important set of single-molecule magnets. As isolated in **1-Gd**, **1-Tb**, and **1-Dy**, complexes of the type [(Cp^Me4H^_2_Ln(THF))_2_(μ−${\rm{N}}_2^ \cdot$)]^−^ exhibit magnetic properties that are remarkably similar to those of the previously reported [(((Me_3_Si)_2_N)_2_Ln(THF))_2_(μ−${\rm{N}}_2^ \cdot$)]^−^ complexes, with replacement of the (Me_3_Si)_2_N^−^ ligands with Cp^Me4H−^ ligands leading to comparable exchange coupling constants, relaxation barriers, blocking temperatures, and coercive fields (Table 1). Importantly, however, upon removal of the THF ligands to form [(Cp^Me4H^_2_Ln)_2_(μ−${\rm{N}}_2^ \cdot$)]^–^ species, as isolated in **2-Tb** and **2-Dy**, a marked increase in magnetic anisotropy arises, leading to substantial increases in blocking temperatures and coercive fields. Indeed, **2-Tb** displays a relaxation barrier of *U*_eff_ = 276 cm^−1^ and a 100-s blocking temperature of *T*_b_ = 20 K, both records for exchange-coupled single-molecule magnets, as well as a coercive field of *H*_c_ = 7.9 T at 10 K, the largest yet observed for any coordination compound. Furthermore, a simple model of the electronic structure for such radical-bridged dilanthanide complexes clearly relates the relaxation barrier to the exchange coupling constant, for example, through the equations *U*_eff_ = 12*J*_Tb-rad_ for diterbium(III) complexes and *U*_eff_ = 15*J*_Dy-rad_ for didysprosium(III) complexes. Thus, a path toward improved single-molecule magnets of this type is clear: replacing N_2_^3‒^ with a radical bridge that provides stronger exchange coupling can be expected to increase *U*_eff_ substantially, while further adjustments to the ligand field arising from the capping ligands can enhance axiality, ensuring that the observed blocking temperature is not diminished by through-barrier relaxation processes and potentially yielding even greater coercive fields.

**Table 1:** Key magnetic properties of N_2_^3−^ radical-bridged lanthanide single-molecule magnets

	*J*_est_(cm^-1^)	*U*_eff_(cm^-1^)	*τ*_0_ (s)	*T*_b_ (K)^a^	*H*_c_ (T)	References
[(((Me_3_Si)_2_N)_2_Tb(THF))_2_(μ−${\rm{N}}_2^ \cdot$)]^−^		227	8.2 × 10^−9^	14	4.7 T (11 K)^b^	22
[(Cp^Me4H^_2_Tb(THF))_2_(µ−${\rm{N}}_2^ \cdot$)]^−^ (**1-Tb**)	−20.2	242	1.4 × 10^−9^	14	3.7 T (11 K)^b^	This work
[(Cp^Me4H^_2_Tb)_2_(µ−${\rm{N}}_2^ \cdot$)]^−^ (**2-Tb**)	−23.1	276	1.3 × 10^−7^	20	7.9 T (10 K)^c^	This work
[(((Me_3_Si)_2_N)_2_Dy(THF))_2_(μ−${\rm{N}}_2^ \cdot$)]^−^		123	8.0 × 10^−9^	8.3	1.2 T (8 K)^d^	21
[(Cp^Me4H^_2_Dy(THF))_2_(µ−${\rm{N}}_2^ \cdot$)]^−^ (**1-Dy**)	−7.3	110	3.1 × 10^−9^			This work
[(Cp^Me4H^_2_Dy)_2_(µ−${\rm{N}}_2^ \cdot$)]^−^ (**2-Dy**)	−7.2	108	1.7 × 10^−8^	6.6	1 T (5.5 K)^d^	This work

^a^Blocking temperature defined as the temperature at which the magnetic relaxation time is 100 s

^b^Sweep rate = 0.009 T/s

^c^Sweep rate = 0.01 T/s

^d^Sweep rate = 0.008 T/s

## Methods

### General synthesis

The manipulations described below were performed under an inert atmosphere with rigorous exclusion of air and water using Schlenk, vacuum line, and glovebox techniques. Solvents were dried using a commercial solvent purification system from JC Meyer Solvent Systems (http://www.jcmeyer-solventsystems.com). The precursor 1,2,3,4-tetramethylcyclopentadiene (Cp^Me4H^H) was purchased from Sigma-Aldrich and dried over 4 Å sieves before use. Anhydrous LnCl_3_ (Ln = Gd, Tb, Dy) was purchased from Sigma-Aldrich and used as received. Potassium bis(trimethylsilyl)amide, KN[Si(CH_3_)_3_]_2_, was purchased from Sigma-Aldrich, dissolved in toluene, filtered through Celite, and recrystallized from toluene at −35 °C before use. The compound 4,7,13,16,21,24-hexaoxa-1,10-diazabicyclo[8.8.8]-hexacosane (2.2.2-cryptand; here abbreviated as crypt-222) was purchased from TCI America and used as received. The compounds, KCp^Me4H^ ^29^ and KC_8_^46^, were prepared according to literature procedures. Synthesis details for the precursor complexes Cp^Me4H^_2_Ln(η^3^-C_3_H_5_) and Cp^Me4H^_2_Ln(BPh_4_), (Supplementary Figs. 62–67), where Ln = Gd, Tb, Dy, are described in the Supplementary Methods, and their preparation followed closely the route used for Cp^Me4H^_2_Lu(BPh_4_) or Cp^Me4H^_2_Sc(BPh_4_)^29, 30^. Preparation of the complexes Cp^Me4H^_2_Ln(µ-Cl_2_)K(THF)_*x*_ and (Cp^Me4H^_2_Ln(THF))(µ-N_2_), **3-Ln**, (Supplementary Figs. 68–80), generally followed routes previously described in the literature for Lu^III^ and Sc^III^ analogs^29, 30^, while syntheses and modifications made in the case of **3-Ln** are described below. The structures of (Cp^Me4H^_2_Ln(THF))_2_(µ−N_2_), where Ln = Gd, Dy were published previously, although (Cp^Me4H^_2_Dy(THF))_2_(µ−N_2_) was reported in a different cell, and these compounds were synthesized by reduction of (C_5_Me_4_H)_3_Ln(THF)^47^. Elemental analyses were performed by the Micro-Mass Facility at the University of California, Berkeley, using a Perkin-Elmer Series 2400 Series II combustion analyzer. IR spectra were recorded on a Perkin-Elmer Avatar Spectrum 400 FTIR Spectrometer equipped with ATR.

### General procedure for the synthesis of (Cp^Me4H^_2_Ln(THF))_2_(µ−N_2_^.^), **3-Ln**, Ln = Gd, Tb, Dy

In a nitrogen-filled glovebox, Cp^Me4H^_2_Ln(BPh_4_) was dissolved in 15 ml of THF to give a colorless solution. Subsequently, potassium graphite was added at once whereby the solution color turned to orange–red. After 5 min of stirring at room temperature, black and white insoluble materials, presumably graphite and KBPh_4_, were removed by filtration. The orange–red filtrate was pumped down under reduced pressure to yield a solid that was extracted with 5 ml of toluene to afford a dark red solution. Green crystals of (Cp^Me4H^_2_Ln(THF))_2_(µ−N_2_) suitable for X-ray analysis were grown in the freezer over the course of 48 h.

***3-Gd***: Isolated 263 mg in crystalline yield (49%) from the reaction of 790 mg (1.10 mmol) of crystalline Cp^Me4H^_2_Gd(BPh_4_) and 149 mg (1.10 mmol) of potassium graphite. IR (neat): 2962 m, 2905 s, 2855 s, 2716 w, 1443 m, 1377 w, 1327 w, 1242 w, 1173 w, 1108 w, 1030 s, 917 w, 890 m, 875 s, 833 w, 777 s, 762 s, 741 m, 613 s cm^−1^Anal. Calcd for C_44_H_68_N_2_O_2_Gd_2_: C, 54.40; H, 7.06; N, 2.88. Found: C, 54.76; H, 7.21; N, 2.77.

***3-Tb***: Isolated 162.9 mg in crystalline yield (52%) from the reaction of 460.4 mg (0.639 mmol) of crystalline Cp^Me4H^_2_Tb(BPh_4_) and 86.4 mg (0.639 mmol) of potassium graphite. IR (neat): 2960 m, 2930 m, 2905 s, 2895 s, 2858 s, 1459 m, 1446 m, 1437 m, 1429 m, 1376 m, 1368 m, 1030 s, 917 m, 874 s, 860 s, 780 s, 765 s, 743 s, 663 m, 655 m, 615 s, 600 s cm^−1^. Anal. Calcd for C_44_H_68_N_2_O_2_Tb_2_: C, 54.21; H, 7.03; N, 2.87. Found: C, 54.31; H, 7.03; N, 2.79.

***3-Dy***: Isolated 131.2 mg in crystalline yield (55%) from the reaction of 350.3 mg (0.4837 mmol) of crystalline Cp^Me4H^_2_Tb(BPh_4_) and 65.4 mg (0.484 mmol) of potassium graphite. IR (neat): 2954 m, 2936 m, 2901 s, 2857 s, 2858 s, 1461 m, 1448 m, 1441 m, 1432 m, 1426 m, 1379 m, 1370 m, 1202 w, 1030 s, 916 w, 874 s, 862 s, 781 s, 763 s, 742 m, 665 m, 626 m, 616 s, 611 s cm^−1^. Anal. Calcd for C_44_H_68_N_2_O_2_Dy_2_: C, 53.81; H, 6.98; N, 2.85. Found: C, 53.84; H, 7.10; N, 2.82.

### General procedure for the synthesis of [K(crypt-222)(THF)][(Cp^Me4H^_2_Ln(THF))_2_(µ−N_2_^.^)], **1-Ln**, Ln = Gd, Tb, Dy

In an argon-filled glovebox, a 10 ml THF solution containing crypt-222 (6 ml in the case of Dy) was added to a 10 ml pale green THF solution of crystalline (Cp^Me4H^_2_Ln(THF))_2_(µ−N_2_) (6 ml in the case of Dy). Subsequently, potassium graphite was added at once to the reaction mixture, whereby the solution color turned to red–brown. After 5 min of stirring at room temperature, black insoluble material, presumably graphite, was removed by filtration. The dark brown filtrate was stored in the freezer for 24 h to afford dark brown crystals of **1-Ln** that were suitable for X-ray analysis.

***1-Gd***: Isolated 128 mg in crystalline yield (75%) from the reaction of 113.9 mg (0.1166 mmol) of crystalline (Cp^Me4H^_2_Gd(THF))_2_(µ−N_2_), 43.9 mg (0.117 mmol) of crypt-222, and 15.8 mg (0.117 mmol) potassium graphite. IR (neat): 2939 m, 2881 s, 2844 s, 2711 w, 1473 w, 1458 w, 1444 w, 1353 s, 1329 w, 1314 w, 1297 w, 1283 w, 1261 w, 1244 w, 1172 w, 1132 m, 1102 s, 1076 m, 1062 s, 1042 m, 1029 m, 946 s, 922 m, 901 m, 862 w, 826 w, 809 w, 788 w, 763 m, 746 m, 727 m, 727 w, 716 w, 702 w, 663 w, 616 w, 600 w cm^−1^. Anal. Calcd for C_66_H_112_KN_4_O_9_Gd_2_: C, 54.32; H, 7.74; N, 3.84. Found: C, 54.50; H, 7.89; N, 3.78.

***1-Tb***: Isolated 168 mg in crystalline yield (78%) from the reaction of 144 mg (0.148 mmol) of crystalline (Cp^Me4H^_2_Tb(THF))_2_(µ−N_2_), 55.7 mg (0.148 mmol) of crypt-222, and 20.0 mg (0.148 mmol) of potassium graphite. IR (neat): 2940 mbr, 2879 s, 2846 s, 2712 w, 1474 w, 1455 m, 1443 m, 1352 s, 1297 w, 1283 w, 1260 w, 1243 w, 1171 w, 1132 s, 1102 s, 1077 s, 1062 s, 1044 s, 1030 s, 947 s, 931 m, 922 m, 903 m, 856 w, 831 w, 824 w, 765 m, 746 s, 727 w, 615 w, 557 s cm^−1^. Anal. Calcd for C_66_H_112_KN_4_O_9_Tb_2_: C, 54.20; H, 7.72; N, 3.83. Found: C, 54.13; H, 7.85; N, 3.80.

***1-Dy***: Isolated 63.8 mg in crystalline yield (57%) from the reaction of 74.5 mg (0.0759 mmol) of crystalline (Cp^Me4H^_2_Dy(THF))_2_(µ−N_2_), 28.6 mg (0.0759 mmol) of crypt-222, and 10.3 mg (0.0759 mmol) of potassium graphite. IR (neat): 2938 mbr, 2878 s, 2847 s, 1476 m, 1459 m, 1452 m, 1443 m, 1353 s, 1298 w, 1284 w, 1259 w, 1242 w, 1172 w, 1133 s, 1101 vs, 1062 s, 1043 s, 947 s, 931 m, 922 m, 903 m, 883 w, 853 w, 822 w, 767 m, 746 s, 696 w, 672 w, 641 w, 614 w, 560 s cm^−1^. Anal. Calcd for C_66_H_112_KN_4_O_9_Dy_2_: C, 53.94; H, 7.68; N, 3.81. Found: C, 53.85; H, 7.88; N, 4.00.

### Procedure for the synthesis of [K(crypt-222)][(Cp^Me4H^_2_Ln)_2_(µ−N_2_^.^)], **2-Ln**, Ln = Tb, Dy

***2-Tb***: In an argon-filled glovebox, [K(crypt-222)(THF)][(Cp^Me4H^_2_Tb(THF))_2_(µ−N_2_)] (92.0 mg, 0.063 mmol) was dissolved in 12 ml of 2-MeTHF to give a brown–black solution. The solution was filtered and the brown–black filtrate was stored in the freezer for 48 h to afford brown–black crystals of **2-Tb** that were suitable for X-ray analysis (76 mg, 91%). Anal. Calcd for C_59_H_98_KN_4_O_7_Tb_2_: C, 53.19; H, 7.41; N, 4.21. Found: C, 53.34; H, 7.46; N, 4.12.

***2-Dy***: The same procedure was used as above, with 62.0 mg (0.042 mmol) [K(crypt-222)(THF)][(Cp^Me4H^_2_Dy(THF))_2_(µ−N_2_)], and 10 ml of 2-MeTHF to give a brown–black solution, which was filtered and stored in the freezer for 48 h to afford brown–black crystals of **2-Dy** that were suitable for X-ray analysis (51.0 mg, 90%). IR (neat): 2956 m, 2878 s, 2852 s, 2717 w, 1477 m, 1456 m, 1442 m, 1378 w, 1354 s, 1296 m, 1259 m, 1132 s, 1101 vs, 1079 s, 1022 m, 947 s, 932 s, 828 m, 819 m, 803 w, 765 s, 749 s, 620 m, 567 vs cm^−1^. Anal. Calcd for C_59_H_98_KN_4_O_7_Dy_2_: C, 52.90; H, 7.37; N, 4.18. Found: C, 53.06; H, 7.23; N, 4.28.

### Synthesis of [K(crypt-222)][(Cp^Me4H^_2_Gd(2-MeTHF)_2_(µ−N_2_^.^)], 4

In an argon-filled glovebox, [K(crypt-222)(THF)][(Cp^Me4H^_2_Gd(THF))_2_(µ−N_2_^.^)] (96.0 mg, 0.066 mmol) was dissolved in 10 ml of 2-MeTHF to give a brown–black solution. The solution was filtered and the brown–black filtrate was stored in the freezer for 48 h to afford brown–black crystals of **4** that were suitable for X-ray analysis (86.0 mg, 92%). IR (neat): 2952 m, 2879 s, 2842 s, 2711 w, 1473 m, 1441 m, 1376 w, 1350 m, 1327 w, 1297 m, 1256 m, 1232 w, 1172 w, 1131 s, 1114 s, 1101 vs, 1076 s, 1057 s, 1031 m, 1015 m, 991 w, 966 w, 951 s, 932 s, 896 w, 869 w, 859 w, 833 w, 820 m, 798 w, 751 s, 700 w, 684 w, 654 w, 615 w cm^−1^. Anal. Calcd for C_64_H_108_KN_4_O_8_Gd_2_: C, 54.32; H, 7.69; N, 3.96. Found: C, 54.10; H, 7.80; N, 3.82.

### Structural characterization of compounds

X-ray diffraction experiments were performed at 100 K on crystals coated with paratone-N oil and mounted on Kapton or MiTeGen loops. Data were collected at the small molecule X-ray crystallography facility at the University of California, Berkeley using a Bruker QUAZAR diffractometer equipped with a microfocus sealed X-ray source (Mo-Kα radiation; *λ* = 0.71073 Å) and a Bruker APEX-II detector for **2-Tb**, **4**, **3-Dy**, Cp^Me4H^_2_Dy(BPh_4_), Cp^Me4H^_2_Gd(BPh_4_), Cp^Me4H^_2_Tb(allyl), and Cp^Me4H^_2_Dy(allyl), or at Beamline 11.3.1 at the Advanced Light Source on a Bruker D8 Diffractometer equipped with a Bruker PHOTON100 CMOS detector using synchrotron radiation with *λ* = 0.6888 Å for **1-Dy** and Cp^Me4H^_2_Gd(allyl) and *λ* = 0.7749 Å for **1-Gd**, **1-Tb**, **2-Dy**, **3-Tb**, **3-Gd**, and Cp^Me4H^_2_Tb(BPh_4_) (further details are provided in Supplementary Methods and Supplementary Tables 7–9). Crystallographic data have been deposited in the Cambridge Structural Database as CCDC 1547761 (**2-Tb**),1547762 (**2-Dy**),1547763 (**4**), 1547764 (**1-Tb**) 1547765 (**1-Dy**), 1547766 (**1-Gd**), 1547767 (**3-Tb**), 1547768 (**3-Dy**), 1547769 (**3-Gd**), 1547770 (Cp^Me4H^_2_Tb(BPh_4_)), 1547771 (Cp^Me4H^_2_Dy(BPh_4_)), 1547772 (Cp^Me4H^_2_Gd(BPh_4_)), 1547773 (Cp^Me4H^_2_Tb(allyl)), 1547774 (Cp^Me4H^_2_Dy(allyl)), 1547775 (Cp^Me4H^_2_Gd(allyl)).

### Magnetic susceptibility measurements

The magnetic samples of **1-Ln** (Ln = Gd, Tb, Dy), **2-Ln** (Ln = Tb, Dy), and **3-Ln** (Ln = Gd, Dy, Tb) were prepared by adding crushed crystalline samples to 7 mm quartz tubes. Sufficient liquid eicosane (at 40 °C) was added to saturate and cover the samples to prevent crystallite torqueing and provide good thermal contact between the sample and the bath. Tubes were fitted with sealable adapters, evacuated on a Schlenk line, and flame sealed under vacuum using a H_2_/O_2_ flame. Magnetic susceptibility measurements were collected using a Quantum Design MPMSXL SQUID magnetometer. For the hysteresis loops of **2**-**Tb**, magnetic susceptibility measurements were collected using a Quantum Design 14 Tesla Dynacool magnetometer at the facility of Quantum Design in San Diego, CA. High-frequency ac measurements were also performed at the facility of Quantum Design in San Diego, CA, where a 9 T PPMS equipped with the ACMSII measurement option to probe the ac relaxation at frequencies above 1500 Hz was used. Dc susceptibility data measurements were performed at temperatures ranging from 2 to 300 K for **1-Ln** (Ln = Gd, Tb, Dy), **2-Ln** (Ln = Tb, Dy), and **3-Ln** (Ln = Gd, Dy, Tb) using applied fields of 1000, 5000, and 10,000 Oe. Ac magnetic susceptibility data measurements were performed using a 4 Oe switching field. All data were corrected for diamagnetic contributions from the eicosane and core diamagnetism estimated using Pascal’s constants^48^. Cole-Cole plots were fitted using formulae describing *χ*′ and *χ*″ in terms of frequency, constant temperature susceptibility (*χ*_T_), adiabatic susceptibility (*χ*_S_), relaxation time (*τ*), and a variable representing the distribution of relaxation times (*α*)^33^. All data were fitted to *α* values of ≤0.09.

### Details for modeling dc magnetic susceptibility data

Magnetic susceptibility data for **1-Dy**, **1-Tb**, **2-Dy**, and **2-Tb** were modeled using the Hamiltonian given in Eq. (1) above, where *J*_Ln-rad_ corresponds to the magnetic exchange between the radical spin and the *J* multiplets of the lanthanide ions^49^. The operator $O_2^0$ assigns a uniaxial anisotropy parameter to the lanthanide *J* multiplets. When a strongly axial doublet ground state of Dy^III^ or Tb^III^ is obtained, its magnetic exchange with an isotropic spin can be assumed to be Ising in nature^50^. The excited state spectrum for a molecule with dominant Ising exchange should correspond to the energies required for different spin flips, $\Delta E = \Delta \left( {2{J_{{\rm{Ln}} - {\rm{rad}}}}\left( {{J_{{\rm{Ln}}\left( 1 \right)}}{S_{{\rm{rad}}}} + {J_{{\rm{Ln}}\left( 2 \right)}}{S_{{\rm{rad}}}}} \right)} \right)$, where Δ*E* reflects both the exchange coupling strength and the change in total angular momentum between the ground state and the spin-flip-generated excited state. Using the values of *J*_Ln–rad_ determined using the Hamiltonian given in Eq. (1), spin-flip energies, Δ*E*, were estimated for comparison with the experimental barriers, *U*_eff_.

### Data availability

Crystallographic data have been deposited in the Cambridge Structural Database as specified above for each compound under the deposition numbers CCDC 1547761-1547775 that is available free of charge from The Cambridge Crystallographic Data Centre via www.ccdc.cam.ac.uk/data_request/cif. All other data can be obtained from the authors on request.

## Electronic supplementary material

Supplementary Information
