# Supplementary material for: Giant coercivity and high magnetic blocking temperatures for N23− radical-bridged dilanthanide complexes upon ligand dissociation
Source: Nat Commun. 2017 Dec 15;8:2144. doi: 10.1038/s41467-017-01553-w (PMC5732206; doi:10.1038/s41467-017-01553-w)
Supplement: Supplementary file 1 — Supplementary Information [file 41467_2017_1553_MOESM1_ESM.pdf]

Supplementary Methods

1. Experimental Section.

The manipulations described below were performed under an inert atmosphere with rigorous exclusion of air and water using Schlenk, vacuum line, and glovebox techniques. Solvents were dried using a commercial solvent purification system from JC Meyer Solvent Systems.[1] Allylmagnesium chloride (2.0 M in THF) was purchased from Sigma-Aldrich and used as received. Anhydrous 1,4-dioxane was purchased from Sigma-Aldrich and used as received. The precursor 1,2,3,4-Tetramethylcyclopentadiene ($Cp^{Me4H}H$) was purchased from Sigma-Aldrich and dried over 4 Å sieves before use. Anhydrous $LnCl_3$ (Ln = Gd, Tb, Dy) was purchased from Sigma-Aldrich and used as received. Potassium bis(trimethylsilyl)amide, $KN[Si(CH_3)_3]_2$, was purchased from Sigma-Aldrich, dissolved in toluene, filtered through Celite and recrystallized from toluene at −35 °C before use. The compounds KCp^{Me4H},[2] and $[HNEt_3][BPh_4]$,[3] were prepared according to literature procedures. Complexes $Cp^{Me4H}{}_2Ln(\eta^3\text{-}C_3H_5)$ and $Cp^{Me4H}{}_2Ln(BPh_4)$ where Ln = Gd, Tb, Dy were prepared according to synthesis route described below, which is similar in a manner to that of $Cp^{Me4H}{}_2Lu(BPh_4)$ or $Cp^{Me4H}{}_2Sc(BPh_4)$.[2,4] Elemental analyses were performed by the Micro-Mass Facility at the University of California, Berkeley, using a Perkin-Elmer Series 2400 Series II combustion analyzer. IR spectra were recorded on a Perkin-Elmer Avatar Spectrum 400 FTIR Spectrometer equipped with ATR.

$Cp^{Me4H}{}_2$GdAllyl. Allylmagnesium chloride (0.73 mL of a 2.0 M solution in THF) was added to $Cp^{Me4H}{}_2GdCl_2K$ (0.742 g, 1.46 mmol) in 20 mL of toluene to give an orange solution. After stirring for 4 hours, the solution was evaporated to dryness to yield an orange solid. This solid was triturated with 2% 1,4-dioxane in hexanes (20 mL), and white insolubles were removed by filtration to yield a yellow solution. Removal of the solvent under vacuum afforded a crystalline material, $Cp^{Me4H}{}_2Gd(\eta^3\text{-}C_3H_5)$, (0.562 g, 88%). Crystals suitable for X-ray analysis were grown from a concentrated hexane solution of $Cp^{Me4H}{}_2Gd(\eta^3\text{-}C_3H_5)$ at −35 °C over the course of 2d. IR (neat): Anal. Calcd for $C_{21}H_{31}Gd$: C, 57.23; H, 7.09. Found: C, 57.18; H, 7.18.

$Cp^{Me4H}{}_2Gd(BPh_4)$. $[HNEt_3][BPh_4]$ (0.537 g, 1.28 mmol) was slowly added to a stirred solution of $Cp^{Me4H}{}_2Gd(\eta^3\text{-}C_3H_5)$ (0.562 g, 1.28 mmol) in 20 mL of toluene. After the solution was stirred for six hours, the solution was evaporated to dryness. The obtained solid was washed twice with minimum amount of toluene to afford a pale yellow powder (0.790 g, 86%). Crystals suitable for X-ray analysis were grown from a hot toluene solution slowly cooled to room temperature. Anal. Calcd for $C_{42}H_{46}BGd\cdot0.5C_7H_8$: C, 71.44; H, 6.59. Found: C, 71.94; H, 6.55.

$Cp^{Me4H}{}_2$TbAllyl. Allylmagnesium chloride (0.69 mL of a 2.0 M solution in THF) was added to $Cp^{Me4H}{}_2TbCl_2K$ (0.706 g, 1.38 mmol) in 20 mL of toluene to give an orange solution. After stirring for 4 hours, the solution was evaporated to dryness to yield an orange solid. This solid was triturated with 2% 1,4-dioxane in hexanes (20 mL), and white insolubles were removed by filtration to yield a yellow solution. Removal of the solvent under vacuum afforded a crystalline material, $Cp^{Me4H}{}_2Tb(\eta^3\text{-}C_3H_5)$, (0.508 g, 83%). Crystals suitable for X-ray analysis were grown from a concentrated hexane solution of $Cp^{Me4H}{}_2Tb(\eta^3\text{-}C_3H_5)$ at −35 °C over the course of 2d. IR (neat): Anal. Calcd for $C_{21}H_{31}Tb$: C, 57.01; H, 7.06. Found: C, 57.10; H, 6.99.

$Cp^{Me4H}{}_2Tb(BPh_4)$. $[HNEt_3][BPh_4]$ (0.484 g, 1.15 mmol) was slowly added to a stirred solution of $Cp^{Me4H}{}_2Tb(\eta^3\text{-}C_3H_5)$ (0.508 g, 1.15 mmol) in 20 mL of toluene. After the solution was stirred for six hours, the solution was evaporated to dryness. The obtained solid was washed twice with minimum amount of toluene to afford a pale yellow powder (0.753g, 91%). Crystals suitable for X-ray analysis were grown from a hot toluene solution slowly cooled to room temperature. Anal. Calcd for $C_{42}H_{46}BTb\cdot C_7H_8$: C, 72.42; H, 6.70. Found: C, 72.18; H, 6.88.

$Cp^{Me4H}{}_2$DyAllyl. Allylmagnesium chloride (0.64 mL of a 2.0 M solution in THF) was added to $Cp^{Me4H}{}_2DyCl_2K$ (663.7 mg, 1.289 mmol) in 20 mL of toluene to give a yellow solution. After stirring for 4 hours, the solution was evaporated to dryness to yield a yellow solid. This solid was triturated with 2% 1,4-dioxane in hexanes (20 mL), and white insolubles were removed by filtration to yield a yellow solution. Removal of the solvent under vacuum afforded a crystalline material, $Cp^{Me4H}{}_2Dy(\eta^3\text{-}C_3H_5)$, (462.4 mg, 80%). Crystals suitable for X-ray analysis were grown from a concentrated hexane solution of $Cp^{Me4H}{}_2Dy(\eta^3\text{-}C_3H_5)$ at −35 °C over the course of 2d. IR (neat): 3064w, 2960m, 2900s, 2854s, 2754s, 2724m, 1630w, 1539s, 1439sbr, 1380s, 1324m, 1233s, 1174w, 1021s, 970m, 917w, 818s, 774vs, 720m, 694w, 667s, 634s, 627s, 613s cm^{-1}. Anal. Calcd for $C_{21}H_{31}Dy$: C, 56.56; H, 7.01. Found: C, 56.65; H, 7.08.

$Cp^{Me4H}{}_2DyBPh_4$. $[HNEt_3][BPh_4]$ (436.9 mg, 1.036 mmol) was slowly added to a stirred solution of $Cp^{Me4H}{}_2Dy(\eta^3\text{-}C_3H_5)$ (462.4 mg, 1.036 mmol) in 20 mL of toluene. After the solution was stirred for six hours, the solution was evaporated to dryness. The obtained solid was washed twice with minimum amount of toluene to afford a pale yellow powder (648.4 mg, 86%). Crystals suitable for X-ray analysis were grown from a hot toluene solution slowly cooled to room temperature. IR (neat): 3061m, 3047m, 3008m, 2969m, 2904s, 2857m, 1807w, 1603w, 1585m, 1577m, 1555w, 1494m, 1475s, 1426s, 1384s, 1374s, 1322m, 1268s, 1259s, 1238m, 1181m, 1146s, 1131m, 1109w, 1067w, 1056m, 1031s, 1022s, 998w, 968w, 929w, 878s, 848s, 799s, 749s, 740s, 732vs, 721s, 701vs, 666s, 658s, 641s, 623s, 612s cm^{-1}. Anal. Calcd for $C_{42}H_{46}BDy$: C, 70.01; H, 6.43. Found: C, 70.06; H, 6.44.

2. Arrhenius Plot Fitting Details.

The temperature dependence of the magnetic relaxation times recorded on sample of **1-Dy, 1-Tb, 2-Dy**, and **2-Tb** were analyzed in terms of the contributions of different relaxation processes to the observed relaxation rates. Herein we give the details related to the fitting and interpretation of the results, though the reader is referred to the main text for a broader discussion. The curvature of the plots suggested the occurance of multiple relaxation processes, thus many fits were tried with different incorporated relaxation pathways depending on the sample measurement conditions.

The typical magnetic relaxation pathways discussed in the literature, along with their dependence on temperature, are the following: a temperature independent quantum tunneling pathway, Direct relaxation ($\propto T$ or T^2), the Raman relaxation process ($\propto T^n$, n = 4, 5, 7, or 9 typically), and the Orbach process ($\propto \exp(U_{eff}/k_BT)$).[5,6] Note that successful modeling of the Arrhenius plots for **1-Dy, 1-Tb, 2-Dy**, and **2-Tb** did not require each term, and some were judiciously excluded from the fits depending on the nature of the sample. For example, the data collected on all compounds, at zero dc field were not modeled with the Direct process since the corresponding contribution is nullified in the absence of a dc field. The inclusion of a Raman process did not improve the quality of the fit for **1-Dy** and **2-Tb**.

Fitting detail for compound 1-Dy

The Arrhenius data for **1-Dy** were modeled using equation (1), and the resulting best-fit parameters are given in Supplementary Table 1.

$$\frac{1}{\tau_{obs}} = \frac{1}{\tau_{QTM}} + \tau_0^{-1} exp\left({}^{-U_{eff}}\Big/_{k_B T}\right) \qquad (1)$$

Here, the first term is from the tunneling pathway and the second models an Orbach relaxation pathway. Below 7 K the Arrhenius plot for **1-Dy** is nearly temperature independent, which is suggestive of dominant tunneling behavior. Therefore, acceptable fits were obtained by utilizing the first term in equation (1) (see Supplementary Figure 32).

Fitting detail for compound 1-Tb and 2-Dy

The Arrhenius data for **1-Tb** and **2-Dy** containing data obtained from ac measurements (see Supplementary Figures 37 and 43) were obtained from a linear fit to the Arrhenius expression for the relaxation time:

$$\ln(\tau) = \ln(\tau_0) + U_{\mathrm{eff}}/k_{\mathrm{B}}T. \qquad (2)$$

The Arrhenius data for **1-Tb** containing data obtained from ac measurements and dc relaxation experiments were modeled using equation (1), and the resulting best-fit parameters are given in Supplementary Table 1 (see Supplementary Figure 39).

The Arrhenius plot for **2-Dy**, composed from relaxation time data obtained from ac measurements and dc relaxation experiments, was modeled using equation (3) and the resulting best-fit parameters are given in Supplementary Table 1.

$$\frac{1}{\tau_{obs}} = \frac{1}{\tau_{QTM}} + CT^n + \tau_0^{-1} exp\left({}^{-U_{eff}}\!\middle/_{\mathrm{k_B}T}\right) \qquad (3)$$

Here, the first term is from the tunneling pathway, the second is for the Raman process, and the third term models Orbach relaxation pathway (see Supplementary Figure 45).

Fitting detail for compound 2-Tb

The Arrhenius plot for **2-Tb** (see Supplementary Figure 49) was constructed from relaxation times obtained from ac measurements where this data modeled using equation (4), and the resulting best-fit parameters are given in Supplementary Table 1.

$$\frac{1}{\tau_{obs}} = \tau_{0,1}^{-1} exp\left({}^{-U_{eff,1}}\!\middle/_{\mathrm{k_B}T}\right) + \tau_{0,2}^{-1} exp\left({}^{-U_{eff,2}}\!\middle/_{\mathrm{k_B}T}\right) \qquad (4)$$

Here, the two terms model two Orbach relaxation pathways.

The Arrhenius data for **2-Tb** (shown as Supplementary Figure 56) where the relaxation times were obtained from ac measurements and dc relaxation measurements were modeled using equation (5), and the resulting best-fit parameters are given in Supplementary Table 1.

$$\frac{1}{\tau_{obs}} = \frac{1}{\tau_{QTM}} + \tau_{0,1}^{-1} exp\left({}^{-U_{eff,1}}\!\middle/_{\mathrm{k_B}T}\right) + \tau_{0,2}^{-1} exp\left({}^{-U_{eff,2}}\!\middle/_{\mathrm{k_B}T}\right) \qquad (5)$$

Here, the first term is from the tunneling pathway, the second and third term model two Orbach relaxation pathways. Below 14 K, the Arrhenius plot for **2-Tb** is nearly temperature independent, which is suggestive of dominant tunneling behavior. Therefore, acceptable fits were obtained by utilizing the first term in equation (5) (see Supplementary Figure 56).

3. Details of fitting data from dc relaxation experiments for 1-Tb, 2-Dy and 2-Tb

The data from dc relaxation experiments for **1-Tb**, **2-Dy** and **2-Ln** were fitted to a function of the form $y = a \cdot \exp(-((t/\tau)^b))$ where b is a stretch factor. The fits of the dc relaxation experiments at various temperatures gave the τ (s) and b values listed in Supplementary Tables 2-4. The corresponding dc relaxation experiments are shown in Supplementary Figures 38, 44, 50-55.

4. Details for models of $\chi_M T$ data for 1-Tb, 1-Dy, 2-Tb, and 2-Dy.

Data were modeled using the Hamiltonian:

$$\widehat{H} = -2J_{Ln-rad}\hat{S}_{rad} \cdot \left(\hat{J}_{Ln(1)} + \hat{J}_{Ln(2)}\right) + \sum_{i=Ln(1),Ln(2)} B_2^0\, O_2^0(i)$$

in which J_{Ln-rad} corresponds to the magnetic exchange between the radical spin and the J multiplets of the lanthanide ions. The operator O_2^0 assigns a uniaxial anisotropy parameter to the lanthanide J multiplets. The uniaxial anisotropy B_2^0 of the lanthanide centers was assumed to be large in comparison to the magnitude of magnetic exchange. The value used for the B_2^0 parameter in each model was -150 cm^{-1}, though variations of this parameter to values as high as -800 cm^{-1} did not alter the models significantly.

In some cases for later lanthanide-containing systems, including for Tb^{3+} and Dy^{3+}, when a strongly axial doublet ground state of the Ln^{3+} ion is obtained, the magnetic exchange of the total angular momentum of the Ln^{3+} ion with an isotropic spin can be assumed to be Ising in nature.[7] The excited state spectrum for a molecule with dominant Ising exchange corresponds to the energies required for different spin flips:

$$\Delta E = \Delta(2J_{Ln-rad}(J_{Ln1}S_{rad} + J_{Ln2}S_{rad}))$$

where ΔE reflects both the exchange coupling strength and the change in total angular momentum between the ground state (or whichever state from which the spin-flip excitation is occurring) and the spin-flip-generated excited state.

As an example, in **2-Tb**, the first excited state corresponds to a flip of one terbium moment, with an energy of $(2J_{Ln-rad})(6)$, or $12J_{Ln-rad}$, while the second excited state energy corresponds to a flip of the radical spin, with an energy of $(2J_{Ln-rad})(12)$, or $24J_{Ln-rad}$ (here the change in angular momenta is between those of the ground and second excited states, rather than between the ground and first excited states).

The Landé g values for the lanthanide J multiplets were allowed to vary around their expected g_J values, 1.50 for Tb^{3+} and 1.33 for Dy^{3+}. Variations from expected g_J values may have a variety of meanings. In the case of **1-Tb**, the extremely small variations above 1.50 may potentially be attributed to a small sample mass error, as g_J effectively acts as a scaling parameter in these models. For **2-Tb**, the lower than expected g values that provide the best fits may be attributed to a contribution from a coupled higher angular momentum state with a g value below 1.5.[8] Such a contribution is possible even at room temperature due to the strong lanthanide-radical coupling. In contrast, the larger than expected g values that enable the best fits for **1-Dy** and **2-Dy** are more challenging to rationalize. While mass error is not impossible, the larger deviations

from expected g_J for both **1-Dy** and **2-Dy** suggest a more complex origin. Fortunately, J_{Ln-rad} mostly prompts changes in slope of $\chi_M T$ with changing temperature rather than scaling of the $\chi_M T$ product. Since the model tracks changes in slope with decreasing temperature for **1-Dy** and **2-Dy** reasonably well, the J_{Ln-rad} values extracted may be considered reasonable, though certainly not definitive.

A number of models with small variations of g_J and J_{Ln-rad} can reasonably reproduce the data for **1-Dy**, **1-Tb**, **2-Dy,** and **2-Tb**. As such, multiple models are shown for each $\chi_M T$ versus T data set. For each data set, the model represented by a blue dashed line reflects g_J held constant at its expected value, with J_{Ln-rad} allowed to vary to provide the best fit. The model shown as a solid purple line reflects a J_{Ln-rad} value, held constant, that generates a spin-flip barrier that best corresponds to the experimental U_{eff} observed for that complex, with g_J then allowed to vary to provide the best fit. Finally, the model represented by a pink dash-dot line reflects a model in which both J_{Ln-rad} and g_J are allowed to vary to generate the best fit to the total data set. Each of these models was generated using the program PHI[9], with experimental $\chi_M T$ data collected across a temperature range of 20 – 300 K, Supplementary Figures 58-59, Supplementary Tables 5 and 6).

5. Crystallographic data

X-ray diffraction experiments were performed at 100 K on crystals coated with Paratone-N oil and mounted on Kapton or MiTeGen loops. X-ray data were collected at the Small Molecule X-ray Crystallography Facility at the University of California, Berkeley using a Bruker QUAZAR diffractometer equipped with a microfocus sealed X-ray source (Mo Kα radiation; λ = 0.71073 Å) and a Bruker APEX-II detector (for **2-Tb**, **4**, **3-Dy**, $Cp^{Me4H}{}_2Dy(BPh_4)$, $Cp^{Me4H}{}_2Gd(BPh_4)$, $Cp^{Me4H}{}_2$TbAllyl, and $Cp^{Me4H}{}_2$DyAllyl) or at Beamline 11.3.1 at the Advanced Light Source on a Bruker D8 Diffractometer equipped with a Bruker PHOTON100 CMOS detector using synchrotron radiation (λ = 0.6888 Å for $Cp^{Me4H}{}_2$GdAllyl and **1-Dy**; λ = 0.7749 Å for **2-Dy**, **1-Tb**, **1-Gd**, **3-Tb**, **3-Gd**, and $Cp^{Me4H}{}_2Tb(BPh_4)$). Crystals for **2-Tb**, **2-Dy**, and **1-Gd** were found to be non-merohedral twins based on analysis of their diffraction patterns. For each of these structures, CELL_NOW[10] was used to determine the orientation matrices and raw data for both twin matrices were integrated and corrected for Lorentz and polarization effects using Bruker AXS SAINT[11] software and corrected for absorption using TWINABS.[12] For all other structures, raw data were integrated and corrected for Lorentz and polarization effects using Bruker AXS SAINT[11] software and corrected for absorption using SADABS.[13] Space group assignments were determined by examination of systematic absences, E-statistics, and successive refinement of the structures. All structures were solved by intrinsic phasing using SHELXT.[14] Additional refinement was performed with SHELXL[15] operated within the OLEX2[16] interfaces. Disorder in the structures of **2-Tb**, **2-Dy**, **1-Tb**, **1-Dy**, **1-Gd**, **3-Tb**, **3-Dy**, and **3-Gd** required the use of displacement parameter restraints in the refinement. The structures for **1-Dy**, **1-Gd**, **3-Tb**, **3-Gd**, and $Cp^{Me4H}{}_2$GdAllyl gave rise to A and B level alerts from checkCIF. Responses addressing these alerts have been included in the CIFs and can be read in reports generated by checkCIF. Further details are provided in Supplementary Tables 7-9.

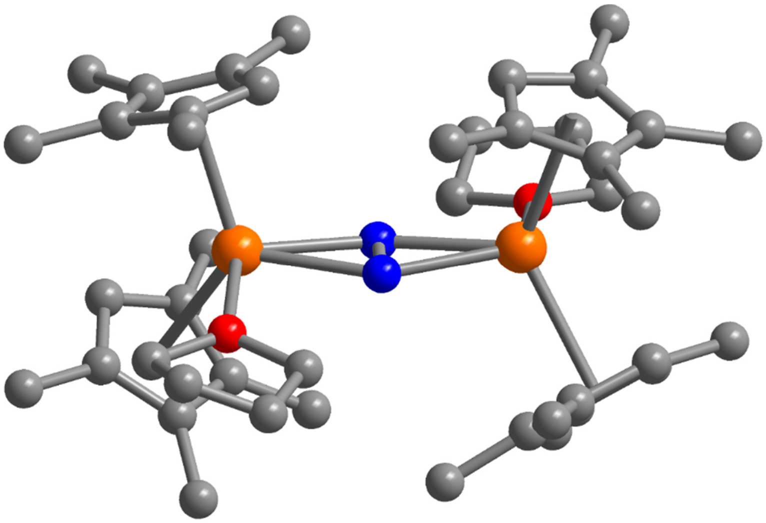

Supplementary Figure 1. Structure of the N_2^{3-} radical-bridged anion in complex **1-Gd**. Orange, red, blue, and gray spheres represent Gd, O, N, and C atoms, respectively; H atoms have been omitted for clarity.

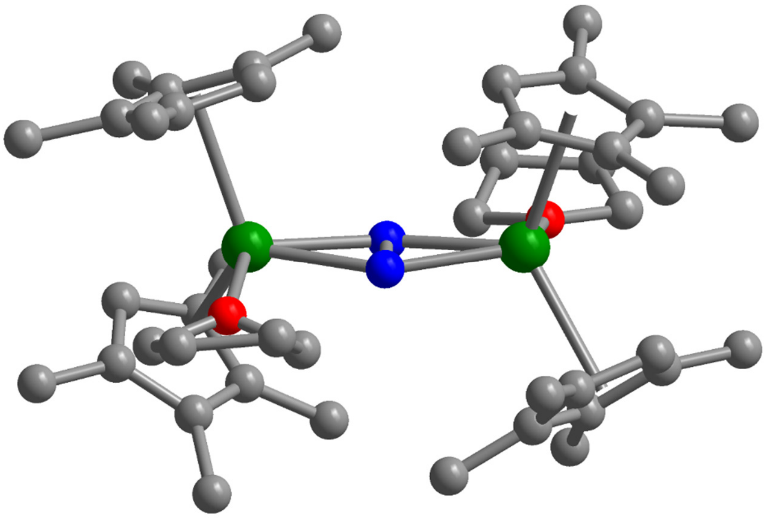

Supplementary Figure 2. Structure of the N_2^{3-} radical-bridged anion in complex **1-Dy**. Green, red, blue, and gray spheres represent Dy, O, N, and C atoms, respectively; H atoms have been omitted for clarity.

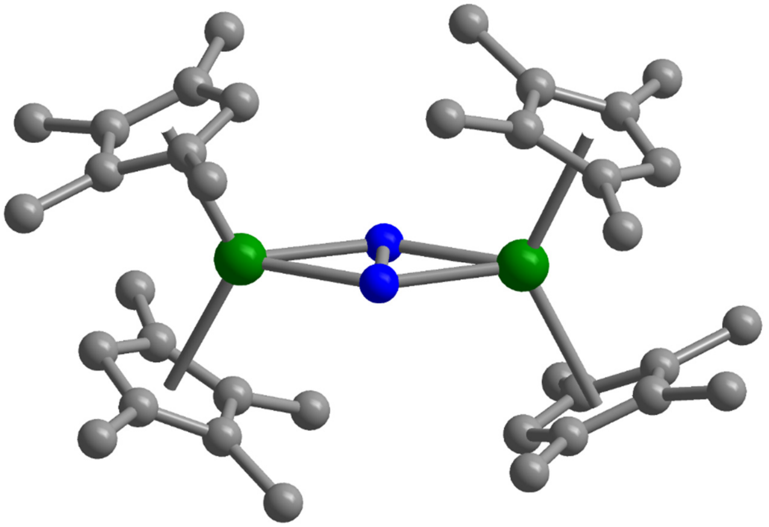

Supplementary Figure 3. Structure of the N_2^{3-} radical-bridged complex in **2-Dy**. Orange, red, blue, and gray spheres represent Dy, O, N, and C atoms, respectively; H atoms have been omitted for clarity.

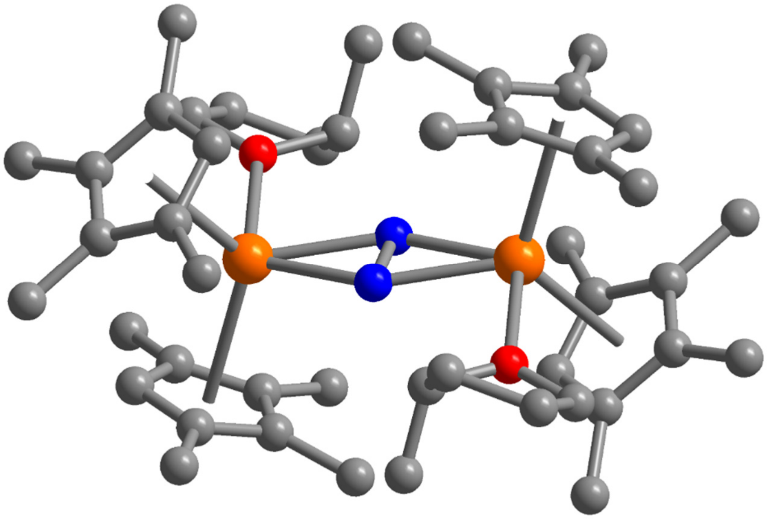

Supplementary Figure 4. Structure of the N_2^{3-} radical-bridged anion in complex **4**. Orange, red, blue, and gray spheres represent Gd, O, N, and C atoms, respectively; H atoms have been omitted for clarity.

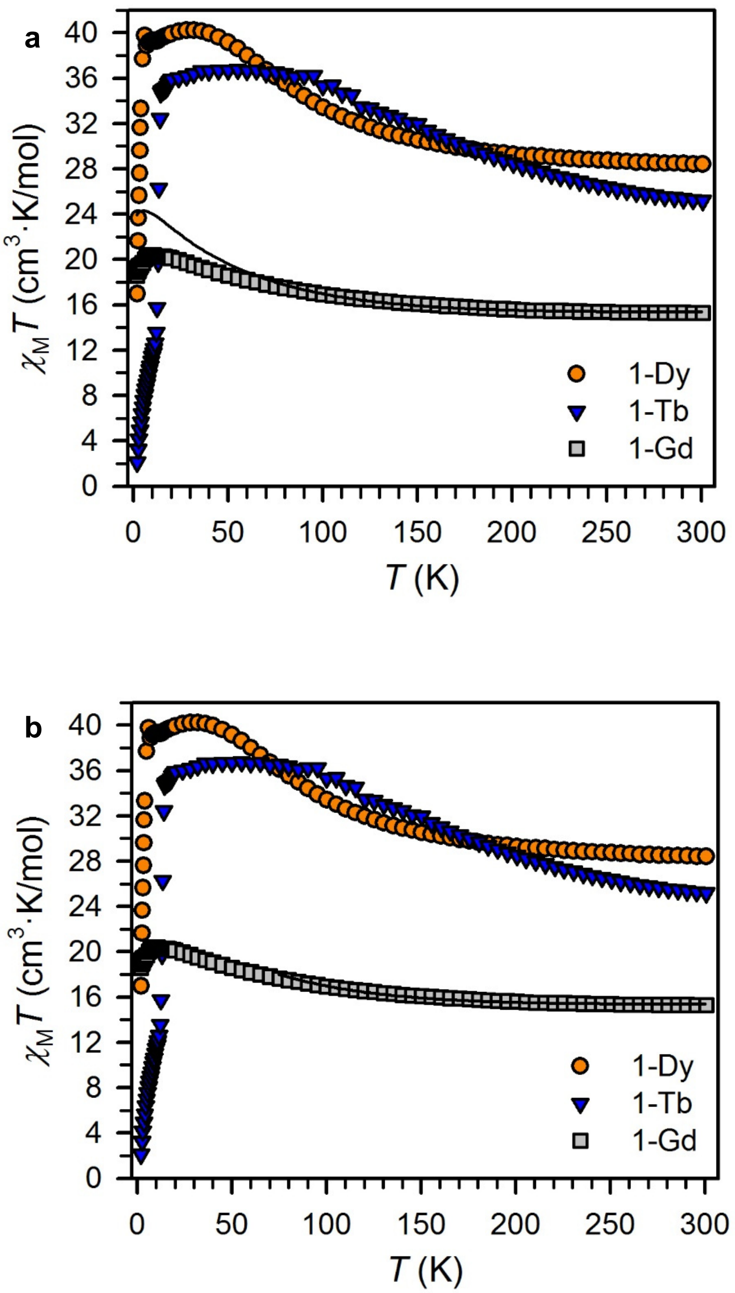

Supplementary Figure 5. Variable temperature dc susceptibility data of polycrystalline **1-Dy** (orange circles), **1-Tb** (blue triangles) and **1-Gd** (grey squares) collected under 0.1 T applied dc field. The black line represents a fit to the data for **1-Gd**, as discussed in the main text. In **a** the fit is shown from 2 to 300 K. In **b** the fit is shown from 75 to 300 K for clarity.

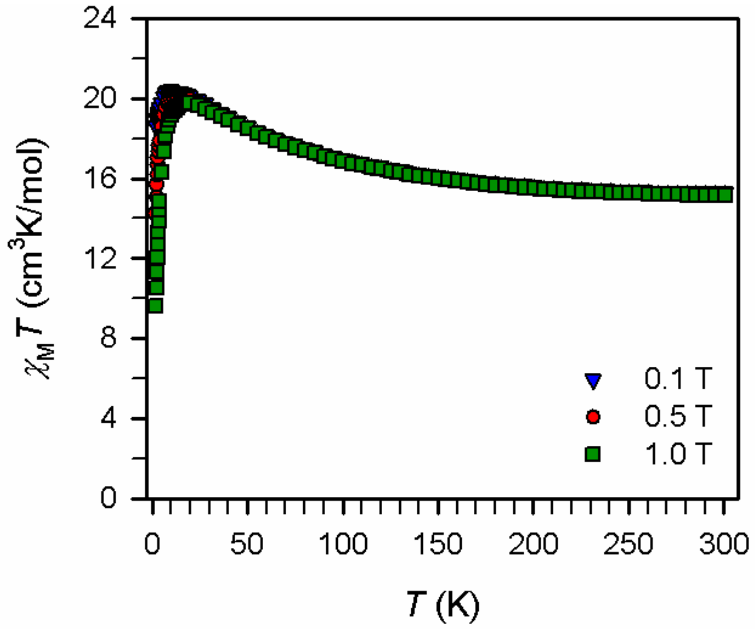

Supplementary Figure 6. Variable-temperature dc magnetic susceptibility data for a restrained polycrystalline sample of **1-Gd** collected under a 0.1 T (blue triangles), 0.5 T (red circles), 1 T (green squares) applied dc field.

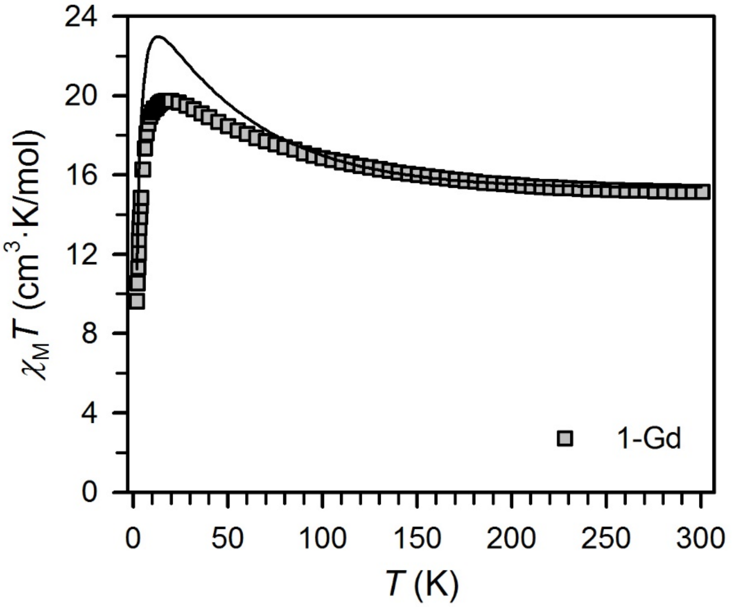

Supplementary Figure 7. Variable-temperature dc magnetic susceptibility data for a restrained polycrystalline sample of **1-Gd** collected under a 1 T applied dc field. The black lines represents a fit to the data for **1-Gd**, as discussed in the main text.

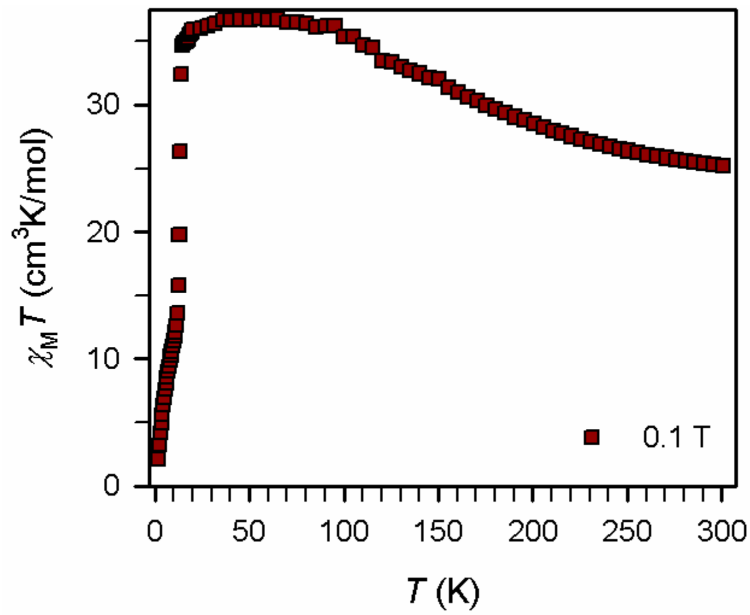

Supplementary Figure 8. Variable temperature dc susceptibility data of polycrystalline **1-Tb** collected under 0.1 T applied dc field.

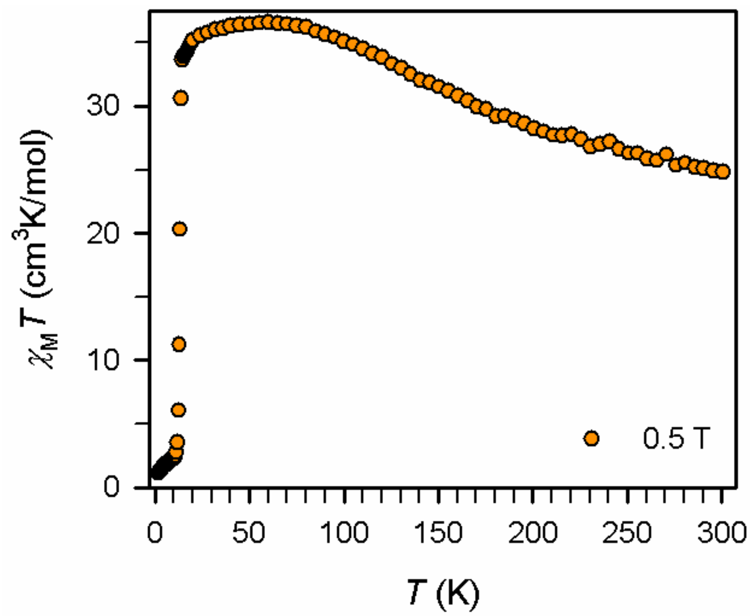

Supplementary Figure 9. Variable temperature dc susceptibility data of polycrystalline **1-Tb** collected under 0.5 T applied dc field.

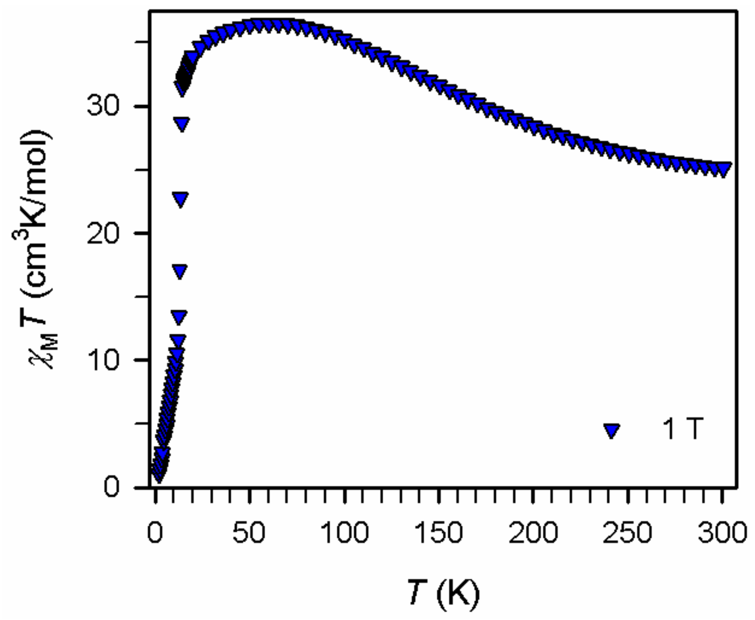

Supplementary Figure 10. Variable temperature dc susceptibility data of polycrystalline **1-Tb** collected under 1 T applied dc field.

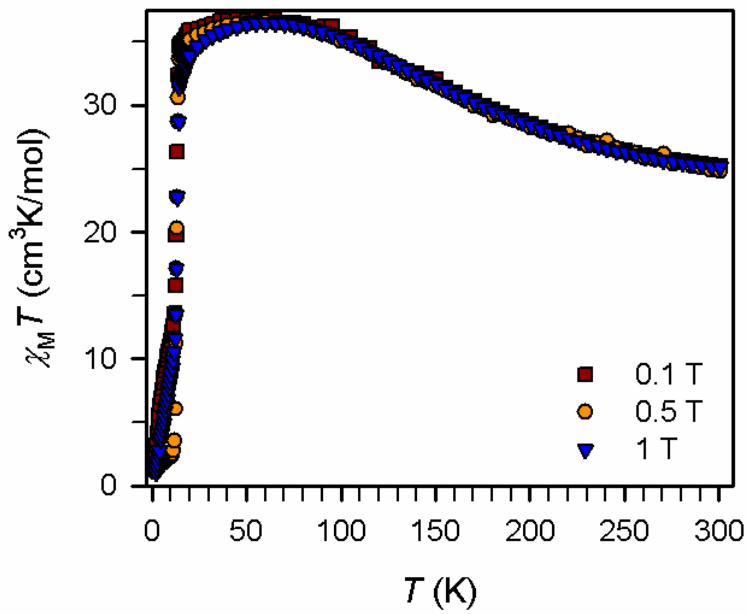

Supplementary Figure 11. Variable temperature dc susceptibility data of polycrystalline **1-Tb** collected under 0.1 T (dark red squares), 0.5 T (orange circles), 1 T (blue triangles) applied dc field.

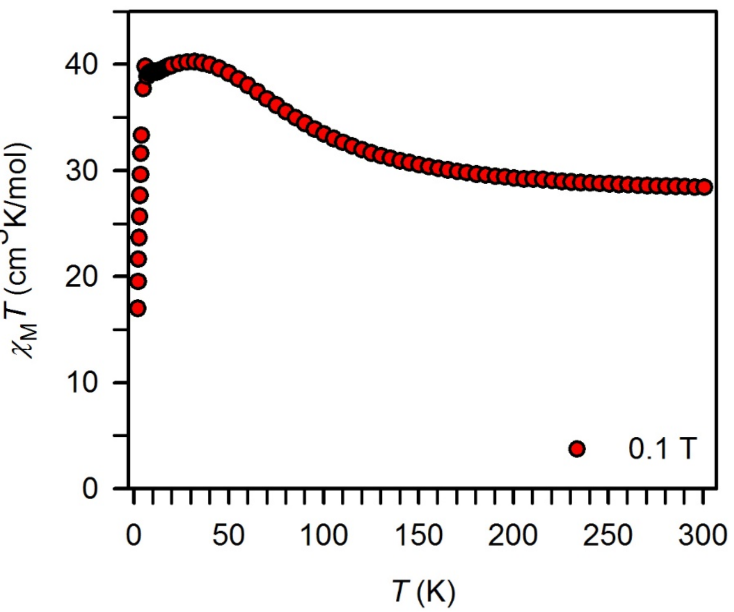

Supplementary Figure 12. Variable-temperature dc magnetic susceptibility data for a restrained polycrystalline sample of **1-Dy** collected under a 0.1 T applied dc field.

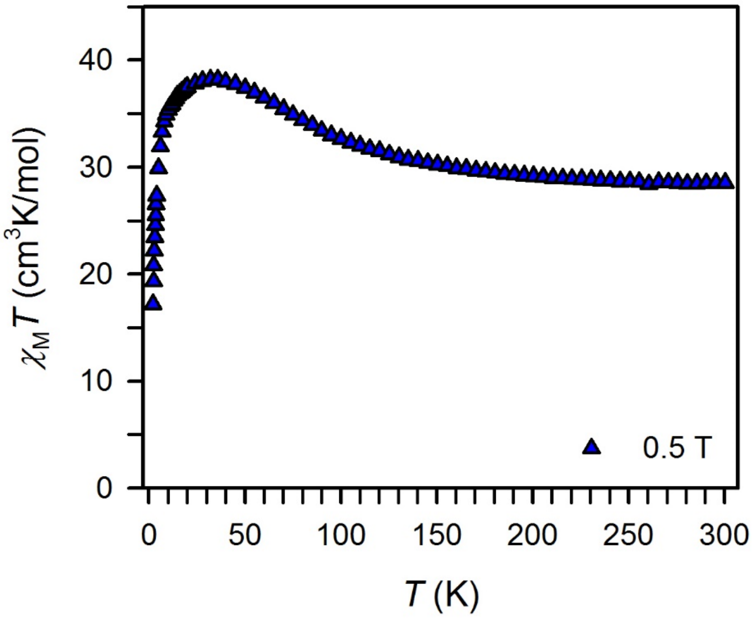

Supplementary Figure 13. Variable-temperature dc magnetic susceptibility data for a restrained polycrystalline sample of **1-Dy** collected under a 0.5 T applied dc field.

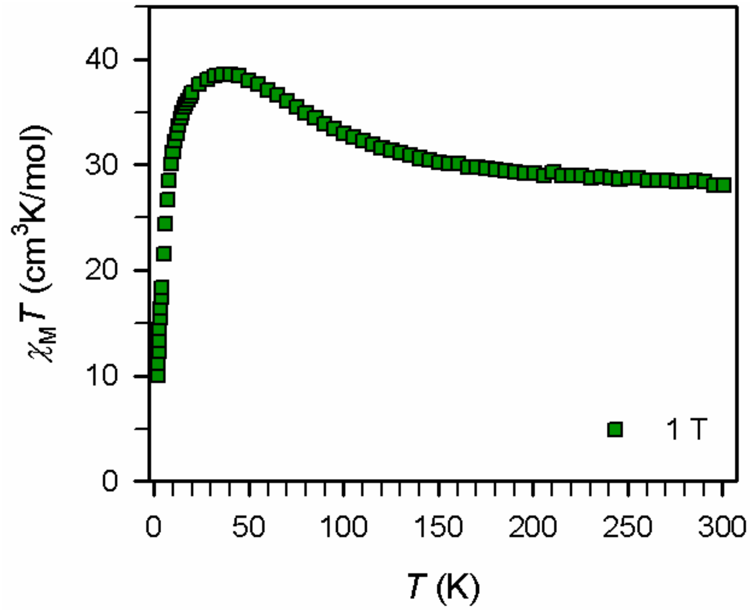

Supplementary Figure 14. Variable-temperature dc magnetic susceptibility data for a restrained polycrystalline sample of **1-Dy** collected under a 1 T applied dc field.

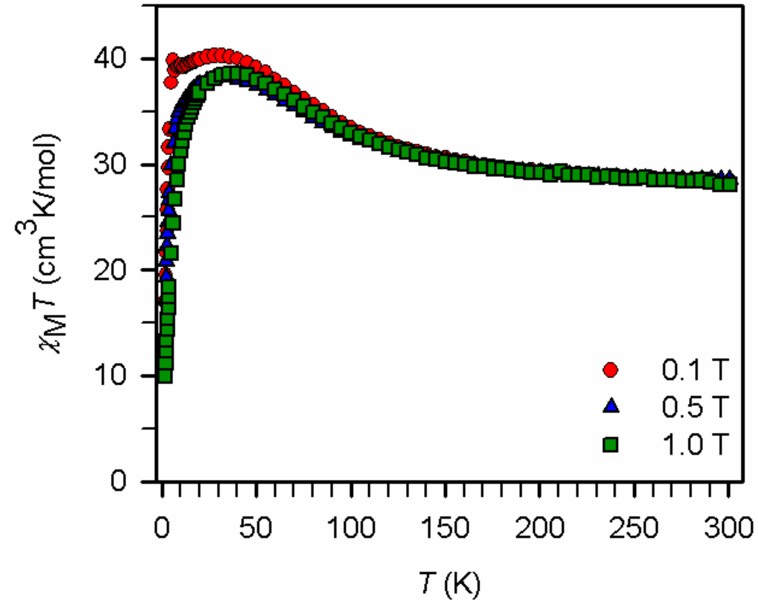

Supplementary Figure 15. Variable temperature dc susceptibility data of polycrystalline **1-Dy** collected under 0.1 T (red circles), 0.5 T (blue triangles), 1 T (green squares) applied dc field.

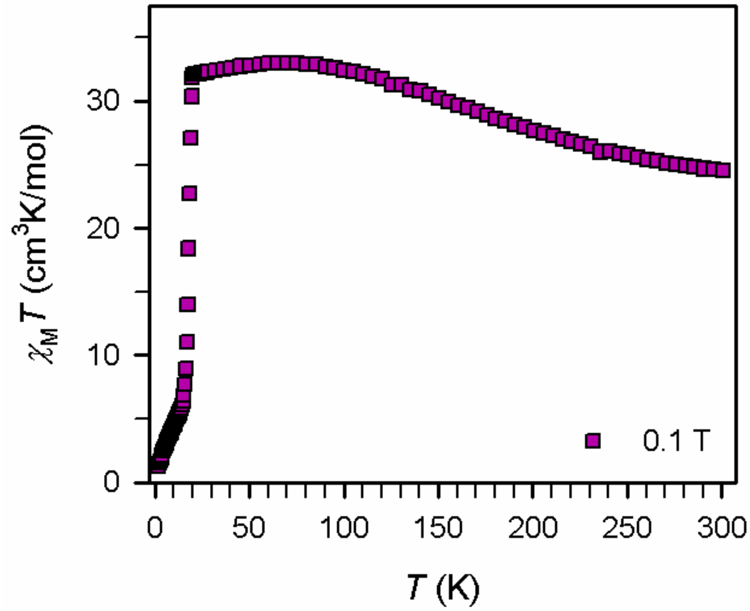

Supplementary Figure 16. Variable temperature dc susceptibility data of polycrystalline **2-Tb** collected under 0.1 T applied dc field.

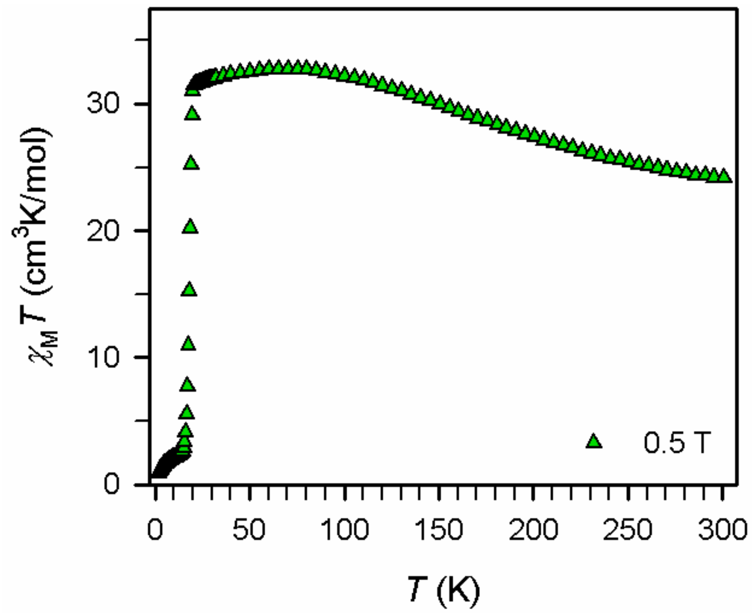

Supplementary Figure 17. Variable temperature dc susceptibility data of polycrystalline **2-Tb** collected under 0.5 T applied dc field.

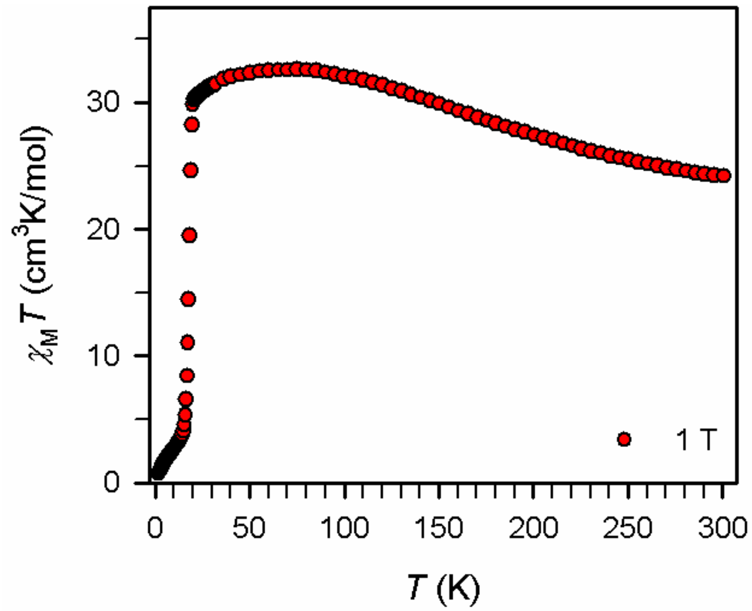

Supplementary Figure 18. Variable temperature dc susceptibility data of polycrystalline **2-Tb** collected under 1 T applied dc field.

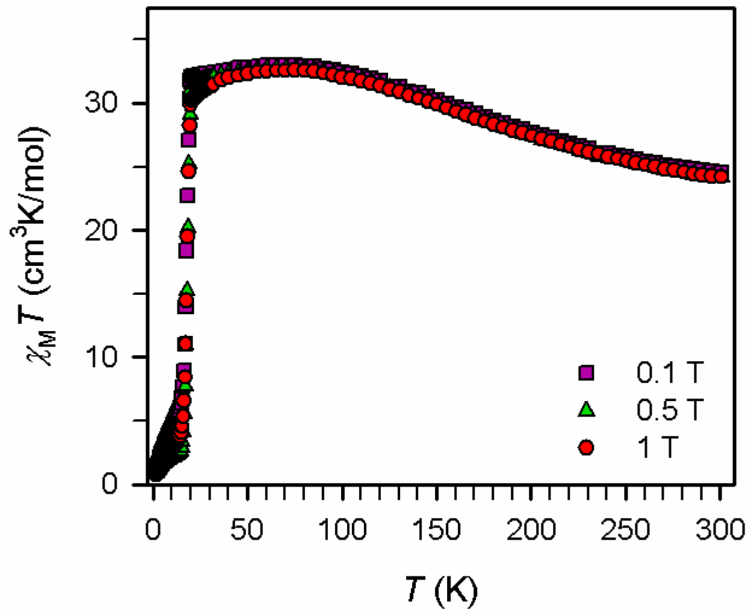

Supplementary Figure 19. Variable temperature dc susceptibility data of polycrystalline **2-Tb** collected under 0.1 T (purple squares), 0.5 T (green triangles), 1 T (red circles) applied dc field.

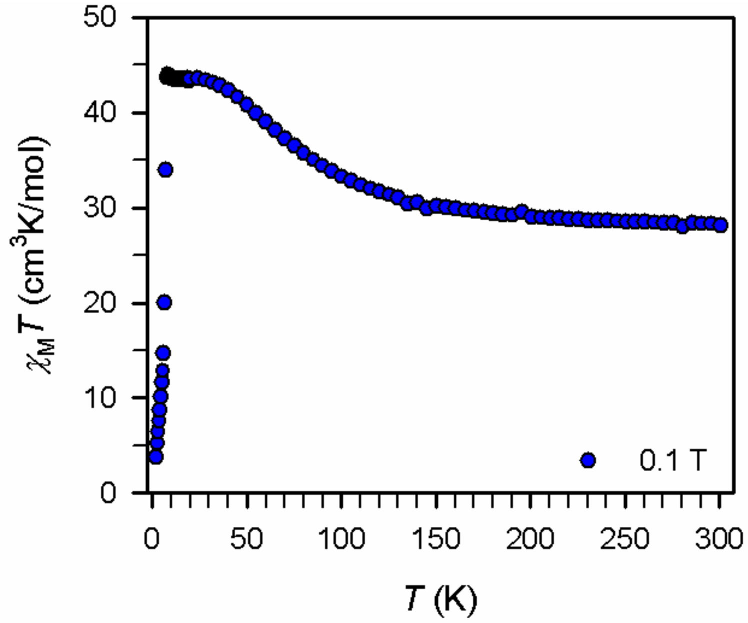

Supplementary Figure 20. Variable temperature dc susceptibility data of polycrystalline **2-Dy** collected under 0.1 T applied dc field.

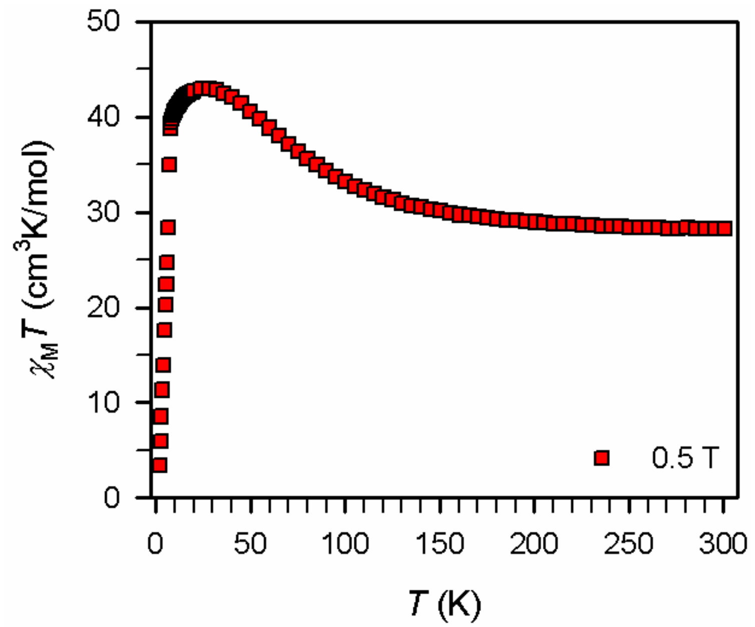

Supplementary Figure 21. Variable temperature dc susceptibility data of polycrystalline **2-Dy** collected under 0.5 T applied dc field.

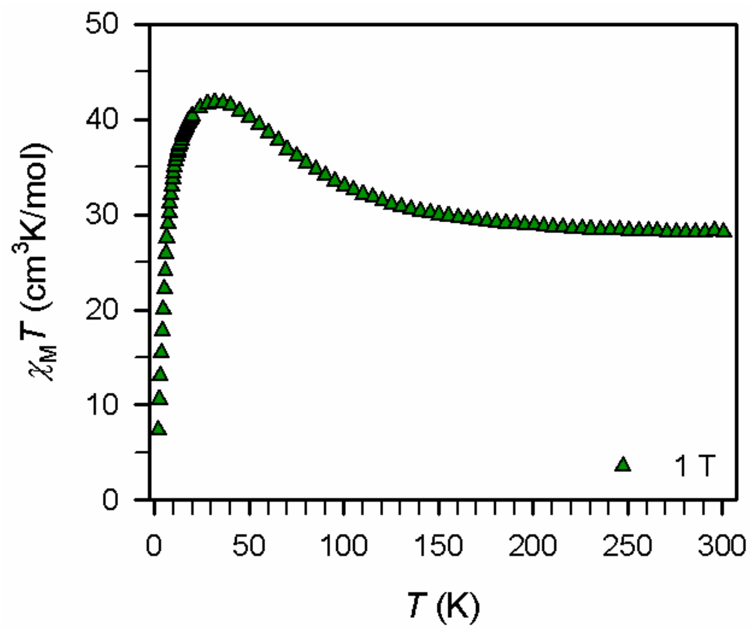

Supplementary Figure 22. Variable temperature dc susceptibility data of polycrystalline **2-Dy** collected under 1 T applied dc field.

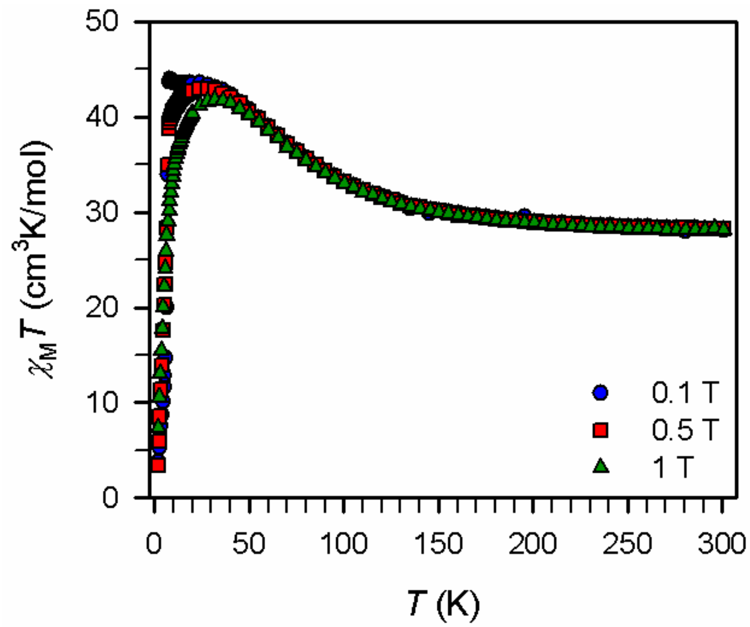

Supplementary Figure 23. Variable temperature dc susceptibility data of polycrystalline **2-Dy** collected under 0.1 T (blue circles), 0.5 T (red squares), 1 T (green triangles) applied dc field.

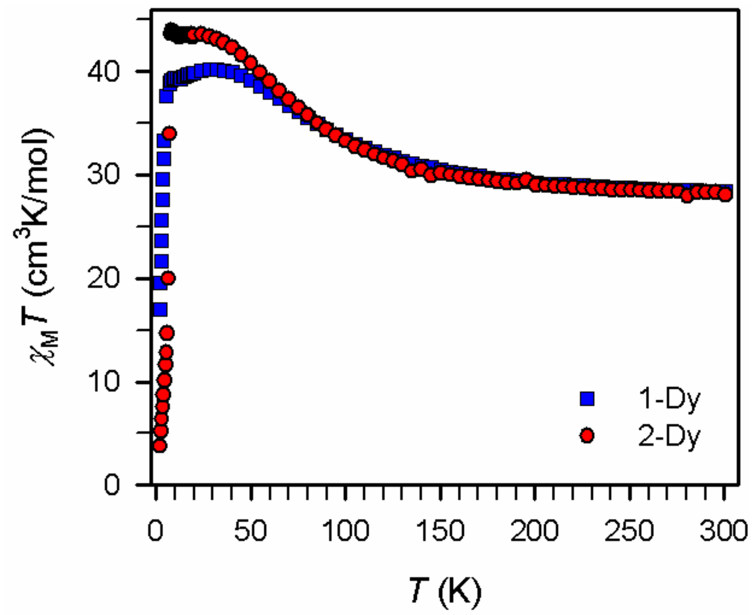

Supplementary Figure 24. Comparison of variable temperature dc susceptibility data of polycrystalline **1-Dy** (blue squares) and **2-Dy** (red circles) collected under 0.1 T applied dc field.

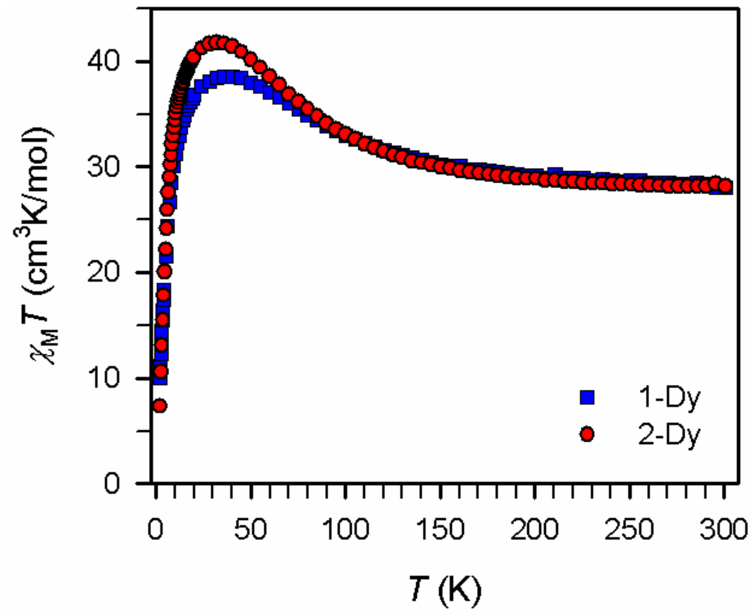

Supplementary Figure 25. Comparison of variable temperature dc susceptibility data of polycrystalline **1-Dy** (blue squares) and **2-Dy** (red circles) collected under 1 T applied dc field.

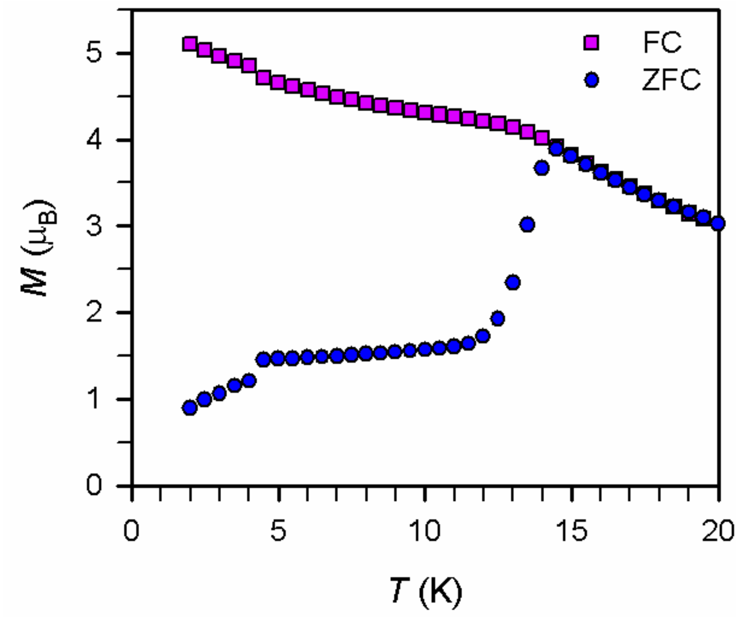

Supplementary Figure 26. Field cooled (purple squares) and zero-field cooled (blue circles) magnetization versus temperature for **1-Tb**, collected under an applied field of 1 T, revealing a blocking temperature of 14.5 K.

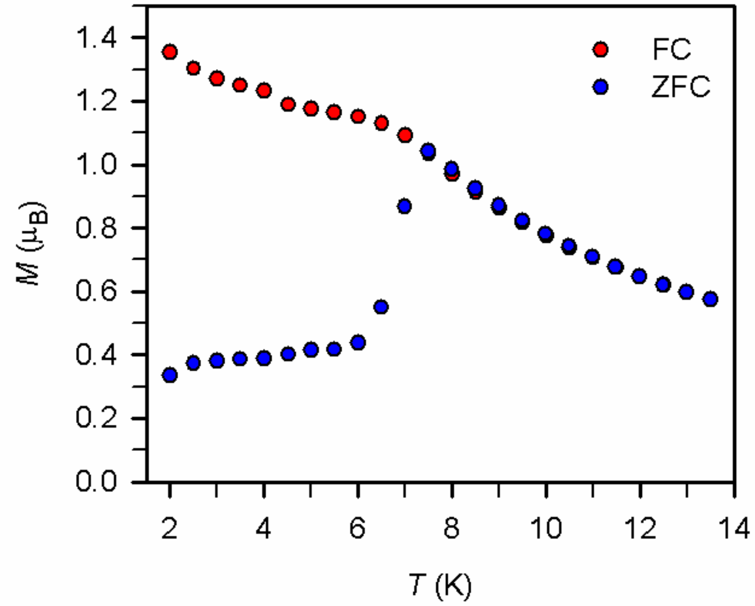

Supplementary Figure 27. Field cooled (red circles) and zero-field cooled (blue circles) magnetization versus temperature for **2-Dy**, collected under an applied field of 0.1 T, revealing a blocking temperature of 7.5 K.

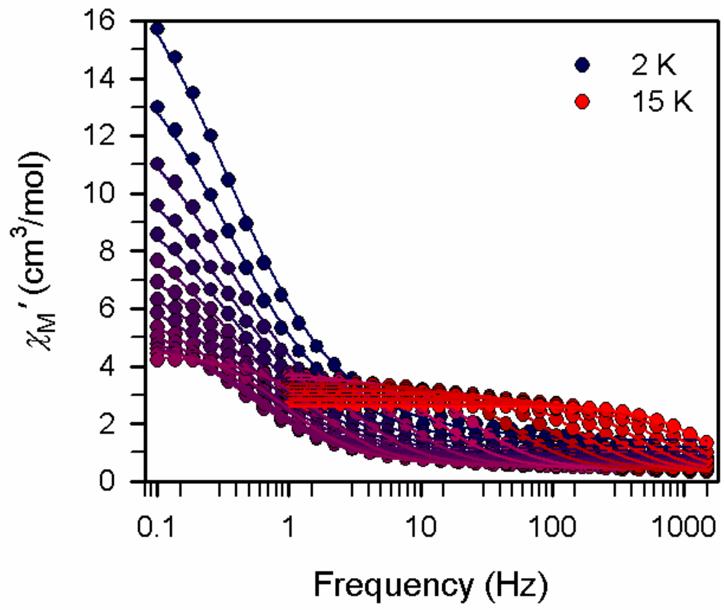

Supplementary Figure 28. In-phase (χ_M') components of the ac magnetic susceptibility for **1-Dy** under zero applied dc field from 2 K (dark blue circles) to 15 K (red circles). Solid lines represent a fit to the data.

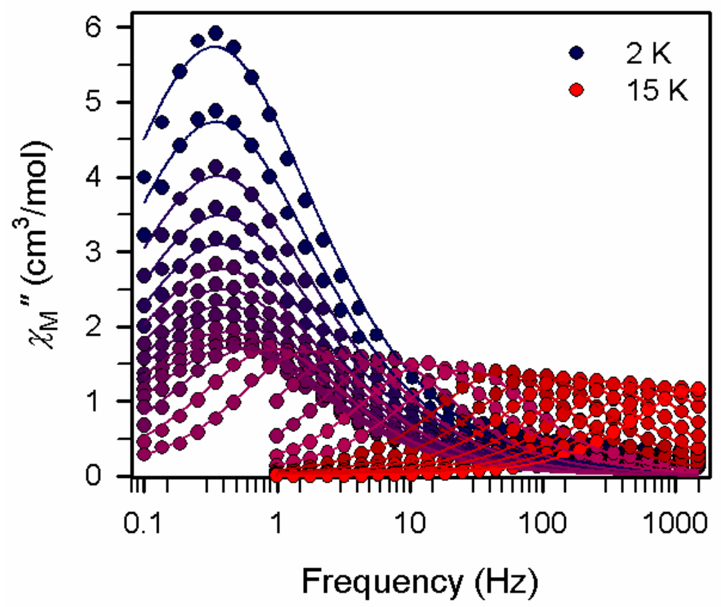

Supplementary Figure 29. Out-of-phase (χ_M'') components of the ac magnetic susceptibility for **1-Dy** under zero applied dc field from 2 K (dark blue circles) to 15 K (red circles). Solid lines represent a fit to the data.

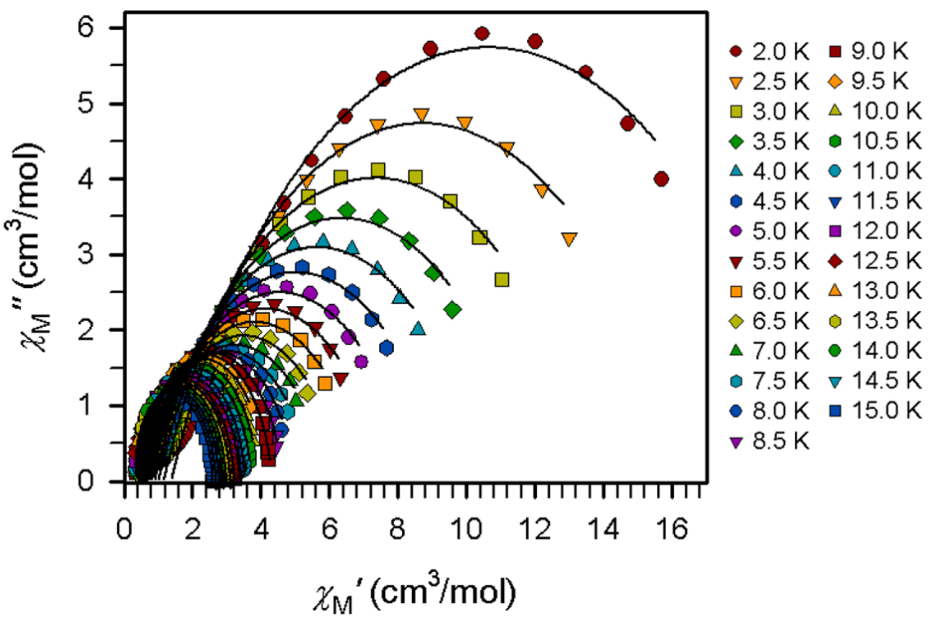

Supplementary Figure 30. Cole-Cole (Argand) plots for ac susceptibility collected from 2 to 15 K under zero applied dc field for **1-Dy**. Symbols represent the experimental data points and the points representing the fits are connected by solid black lines.

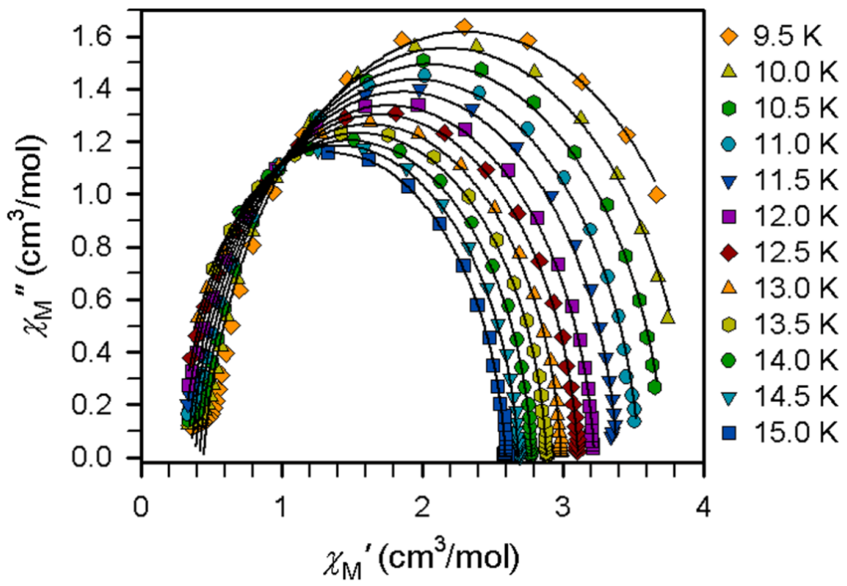

Supplementary Figure 31. Cole-Cole (Argand) plots for ac susceptibility collected under zero applied dc field for **1-Dy** shown for the temperature range 9.5 to 15 K. Symbols represent the experimental data points and the points representing the fits are connected by solid black lines.

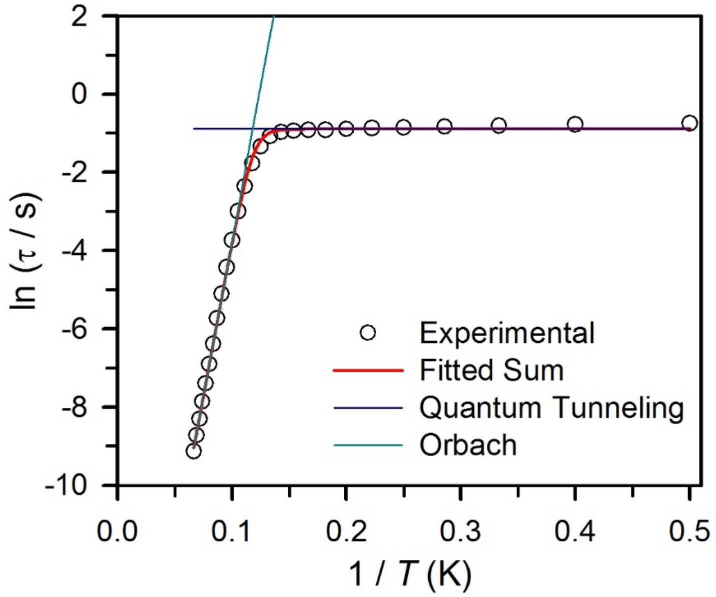

Supplementary Figure 32. Individual contributions of the multiple magnetic relaxation pathways to the Arrhenius plot of **1-Dy** at 0 Oe. Individual parameters used to calculate the contributions are given in Supplementary Table 1. The black line represents a fit to one Orbach relaxation processes and a tunneling pathway.

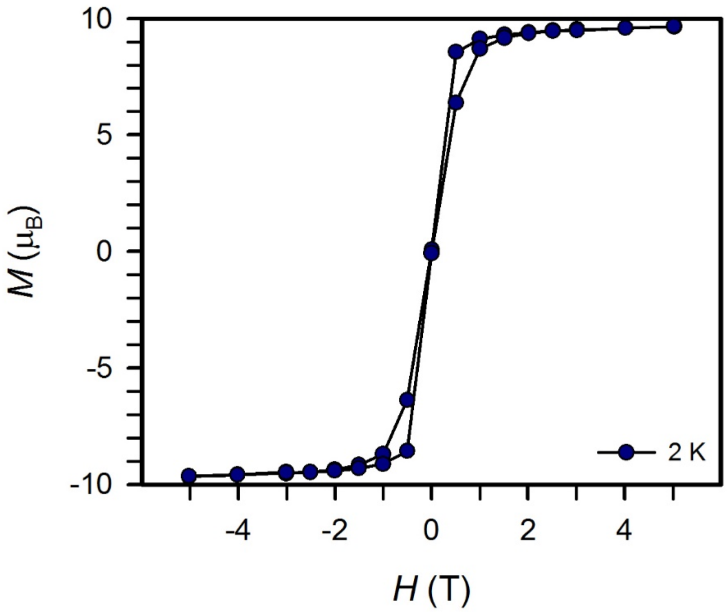

Supplementary Figure 33. Plot of magnetization (*M*) vs dc magnetic field (*H*) at an average sweep rate of 0.01 T/s for **1-Dy**.

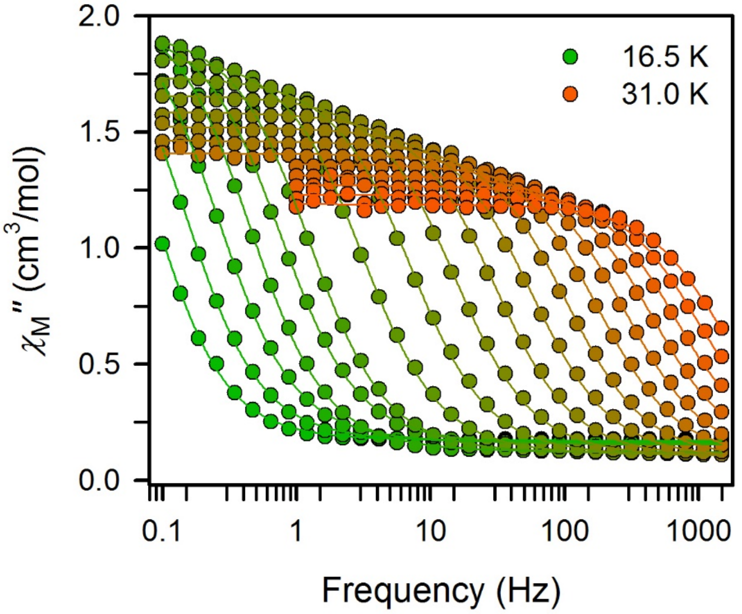

Supplementary Figure 34. In-phase (χ_M') components of the ac magnetic susceptibility for **1-Tb** under zero applied dc field from 16.5 K (green circles) to 31.0 K (orange circles). Solid lines represent a fit to the data.

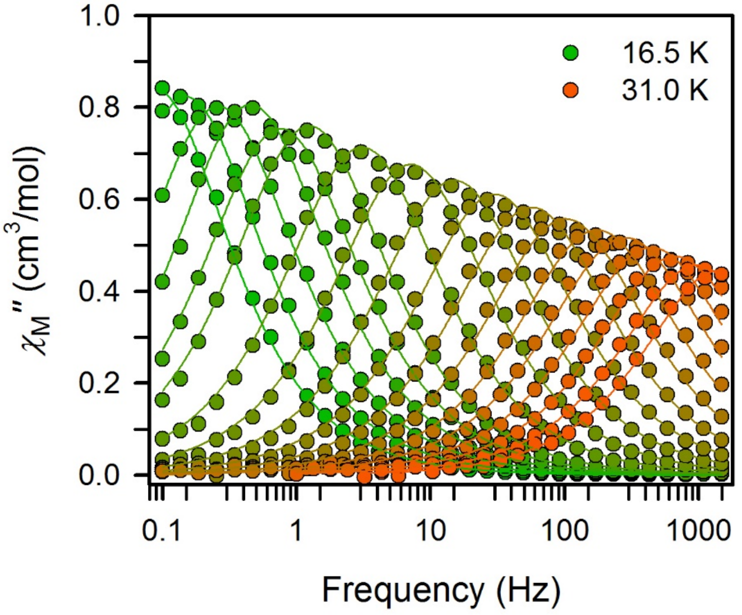

Supplementary Figure 35. Out-of-phase (χ_M'') components of the ac magnetic susceptibility for **1-Tb** under zero applied dc field from 16.5 K (green circles) to 31.0 K (orange circles). Solid lines represent a fit to the data.

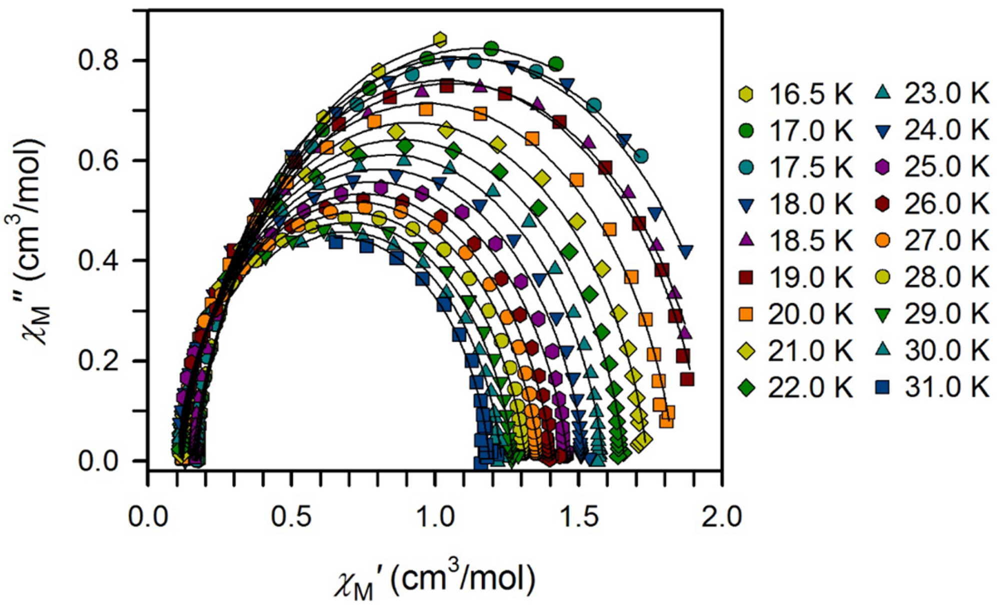

Supplementary Figure 36. Cole-Cole (Argand) plots for ac susceptibility collected under zero applied dc field for **1-Tb**. Symbols represent the experimental data points and the points representing the fits are connected by solid black lines.

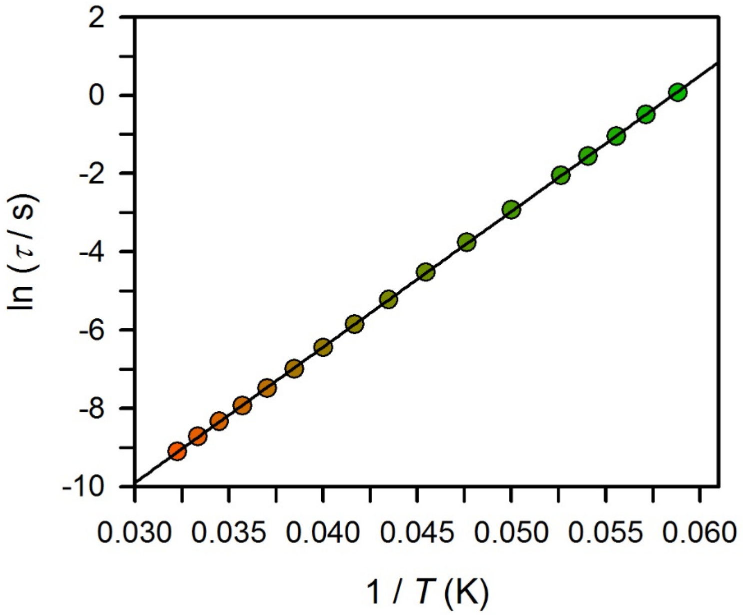

Supplementary Figure 37. Arrhenius plot of the natural log of the relaxation time, τ, versus the inverse temperature obtained from ac measurements for **1-Tb** (green to orange circles).

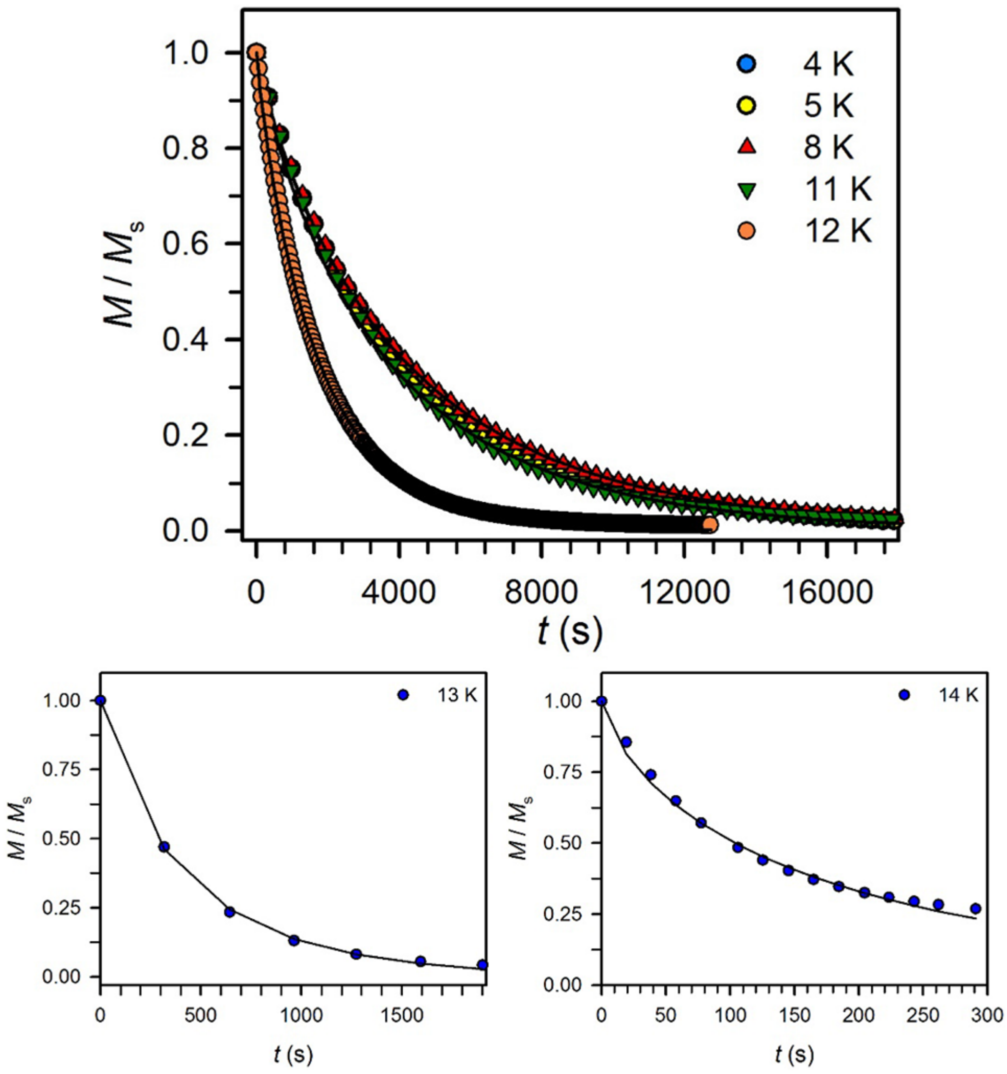

Supplementary Figure 38. Plots of magnetization vs. time used to derive relaxation times for **1-Tb** at different temperatures. The data were fit to a function of the form $y = a \cdot \exp(-((t/\tau)^b))$ where b is a stretch factor (black line).

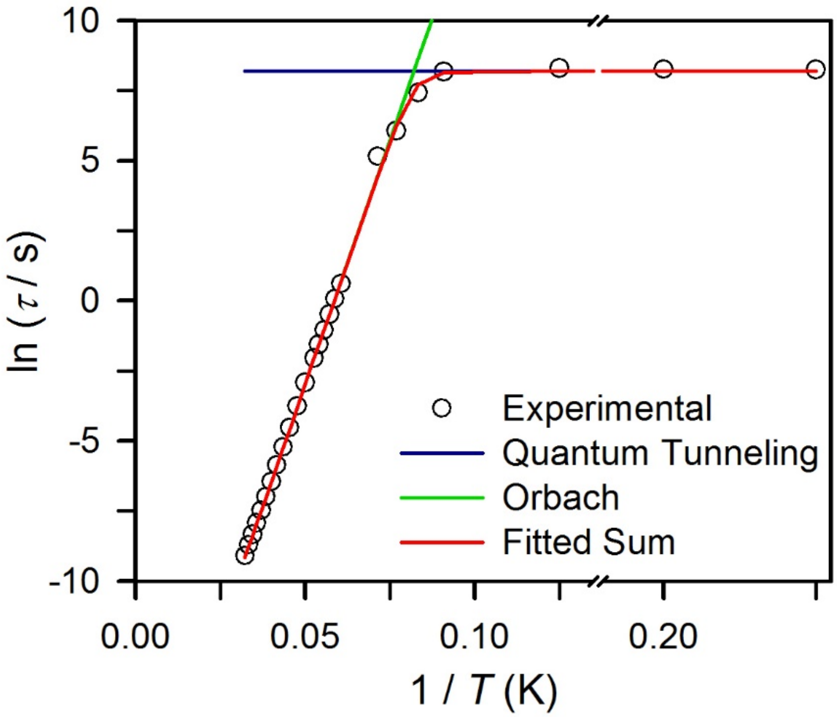

Supplementary Figure 39. Individual contributions of the multiple magnetic relaxation pathways to the Arrhenius plot of **1-Tb** at 0 Oe (see Figure 4). Individual parameters used to calculate the contributions are given in Supplementary Table 1. The red line represents a fit to one Orbach relaxation processes and a tunneling pathway.

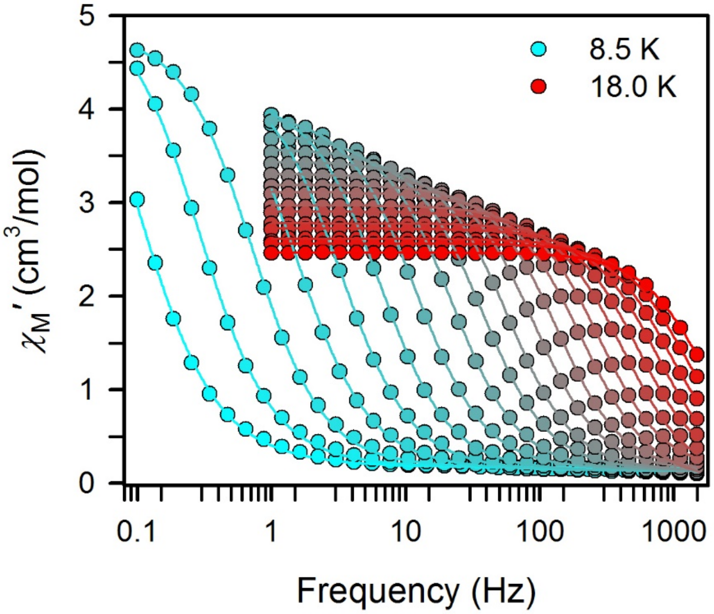

Supplementary Figure 40. In-phase (χ_M') components of the ac magnetic susceptibility for **2-Dy** under zero applied dc field from 8.5 K (cyan circles) to 18 K (red circles). Solid lines represent a fit to the data.

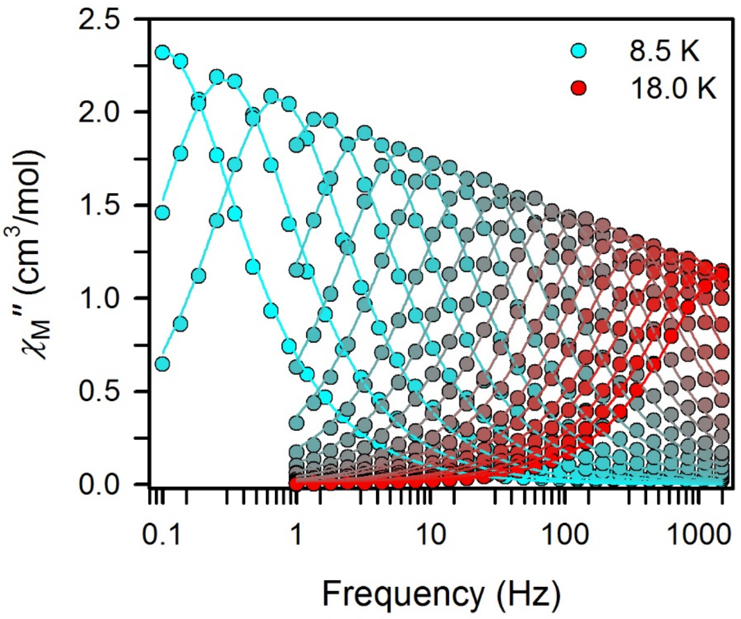

Supplementary Figure 41. Out-of-phase (χ_M'') components of the ac magnetic susceptibility for **2-Dy** under zero applied dc field from 8.5 K (cyan circles) to 18 K (red circles). Solid lines represent a fit to the data.

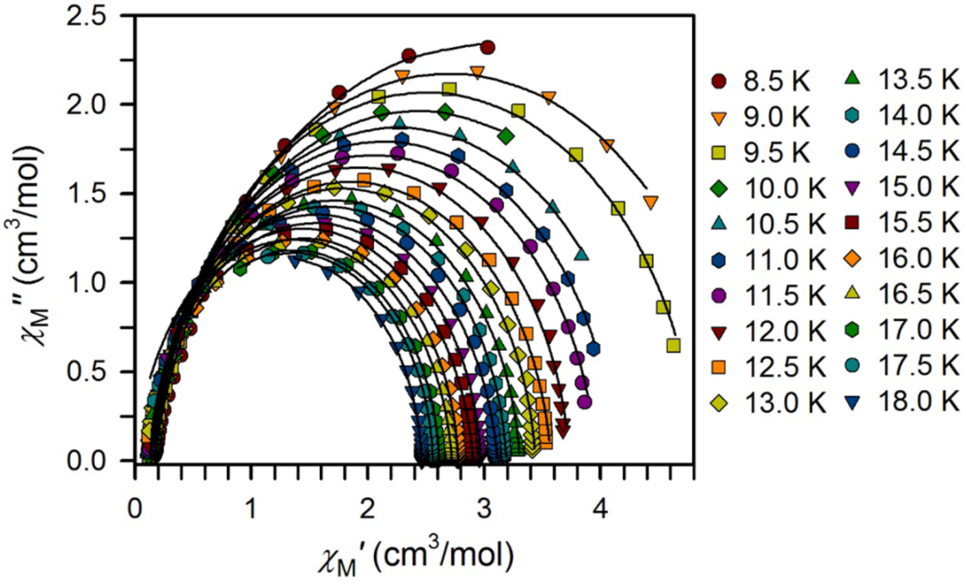

Supplementary Figure 42. Cole-Cole (Argand) plots for ac susceptibility collected from 8.5 to 18 K under zero applied dc field for **2-Dy**. Symbols represent the experimental data points and the points representing the fits are connected by solid black lines.

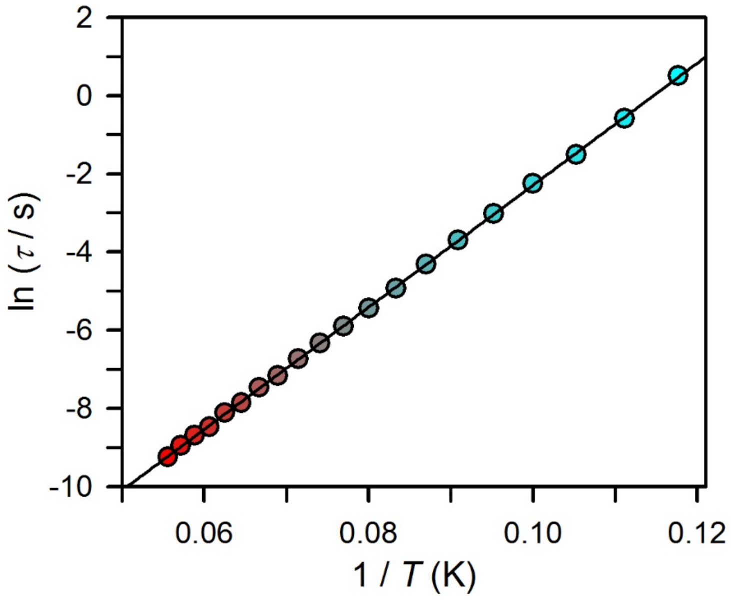

Supplementary Figure 43. Arrhenius plot of the natural log of the relaxation time, τ, versus the inverse temperature obtained from ac measurements, for **2-Dy** (cyan to red circles).

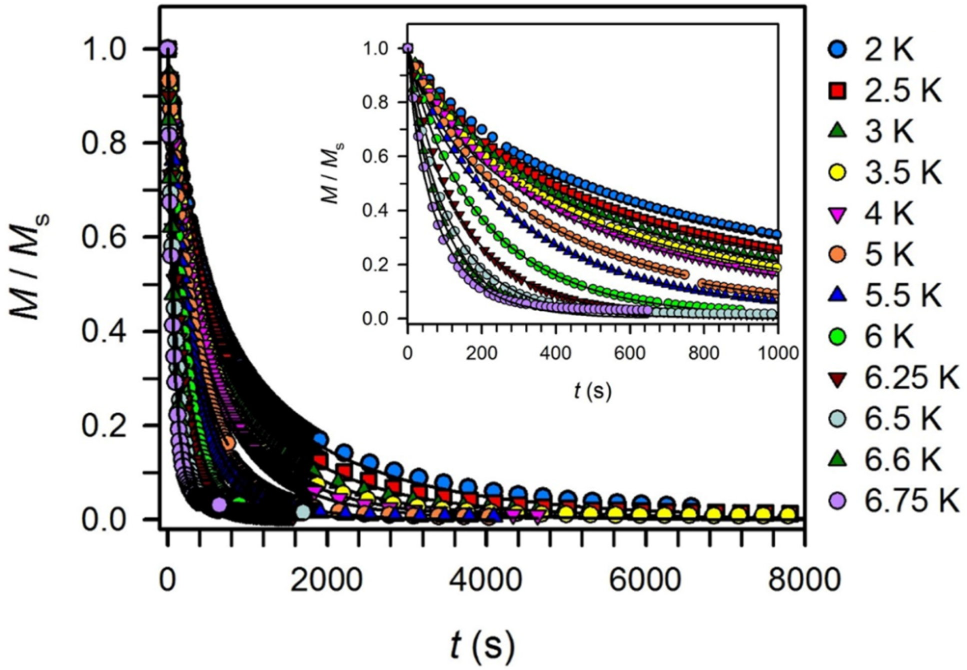

Supplementary Figure 44. Plots of magnetization vs. time used to derive relaxation times for **2-Dy** at different temperatures. The data were fit to a function of the form $y = a \cdot \exp(-((t/\tau)^b))$ where b is a stretch factor (black line). Inset: Data is shown until t = 1000 s for clarity. Decay of the magnetization versus time for **2-Dy**, obtained by applying a magnetic field of 7 T to the sample at a temperature of 60 K, cooling the sample to a given temperature, and then removing the magnetic field.

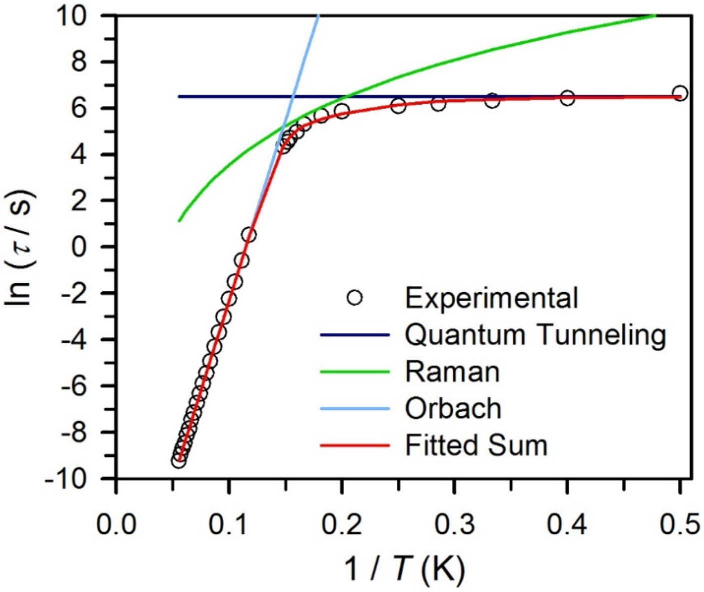

Supplementary Figure 45. Individual contributions of the multiple magnetic relaxation pathways to the Arrhenius plot of **2-Dy** at 0 Oe (see Figure 4). Individual parameters used to calculate the contributions are given in Supplementary Table 1. The red line represents a fit to one Orbach relaxation processes, Raman process and a tunneling pathway.

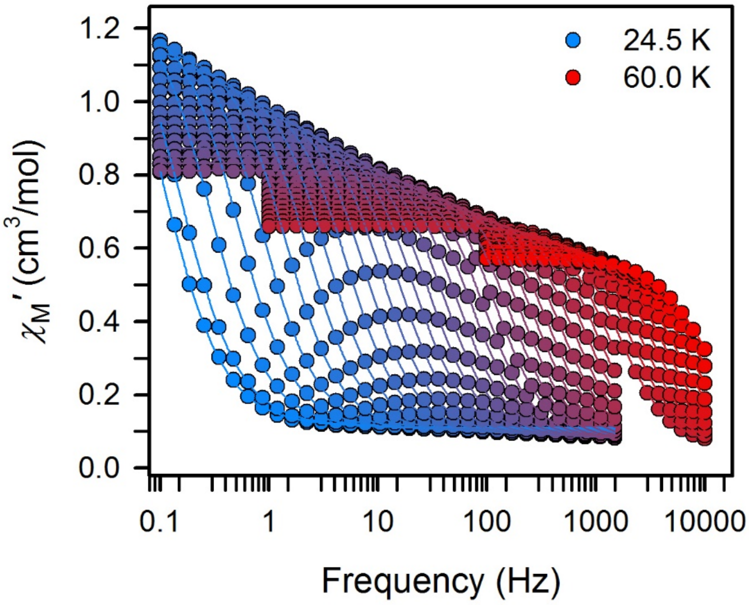

Supplementary Figure 46. In-phase (χ_M') components of the ac magnetic susceptibility for **2-Tb** under zero applied dc field from 24.5 K (blue circles) to 60 K (red circles). Solid lines represent a fit to the data.

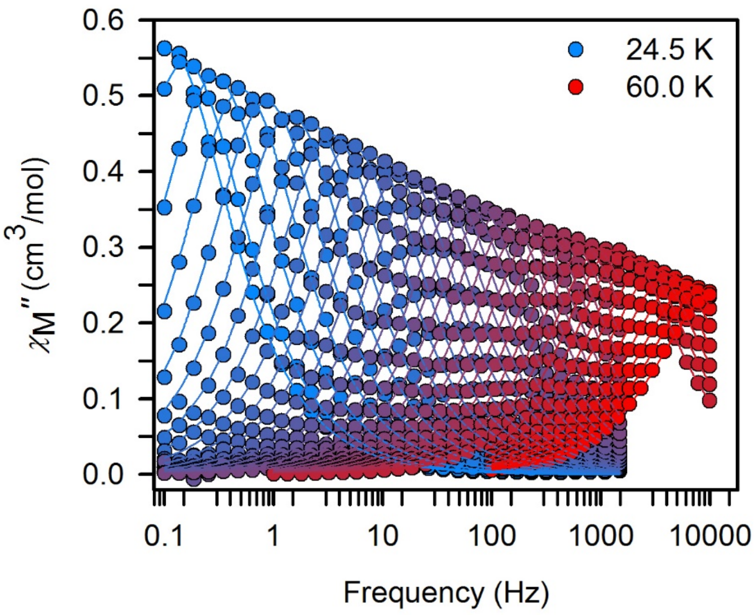

Supplementary Figure 47. Out-of-phase (χ_M'') components of the ac magnetic susceptibility for **2-Tb** under zero applied dc field from 24.5 K (blue circles) to 60 K (red circles). Solid lines represent a fit to the data.

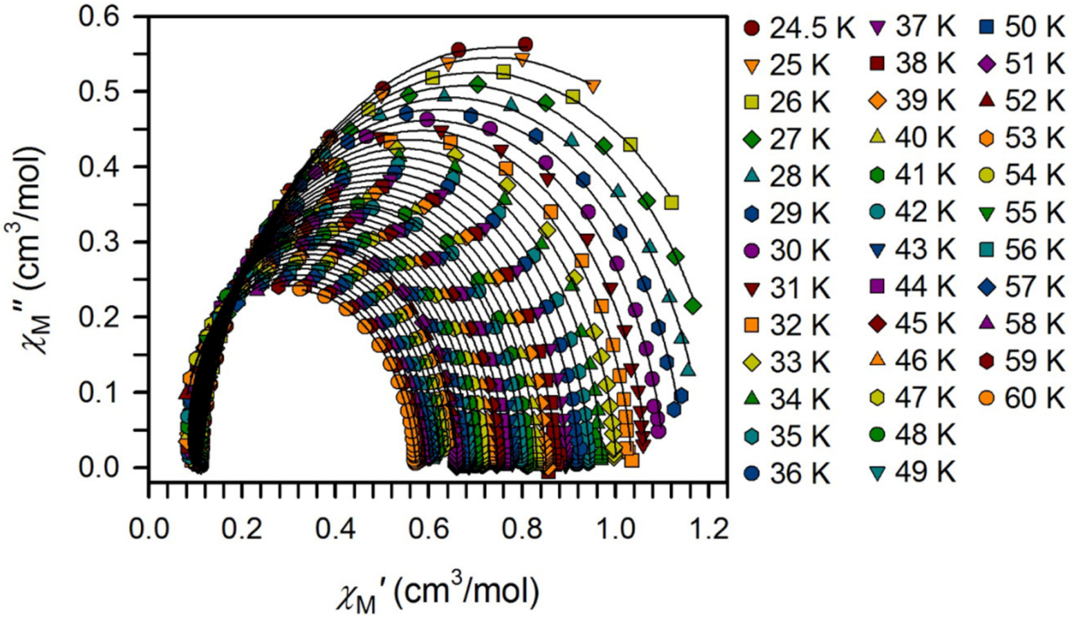

Supplementary Figure 48. Cole-Cole (Argand) plots for ac susceptibility collected from 24.5 to 60 K under zero applied dc field for **2-Tb**. Symbols represent the experimental data points and the points representing the fits are connected by solid black lines.

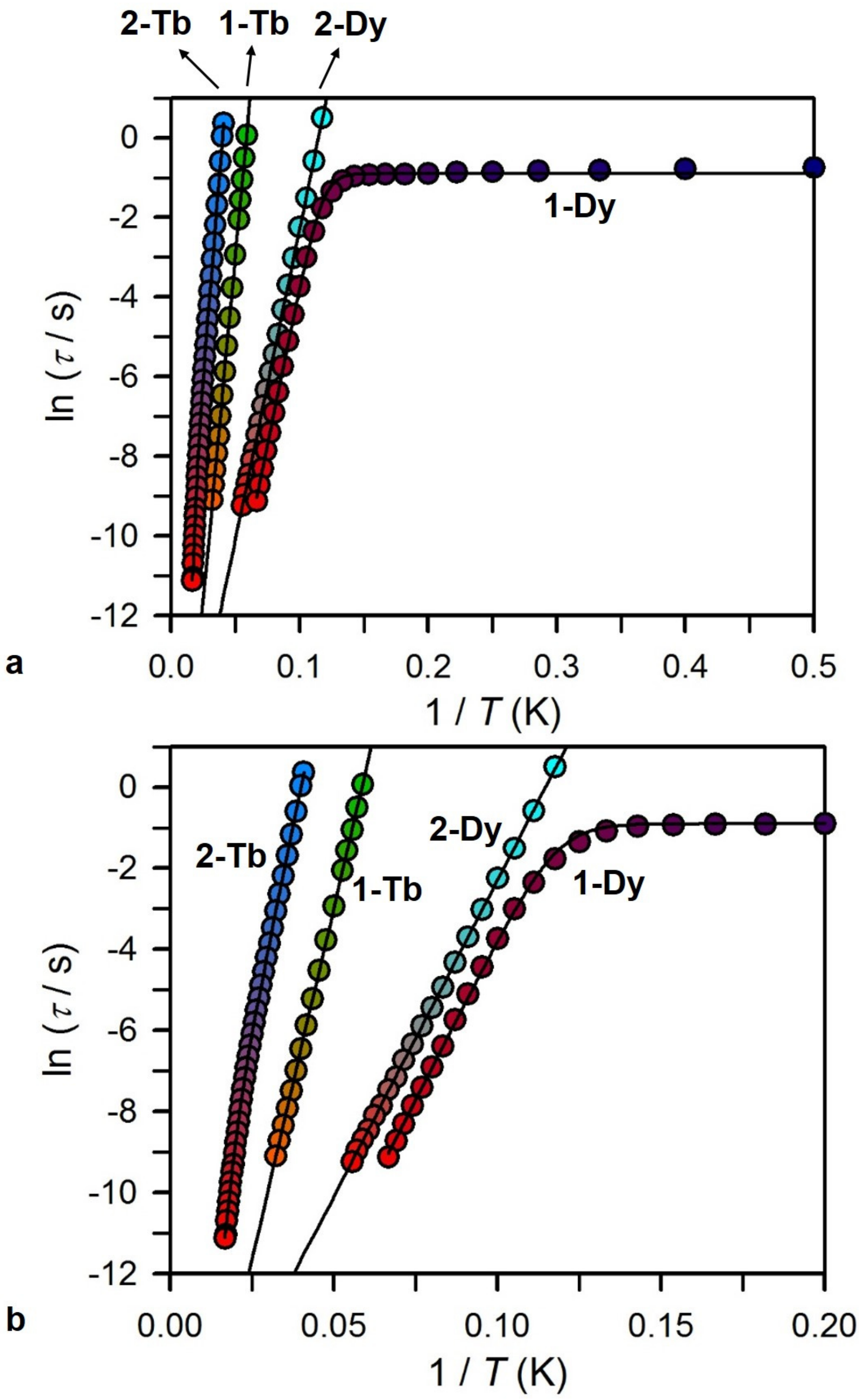

Supplementary Figure 49. (**a**, **b**) Arrhenius plot of the natural log of the relaxation time, τ, versus the inverse temperature obtained from ac measurements, for **1-Tb** (green to red circles), **1-Dy** (dark blue to red circles), **2-Tb** (blue to red circles) and **2-Dy** (cyan to red circles). (b) Arrhenius plot for **1-Dy** is depicted only for temperatures from 5 to 15 K; the τ below 5 K are omitted for clarity.

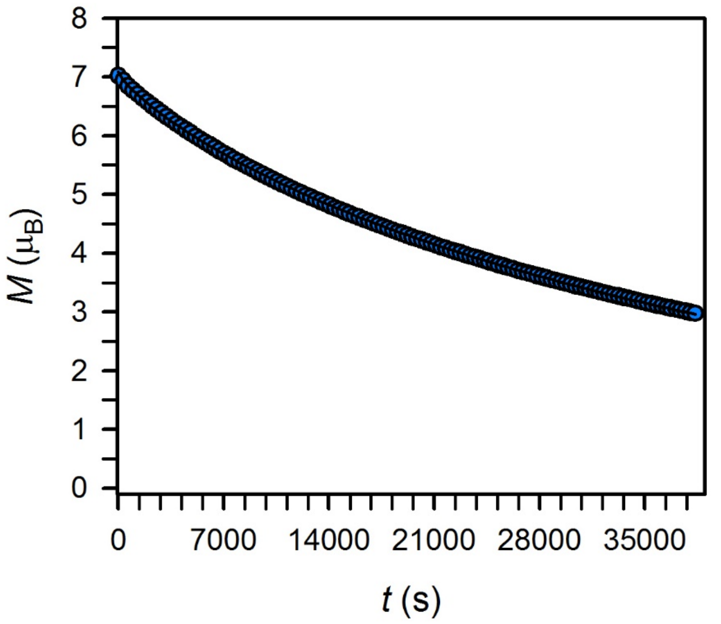

Supplementary Figure 50. Plot of magnetization vs. time used to derive relaxation times for **2-Tb** at 2 K. The data (pale blue circles) were fit to a function of the form $y = a \cdot \exp(-((t/\tau)^b))$ where b is a stretch factor (black line). Decay of the magnetization vs. time for **2-Tb**, obtained by applying a magnetic field of 7 T to the sample at a temperature of 80 K, cooling the sample to 2 K, and then removing the magnetic field.

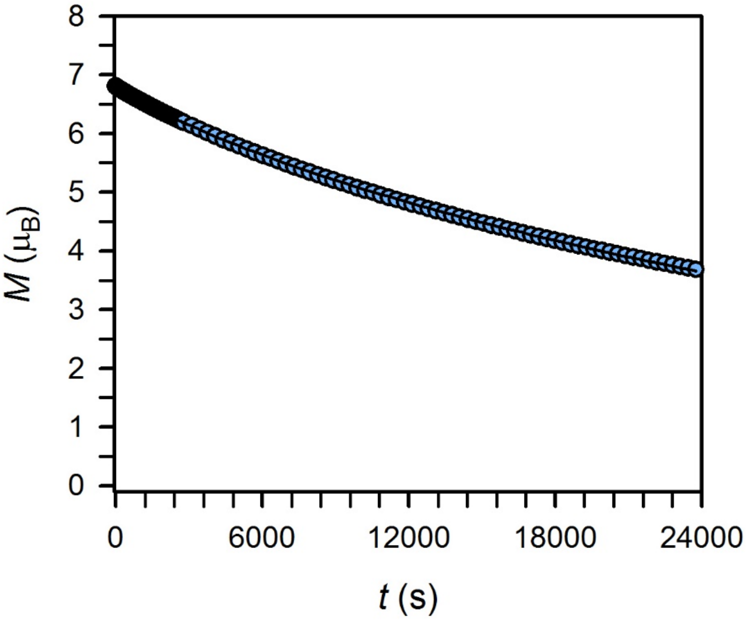

Supplementary Figure 51. Plot of magnetization vs. time used to derive relaxation times for **2-Tb** at 11 K. The data (pale blue circles) were fit to a function of the form $y = a \cdot \exp(-((t/\tau)^b))$ where b is a stretch factor (black line). Decay of the magnetization vs. time for **2-Tb**, obtained by applying a magnetic field of 7 T to the sample at a temperature of 80 K, cooling the sample to 11 K, and then removing the magnetic field.

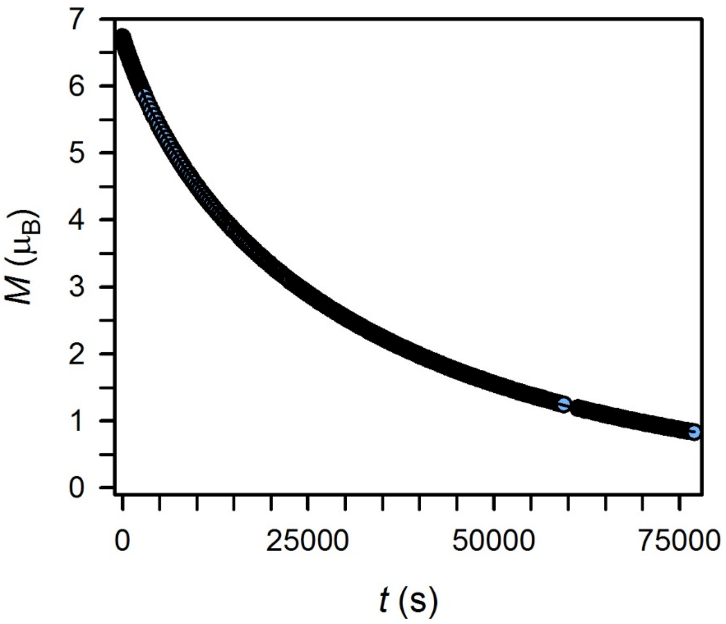

Supplementary Figure 52. Plot of magnetization vs. time used to derive relaxation times for **2-Tb** at 14 K. The data (pale blue circles) were fit to a function of the form $y = a \cdot \exp(-((t/\tau)^b))$ where b is a stretch factor (black line). Decay of the magnetization vs. time for **2-Tb**, obtained by applying a magnetic field of 7 T to the sample at a temperature of 80 K, cooling the sample to 14 K, and then removing the magnetic field.

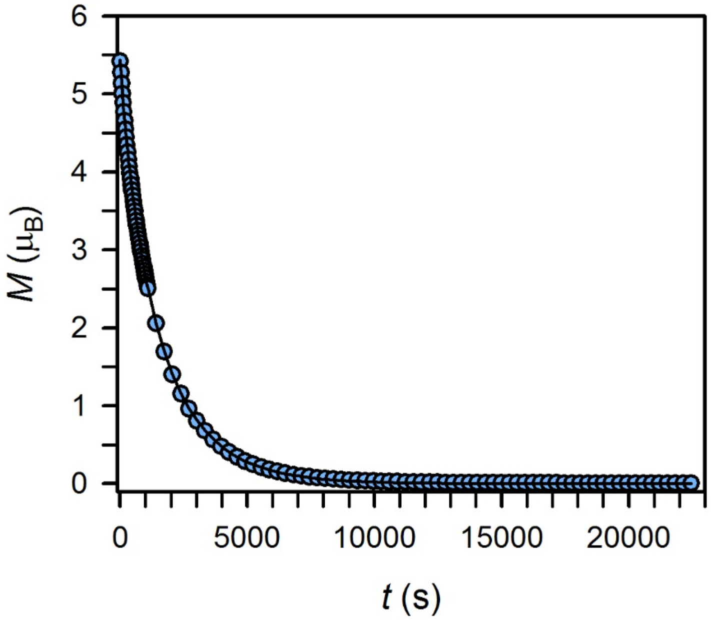

Supplementary Figure 53. Plot of magnetization vs. time used to derive relaxation times for **2-Tb** at 17 K. The data (pale blue circles) were fit to a function of the form $y = a \cdot \exp(-((t/\tau)^b))$ where b is a stretch factor (black line). Decay of the magnetization vs. time for **2-Tb**, obtained by applying a magnetic field of 7 T to the sample at a temperature of 80 K, cooling the sample to 17 K, and then removing the magnetic field.

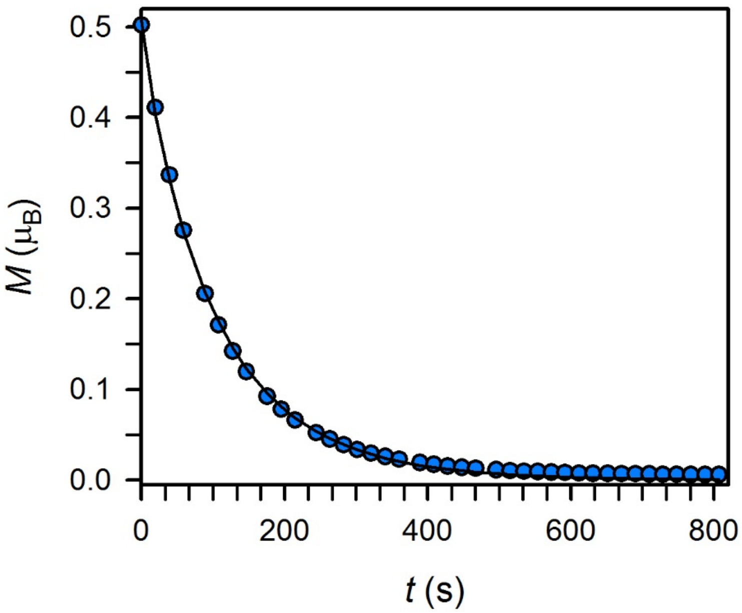

Supplementary Figure 54. Plot of magnetization vs. time used to derive relaxation times for **2-Tb** at 19.55 K. The data (pale blue circles) were fit to a function of the form $y = a \cdot \exp(-((t/\tau)^b))$ where b is a stretch factor (black line). Decay of the magnetization vs. time for **2-Tb**, obtained by applying a magnetic field of 7 T to the sample at a temperature of 80 K, cooling the sample to 19.55 K, and then removing the magnetic field.

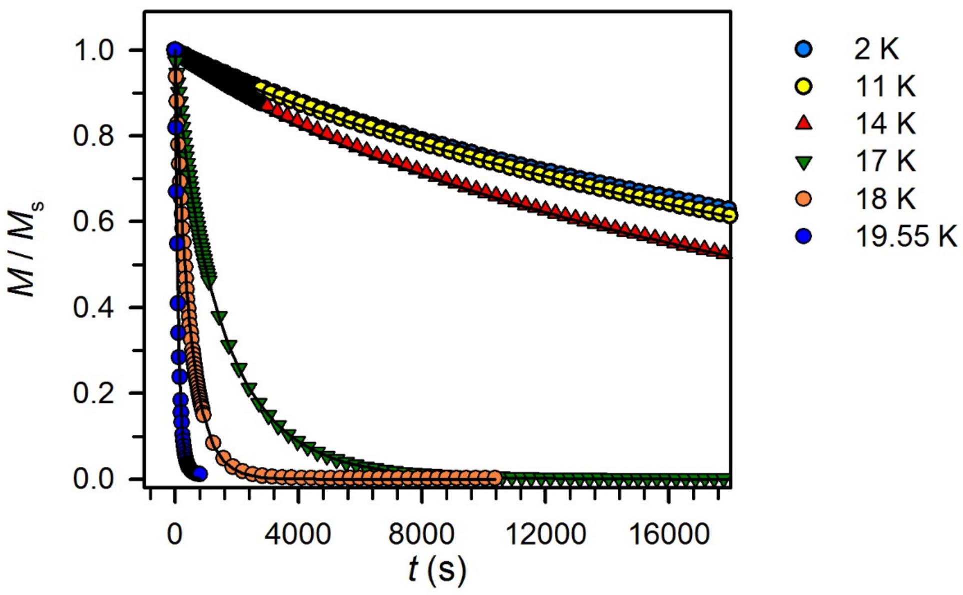

Supplementary Figure 55. Plot of magnetization (normalized) vs. time used to derive relaxation times for **2-Tb** at different temperatures. The data were fit to a function of the form y = a · exp $(-((t/\tau)^b))$ where b is a stretch factor (black line). Decay of the magnetization vs. time for **2-Tb**, obtained by applying a magnetic field of 7 T to the sample at a temperature of 80 K, cooling the sample to a given temperature, and then removing the magnetic field.

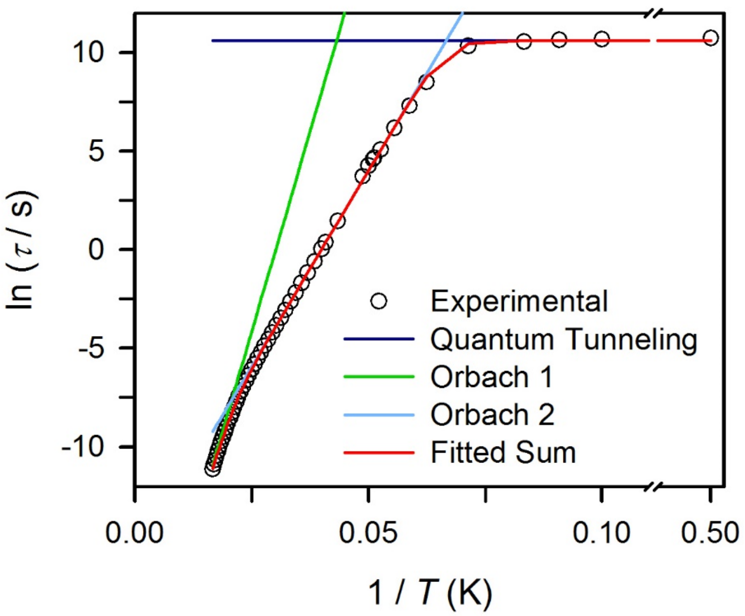

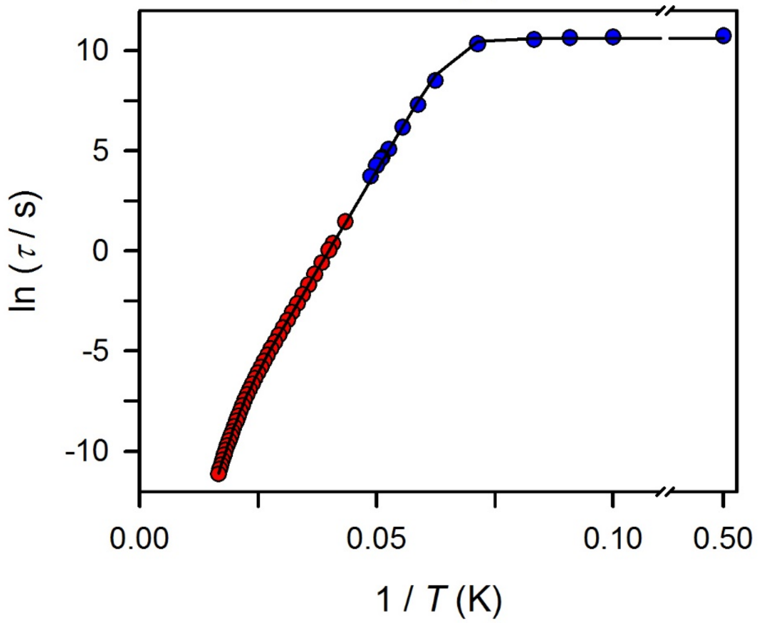

Supplementary Figure 56. (top) Individual contributions of the multiple magnetic relaxation pathways to the Arrhenius plot of **2-Tb** at 0 Oe. Individual parameters used to calculate the contributions are given in Supplementary Table 1. (bottom) Plot of natural log of the relaxation time versus the inverse temperature (temperature range 2 to 60 K) for **2-Tb**. Red and blue circles represent data extracted from ac and dc susceptibility measurements, respectively. The black line represents a fit to two Orbach relaxation processes and a tunneling pathway as described in Figure 4.

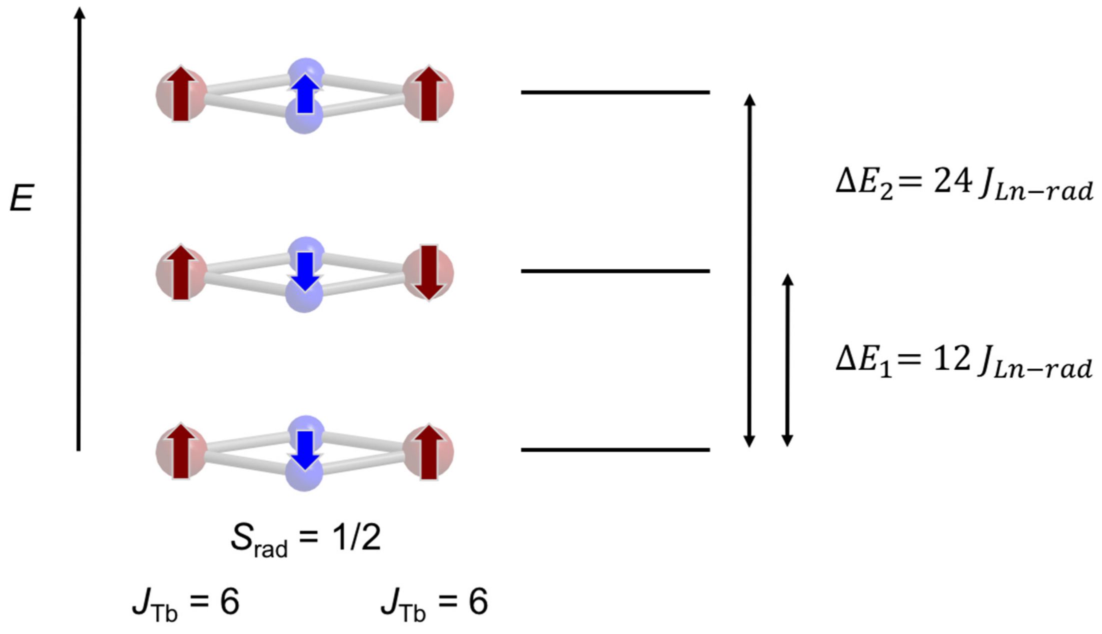

Supplementary Figure 57. Proposed energy level splitting diagram for a $\{Tb_2(\mu\text{-}N_2^{\cdot})\}$ moiety, highlighting the equal spacing between energy levels, the magnitude of which is dependent on multiples of $2J_{\text{Ln-rad}}$ (assuming use of a $2J$ Hamiltonian), as described in Supplementary Methods Section 4.

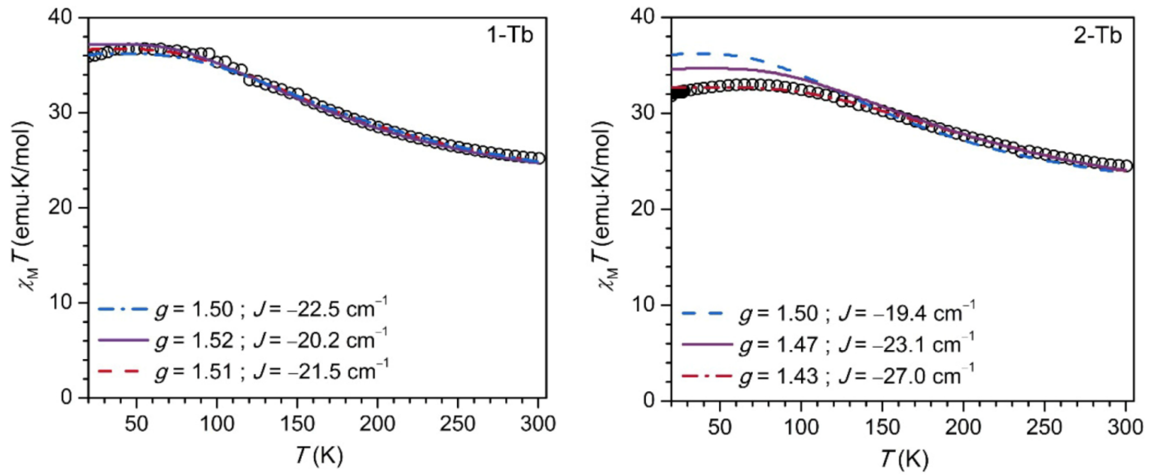

Supplementary Figure 58. Models of $\chi_M T$ data of polycrystalline **1-Tb** and **2-Tb** collected under 0.1 T applied dc field (see Supplementary Methods Section 4 and Supplementary Table 5 for model details).

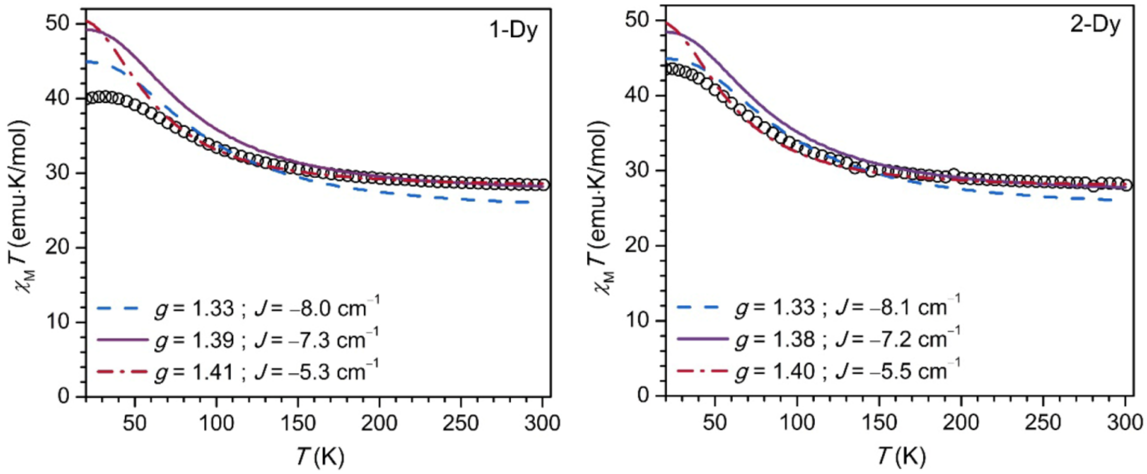

Supplementary Figure 59. Models of $\chi_M T$ data of polycrystalline **1-Dy** and **2-Dy** collected under 0.1 T applied dc field (see Supplementary Methods Section 4 and Supplementary Table 6 for model details).

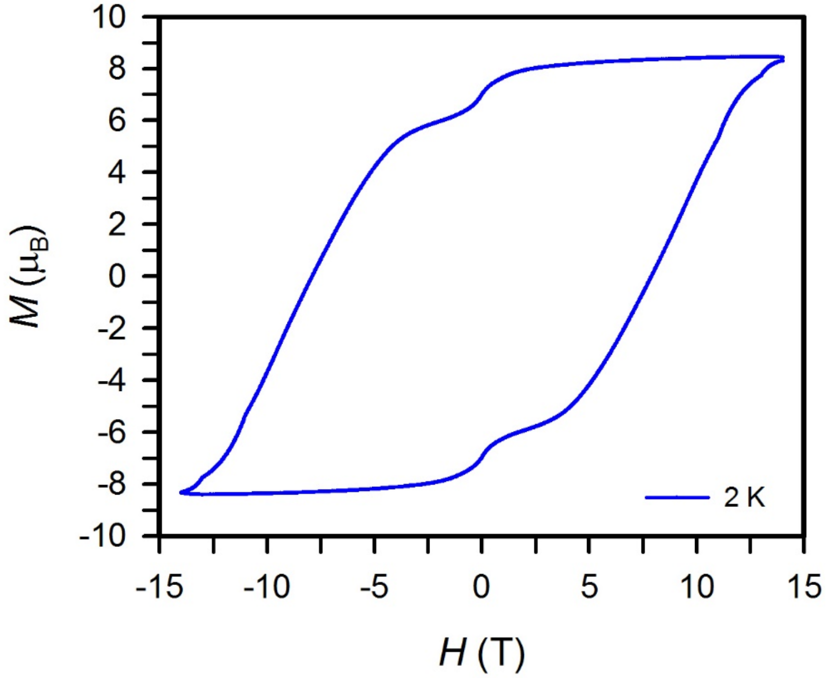

Supplementary Figure 60. Plot of magnetization (*M*) vs dc magnetic field (*H*) at an average sweep rate of 0.01 T/s for **2-Tb** at 2 K.

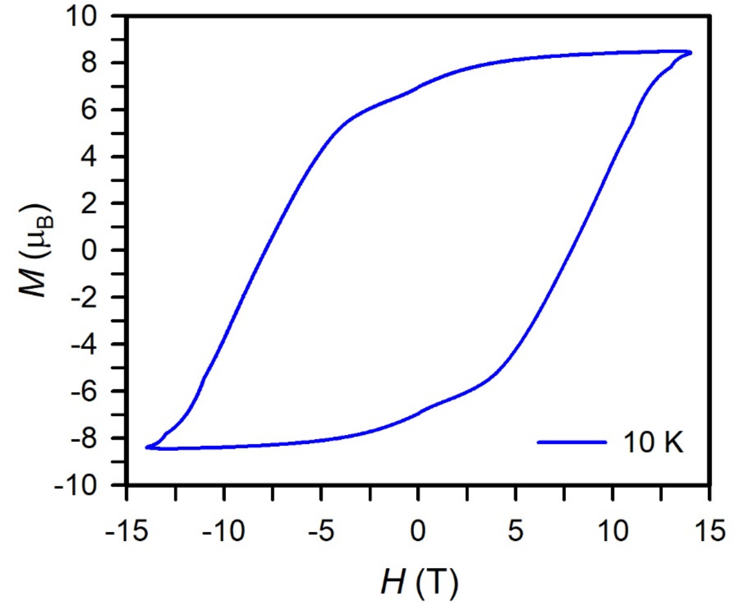

Supplementary Figure 61. Plot of magnetization (*M*) vs dc magnetic field (*H*) at an average sweep rate of 0.01 T/s for **2-Tb** at 10 K.

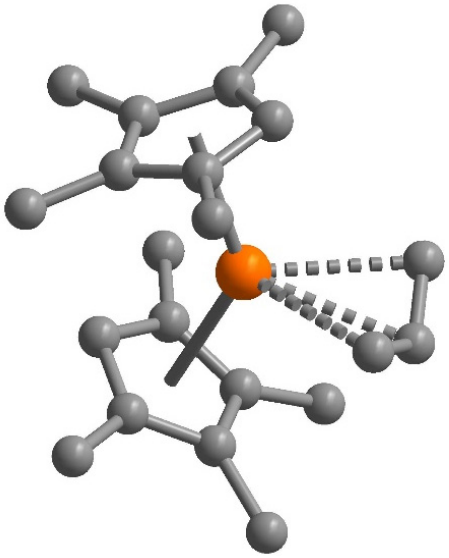

Supplementary Figure 62. Structure of $Cp^{Me4H}{}_2Gd(\eta^3\text{-}C_3H_5)$. Orange and gray spheres represent Gd and C atoms, respectively; H atoms have been omitted for clarity.

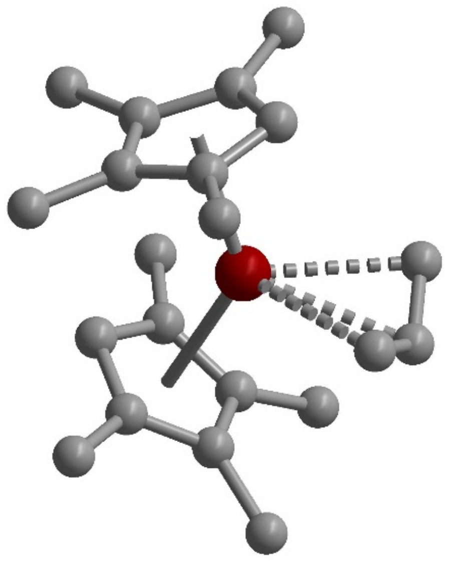

Supplementary Figure 63. Structure of $Cp^{Me4H}{}_2Tb(\eta^3\text{-}C_3H_5)$. Dark red and gray spheres represent Tb and C atoms, respectively; H atoms have been omitted for clarity.

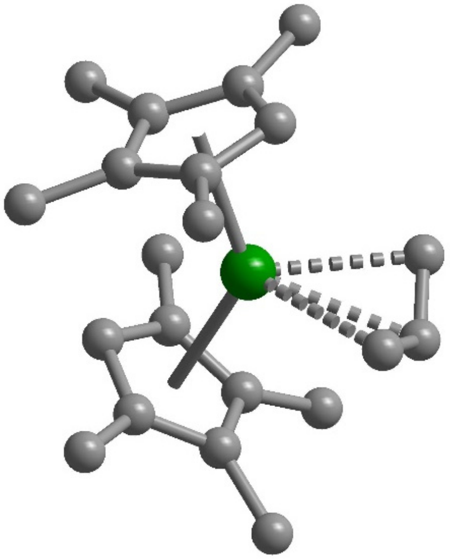

Supplementary Figure 64. Structure of $Cp^{Me4H}{}_2Dy(\eta^3\text{-}C_3H_5)$. Green and gray spheres represent Dy and C atoms, respectively; H atoms have been omitted for clarity.

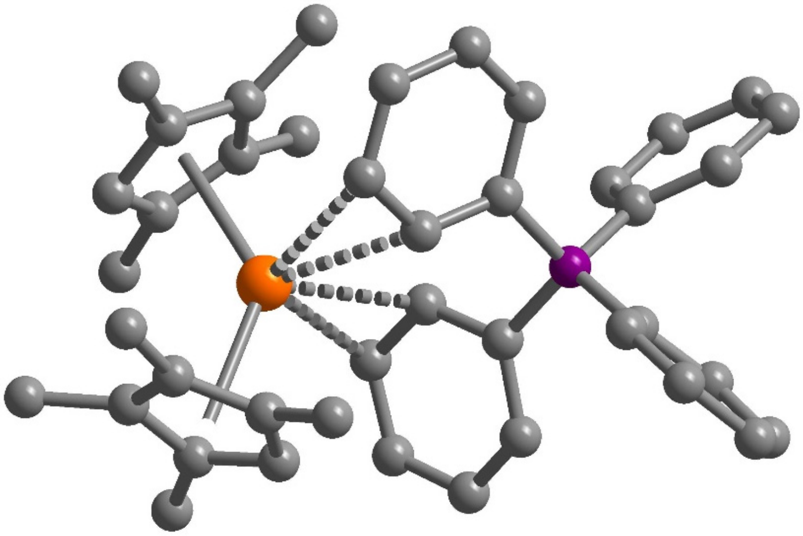

Supplementary Figure 65. Structure of $Cp^{tet}{}_2Gd(BPh_4)$. Orange, purple and gray spheres represent Gd, B and C atoms, respectively; H atoms have been omitted for clarity. The agostic interactions between the loosely ligated $(BPh_4)^{1-}$ anion and the lanthanide atom are indicated by dotted lines.

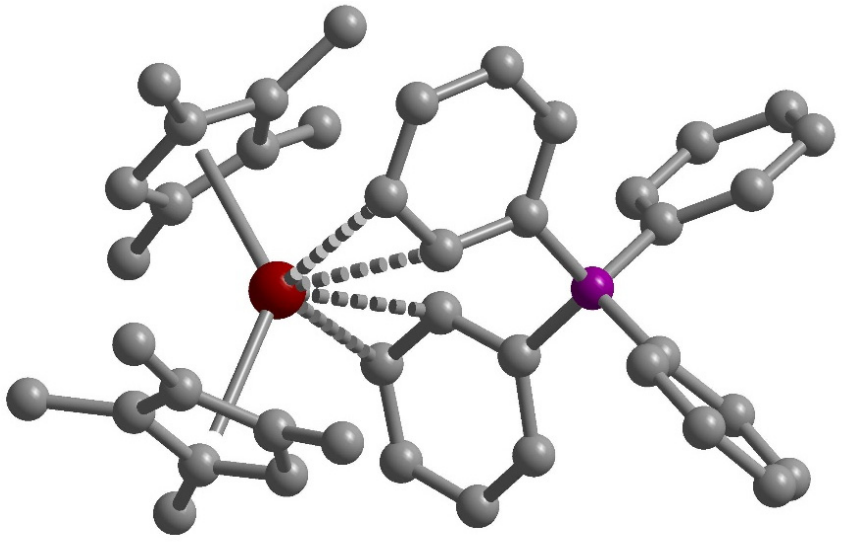

Supplementary Figure 66. Structure of $Cp^{tet}{}_2Tb(BPh_4)$. Dark red, purple and gray spheres represent Tb, B and C atoms, respectively; H atoms have been omitted for clarity. The agostic interactions between the loosely ligated $(BPh_4)^{1-}$ anion and the lanthanide atom are indicated by dotted lines.

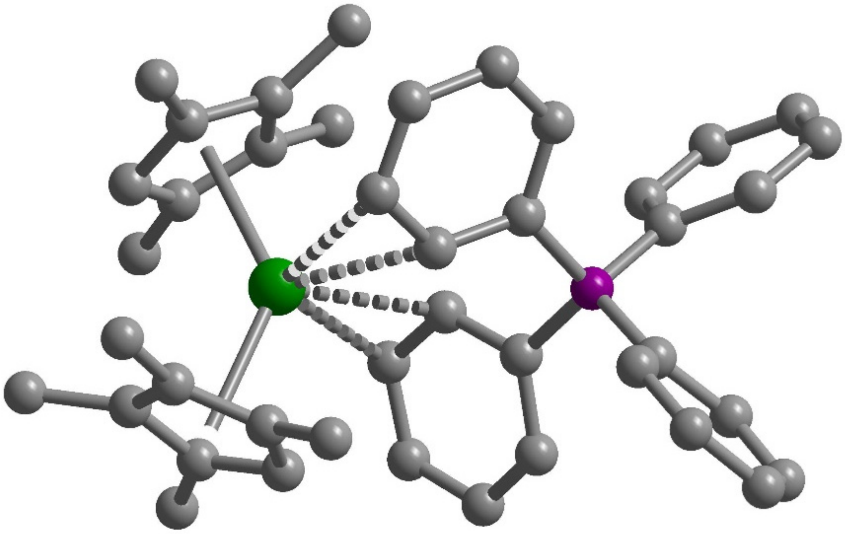

Supplementary Figure 67. Structure of $Cp^{tet}_2Dy(BPh_4)$. Green, purple and gray spheres represent Dy, B and C atoms, respectively; H atoms have been omitted for clarity. The agostic interactions between the loosely ligated $(BPh_4)^{1-}$ anion and the lanthanide atom are indicated by dotted lines.

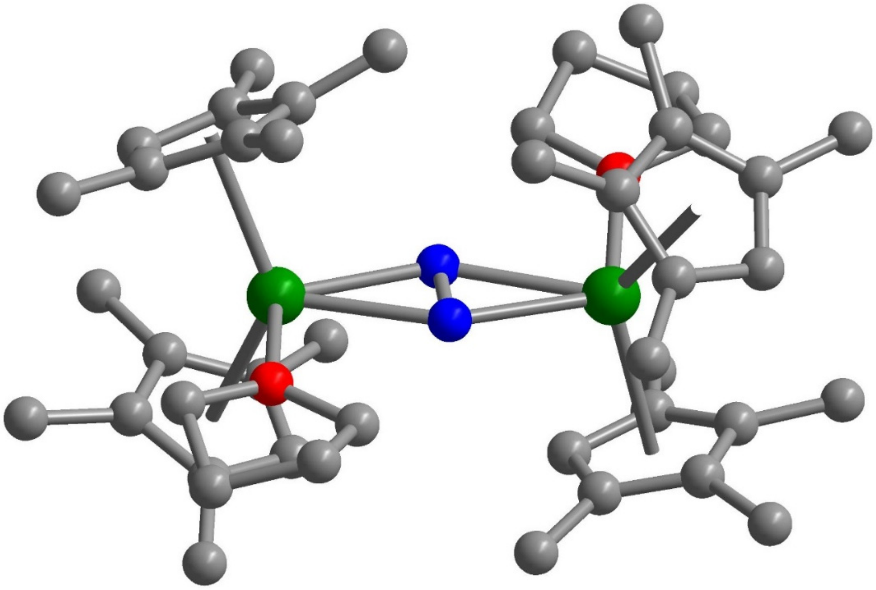

Supplementary Figure 68. Structure of the N_2^{2-} bridged complex in **3-Dy**. Orange, red, blue, and gray spheres represent Tb, O, N, and C atoms, respectively; H atoms have been omitted for clarity. The structure of **3-Dy** is similar to the reported compound in reference 17 but crystallized in a different cell.

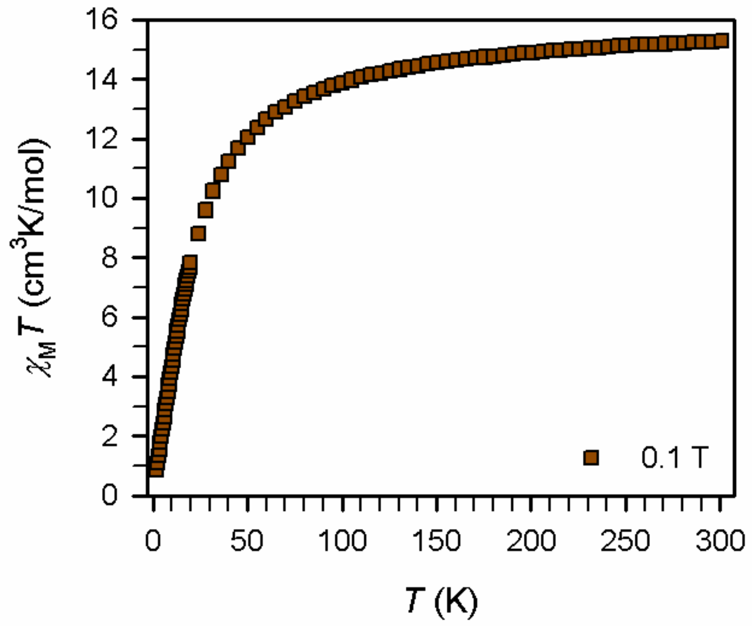

Supplementary Figure 69. Variable temperature dc susceptibility data of polycrystalline **3-Gd** collected under 0.1 T applied dc field.

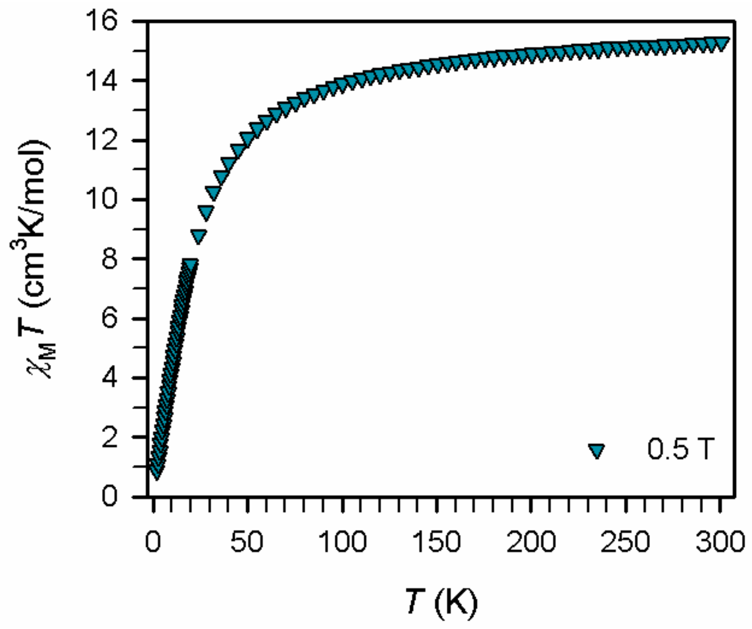

Supplementary Figure 70. Variable temperature dc susceptibility data of polycrystalline **3-Gd** collected under 0.5 T applied dc field.

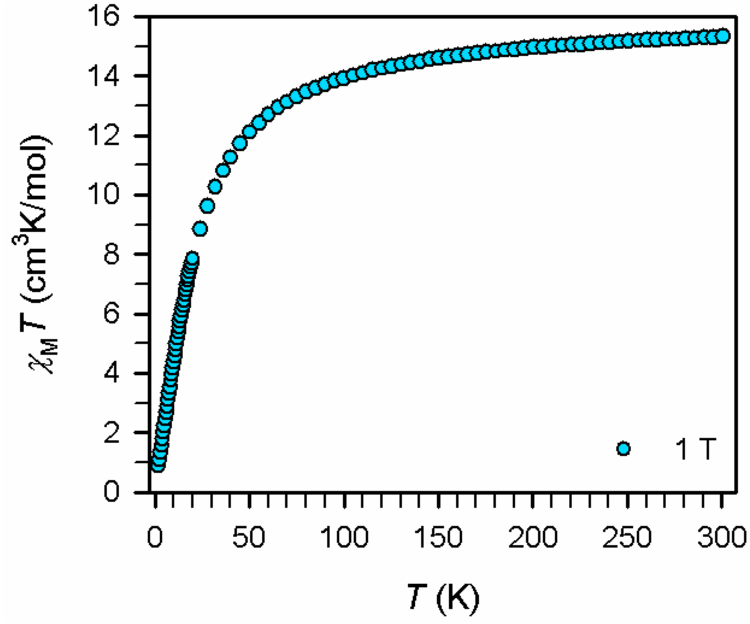

Supplementary Figure 71. Variable temperature dc susceptibility data of polycrystalline **3-Gd** collected under 1 T applied dc field.

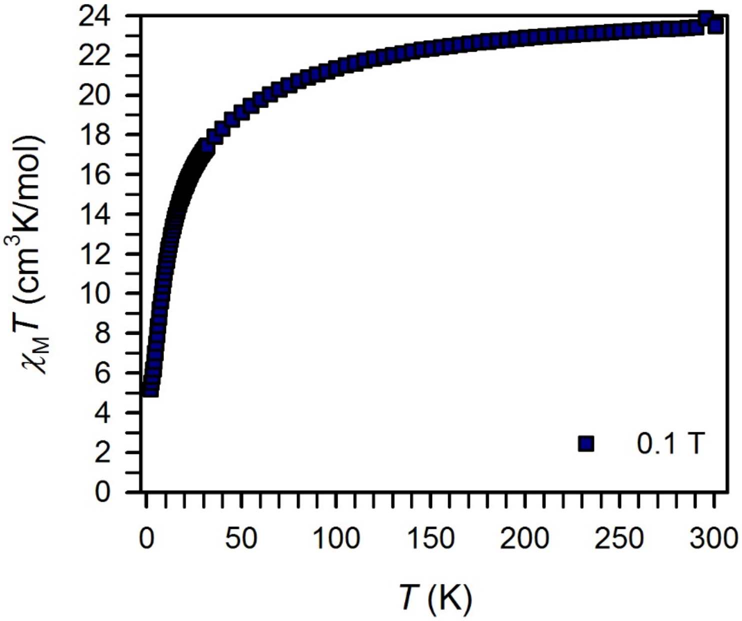

Supplementary Figure 72. Variable temperature dc susceptibility data of polycrystalline **3-Tb** collected under 0.1 T applied dc field.

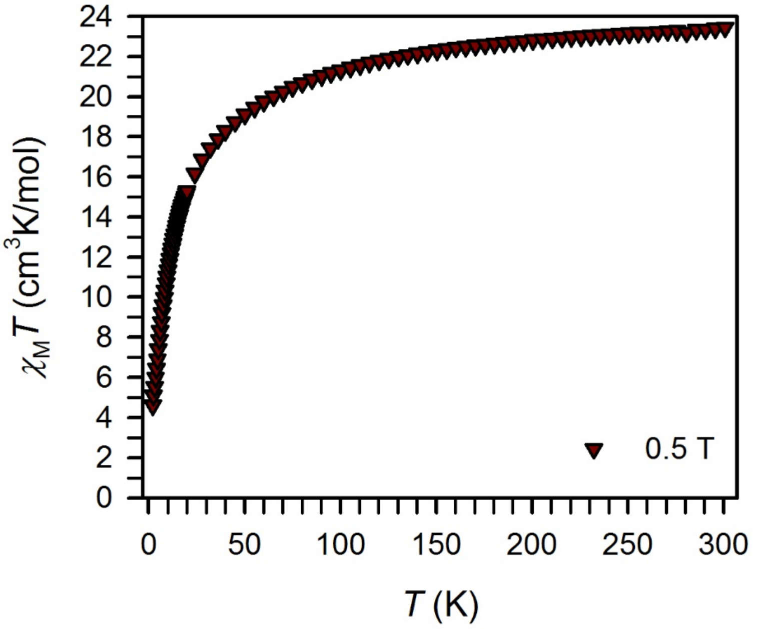

Supplementary Figure 73. Variable temperature dc susceptibility data of polycrystalline **3-Tb** collected under 0.5 T applied dc field.

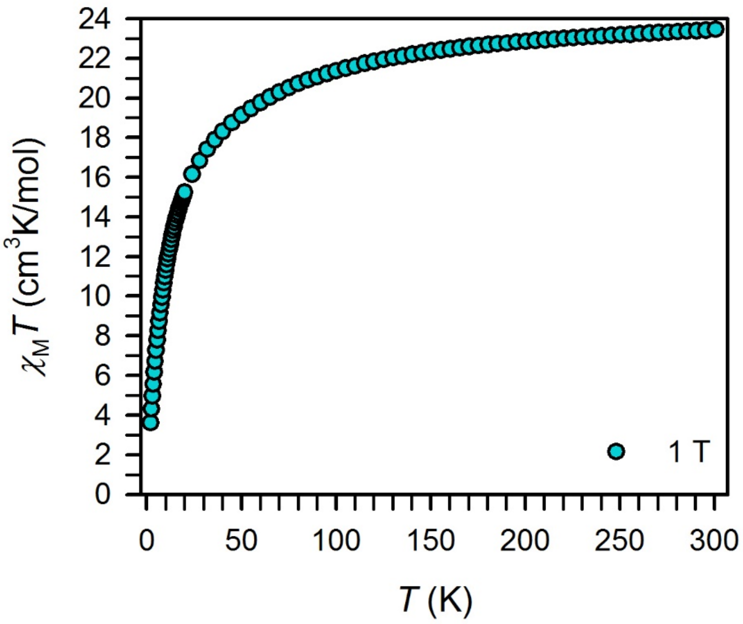

Supplementary Figure 74. Variable temperature dc susceptibility data of polycrystalline **3-Tb** collected under 1 T applied dc field.

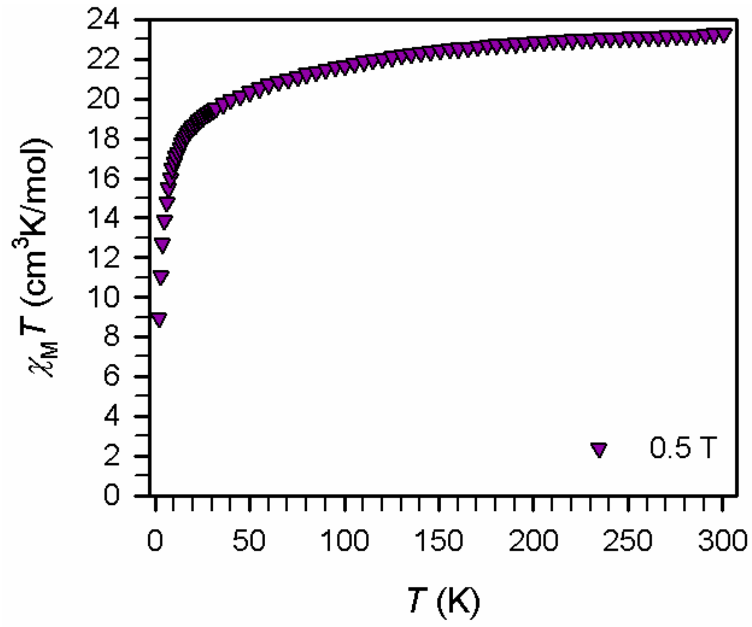

Supplementary Figure 75. Variable temperature dc susceptibility data of polycrystalline **3-Dy** collected under 0.5 T applied dc field.

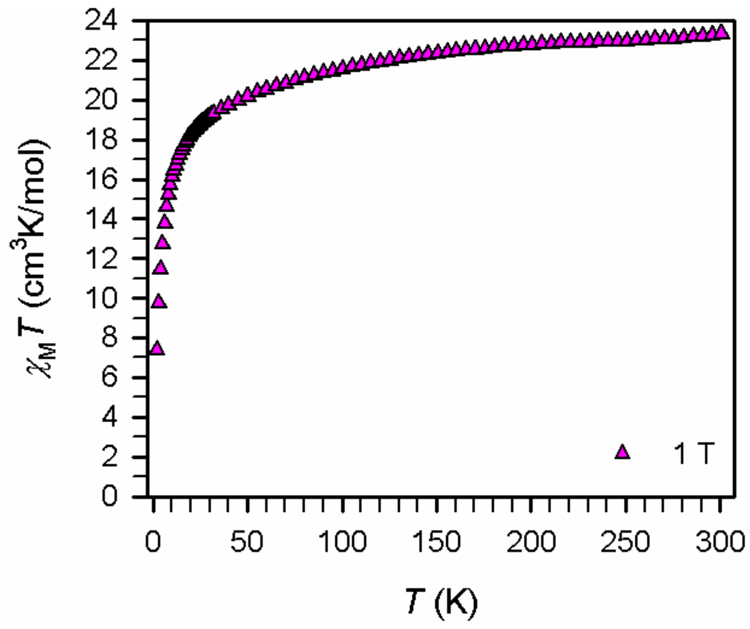

Supplementary Figure 76. Variable temperature dc susceptibility data of polycrystalline **3-Dy** collected under 1 T applied dc field.

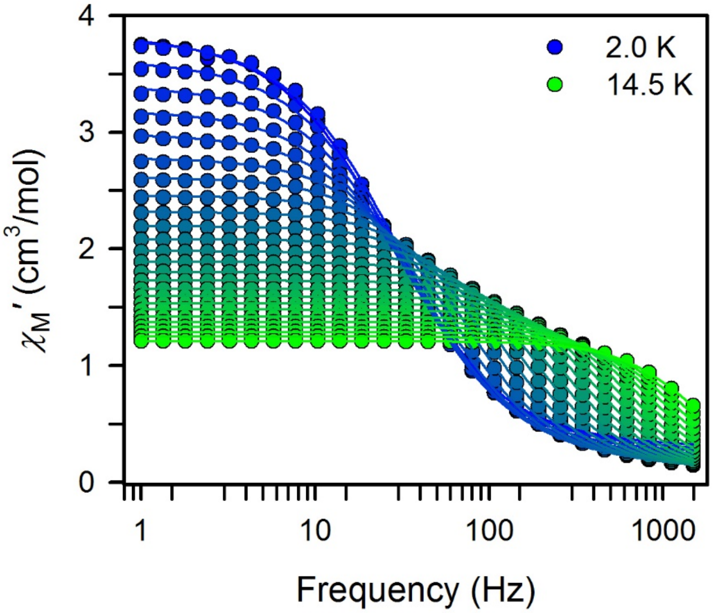

Supplementary Figure 77. In-phase (χ_M') components of the ac magnetic susceptibility for **3-Dy** under zero applied dc field from 2.0 K (blue circles) to 14.5 K (green circles). Solid lines represent a fit to the data.

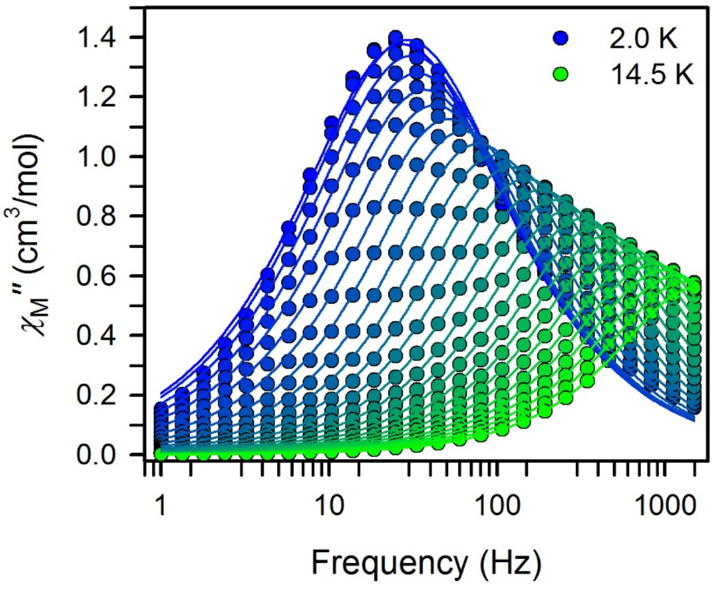

Supplementary Figure 78. Out-of-phase (χ_M'') components of the ac magnetic susceptibility for **3-Dy** under zero applied dc field from 2.0 K (blue circles) to 14.5 K (green circles). Solid lines represent a fit to the data.

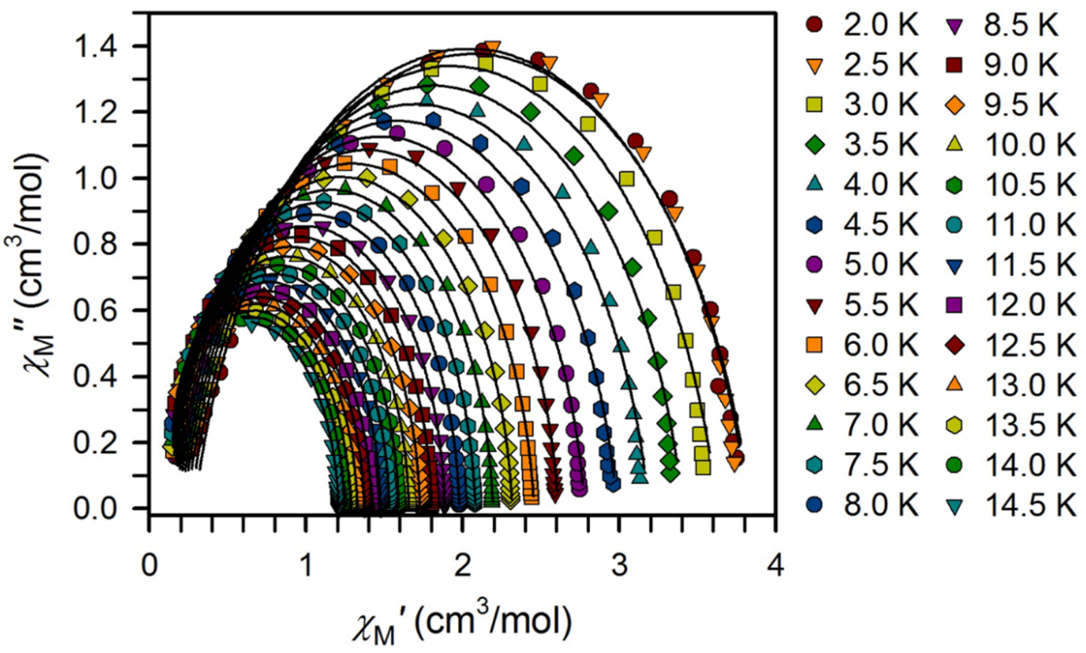

Supplementary Figure 79. Cole-Cole (Argand) plots for ac susceptibility collected from 2.0 to 14.5 K under zero applied dc field for **3-Dy**. Symbols represent the experimental data points and the points representing the fits are connected by solid black lines.

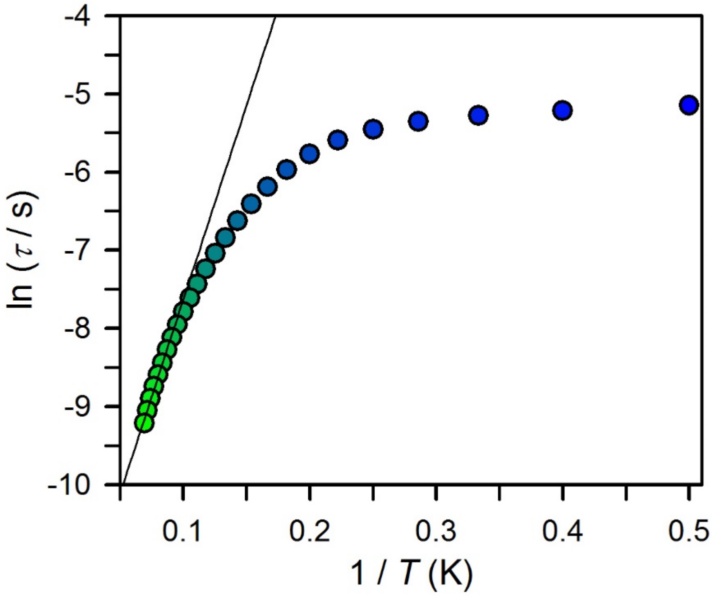

Supplementary Figure 80. Arrhenius plot of the natural log of the relaxation time, τ, versus the inverse temperature for **3-Dy** (blue to green circles). The black line corresponds to a linear fit of the data collected between 11 and 14.5 K to the Arrhenius equation yielding U_{eff} = 35(1) cm^{-1} and τ_0 = 3.4(4) × 10^{-6} s.

Supplementary Table 1. Best-fit parameters for the Arrhenius plots of **1-Tb**, **1-Dy**, **2-Tb** and **2-Dy**. H_{dc} = 0 Oe for all plots. Data in blue was extracted from ac susceptibility measurements. Data in green was extrated from ac and dc susceptibility measurements.

	τ_{QTM} (s)	C ($s^{-1}K^{-n}$)	n	τ_0 (s)	U_{eff} (cm^{-1})	$\tau_{0,2}$ (s)	$U_{eff,2}$ (cm^{-1})
**1-Tb**	-	-	-	$1.4(1) \times 10^{-9}$	242(1)	-	-
**1-Tb**	3645	-	-	$1.4(2) \times 10^{-9}$	242(2)		
**1-Dy**	0.4087	-	-	3.11×10^{-9}	110.1	-	-
**2-Tb**	-	-	-	$1.20(3) \times 10^{-7}$	277.5(1)	$1.7(2) \times 10^{-11}$	578(3)
**2-Tb**	40023	-	-	$1.3(1) \times 10^{-7}$	276(1)	$2(1) \times 10^{-11}$	564(17)
**2-Dy**	-	-	-	$2.3(1) \times 10^{-8}$	108(1)	-	-
**2-Dy**	670	2.1×10^{-6}	4.13	1.7×10^{-8}	108.1	-	-
**3-Dy**	-	-	-	$3.4(4) \times 10^{-6}$	35(1)	-	-

Supplementary Table 2. Relaxation times, τ (s), and stretch factors, b, at various temperatures, T (K) for **1-Tb**.

T (K)	τ (s)	stretch factor
4	3833.371347	0.892694887
5	3865.457747	0.889942938
8	3989.959677	0.875687774
11	3531.560387	0.8757886
12	1678.87914	0.900174082
13	429.6401364	0.851265692
14	173.0946324	0.719604464

Supplementary Table 3. Relaxation times, τ (s), and stretch factors, b, at various temperatures, T (K) for **2-Dy**.

T (K)	τ (s)	stretch factor
2	755.5613468	0.661869881
2.5	618.7185607	0.685205146
3	544.1425218	0.716568091
3.5	482.3986497	0.727883812
4	444.3636179	0.756632687
5	352.8501842	0.804868025
5.5	289.9253495	0.82651583
6	201.5475079	0.883084924
6.25	144.496536	0.867359413
6.5	109.9057266	0.851425942
6.6	93.38555175	0.878198787
6.75	76.85858841	0.795441796

Supplementary Table 4. Relaxation times, τ (s), and stretch factors, b, at various temperatures, T (K) for **2-Tb**.

T (K)	τ (s)	stretch factor
2	45431.51188	0.812878839
10	42726.8632	0.821466362
11	41287.20889	0.854499788
12	38069.61121	0.89157872
14	30489.0606	0.800277248
16	4903.991204	0.825175188
17	1461.813583	0.883622366
18	464.8666232	0.944320422
19	160.3983331	0.922300874
19.5	104.5794064	0.92194305
19.55	100.2466785	0.909506642
19.6	96.05876962	0.914170052
20	70.97870173	0.831173565
20.5	43.68173157	0.313824997

Supplementary Table 5. Comparison of predicted barriers to magnetic relaxation (U_{model}) to experimental effective barriers to magnetic relaxation (U_{eff}) for **1-Tb** and **2-Tb** using the models shown in Supplementary Figure 58, with methods of U_{model} computation described in Supplementary Methods Section 4.

**1-Tb**	**U_{model} (cm^{-1})**	**2-Tb**	**$U_{model(1)}$ (cm^{-1})**	**$U_{model(2)}$ (cm^{-1})**
g = 1.50<br>J = −22.5 cm^{-1}	270	g = 1.50<br>J = –19.4 cm^{-1}	233	466
g = 1.52<br>J = −20.2 cm^{-1}	242.4	g = 1.47<br>J = –23.1 cm^{-1}	277	554
g = 1.51<br>J = −21.45 cm^{-1}	258	g = 1.43<br>J = –27.0 cm^{-1}	324	648
**Experimental U_{eff} (cm^{-1})**	**242**	**Experimental U_{eff} (cm^{-1})**	**276**	**564**

Supplementary Table 6. Comparison of predicted barriers to magnetic relaxation (U_{model}) to experimental effective barriers to magnetic relaxation (U_{eff}) for **1-Dy** and **2-Dy** using the models shown in Supplementary Figure 59, with methods of U_{model} computation described in Supplementary Methods Section 4.

**1-Dy**	**U_{model} (cm^{-1})**	**2-Dy**	**U_{model} (cm^{-1})**
g = 1.33, J = –8.0 cm^{-1}	120	g = 1.33, J = –8.1 cm^{-1}	121.5
g = 1.39, J = –7.3 cm^{-1}	109.5	g = 1.38, J = –7.2 cm^{-1}	108
g = 1.41, J = –5.3 cm^{-1}	79.5	g = 1.40, J = –5.5 cm^{-1}	82.5
**Experimental U_{eff} (cm^{-1})**	**110**	**Experimental U_{eff} (cm^{-1})**	**108**

Supplementary Table 7. Crystallographic Data.

	[K(crypt-222)] [(Cp$^{Me4H}{}_2$Tb)$_2$N$_2^{•}$] ·(2-MeTHF)	[K(crypt-222)] [(Cp$^{Me4H}{}_2$Dy)$_2$N$_2^{•}$] ·(2-MeTHF)	[K(crypt-222)] [(Cp$^{Me4H}{}_2$Gd(2-MeTHF))$_2$N$_2^{•}$]	[K(crypt-222)(THF)] [(Cp$^{Me4H}{}_2$Tb(THF))$_2$N$_2^{•}$]	[K(crypt-222)(THF)] [(Cp$^{Me4H}{}_2$Dy(THF))$_2$N$_2^{•}$]	[K(crypt-222)(THF)] [(Cp$^{Me4H}{}_2$Gd(THF))$_2$N$_2^{•}$]
Formula	$C_{59}H_{98}KN_4O_7Tb_2$	$C_{59}H_{98}KN_4O_7Dy_2$	$C_{64}H_{108}Gd_2KN_4O_8$	$C_{66}H_{111}KN_4O_9Tb_2$	$C_{66}H_{111}KN_4O_9Dy_2$	$C_{66}H_{111}KN_4O_9Gd_2$
Temperature (K)	100(2)	100(2)	100(2)	100(2)	100(2)	100(2)
Crystal System	Monoclinic	Monoclinic	Monoclinic	Triclinic	Triclinic	Triclinic
Space Group	$P2_1/c$	$P2_1/c$	$C2/c$	$P\bar{1}$	$P\bar{1}$	$P\bar{1}$
a, b, c (Å)	13.4188(5), 15.8547(5), 28.8599(11)	13.4230(7), 15.8540(9), 28.9398(15)	27.530(4), 16.947(3), 17.447(3)	10.7750(16), 17.158(3), 18.330(2)	10.7809(5), 17.1416(7), 18.2207(8)	10.7309(4), 17.2120(5), 18.3517(6)
α, β, γ (°)	90, 97.020(2), 90	90, 96.929(3), 90	90, 126.176(6), 90	94.287(4), 92.650(3), 92.042(3)	94.401(3), 92.790(2), 92.416(3)	94.340(2), 92.563(3), 92.032(2)
V, (Å^3)	6093.9(4)	6113.7(6)	6570.3(17)	3373.1(8)	3349.8(3)	3373.90(19)
Z	4	4	4	2	2	2
Radiation, λ (Å)	MoKα, 0.71073	Synchrotron, 0.7749	MoKα, 0.71073	Synchrotron, 0.7749	Synchrotron, 0.6888	Synchrotron, 0.7749
2Θ Range for Data Collection (°)	2.844 to 50.818	4.172 to 55.808	3.022 to 50.700	4.688 to 59.802	4.246 to 64.162	4.704 to 55.952
Completeness to 2Θ	99.5% (2Θ = 50.818°)	98.1% (2Θ = 55.412°)	100% (2Θ = 50.700°)	99.9% (2Θ = 55.412°)	99.8% (2Θ = 48.822°)	99.6% (2Θ = 55.412°)
Data / Restraints / Parameters	12296 / 142 / 703	14523 / 93 / 723	6031 / 0 / 366	14933 / 0 / 765	25599 / 6 / 774	12400 / 6 / 765
Goodness of Fit on F^2	1.024	1.094	1.134	1.031	0.996	1.021
R1[a], wR2[b] (I>2σ(I))	0.0553, 0.1258	0.0716, 0.1697	0.0286, 0.0637	0.0467, 0.0853	0.0406, 0.0654	0.0653, 0.1080
R1[a], wR2[b] (all data)	0.0969, 0.1490	0.0887, 0.1835	0.0315, 0.0663	0.0880, 0.0959	0.0766, 0.0738	0.1370, 0.1287
Largest Diff. Peak and Hole (e Å^{-3})	2.830 and –1.624	3.912 and –3.706	1.471 and –0.744	3.405 and –1.484	2.615 and –1.056	2.130 and –1.724

[a]$R_1 = \sum||F_o| - |F_c||/\sum|F_o|$. [b]$wR_2 = \{\sum[w(F_o^2 - F_c^2)^2]/\sum[w(F_o^2)^2]\}^{1/2}$.

Supplementary Table 8. Crystallographic Data.

	$[(Cp^{Me4H}{}_2Tb(THF))_2N_2]\cdot(C_7H_8)$	$[(Cp^{Me4H}{}_2Dy(THF))_2N_2]\cdot(C_7H_8)$ (ref 17)	$[(Cp^{Me4H}{}_2Gd(THF))_2N_2]\cdot(C_7H_8)$ (ref 17)	$Cp^{Me4H}{}_2Tb(BPh_4B)$	$Cp^{Me4H}{}_2Dy(BPh_4)$	$Cp^{Me4H}{}_2Gd(BPh_4)$
Formula	$C_{58}H_{84}N_2O_2Tb_2$	$C_{58}H_{84}N_2O_2Dy_2$	$C_{58}H_{84}N_2O_2Gd_2$	$C_{45.50}H_{50}BTb$	$C_{45.50}H_{50}BDy$	$C_{45.50}H_{50}BGd$
Temperature (K)	100(2)	100(2)	100(2)	100(2)	100(2)	100(2)
Crystal System	Monoclinic	Monoclinic	Monoclinic	Monoclinic	Monoclinic	Monoclinic
Space Group	C2/c	C2/c	C2/c	$P2_1/n$	$P2_1/n$	$P2_1/n$
a, b, c (Å)	15.2449(9), 13.9483(8), 25.6611(15)	15.2405(7), 13.9325(6), 25.6059(11)	15.2571(8), 13.9763(7), 25.6669(14)	14.5995(9), 14.4397(9), 17.6257(11)	14.6032(8), 14.4193(8), 17.5930(10)	14.6048(9), 14.4569(9), 17.6714(10)
α, β, γ (°)	90, 104.616(3), 90	90, 105.014(2), 90	90, 104.729(2), 90	90, 102.337(3), 90	90, 102.376(2), 90	90, 102.392(2), 90
V, (Å^3)	5280.0(5)	5251.5(4)	5293.3(5)	3629.9(4)	3618.4(4)	3644.2(4)
Z	4	4	4	4	4	4
Radiation, λ (Å)	Synchrotron, 0.7749	MoKα, 0.71073	Synchrotron, 0.7749	Synchrotron, 0.7749	MoKα, 0.71073	MoKα, 0.71073
2Θ Range for Data Collection (°)	4.382 to 55.758	3.294 to 61.044	4.376 to 70.012	4.376 to 85.556	3.296 to 50.698	3.290 to 50.758
Completeness to 2Θ	99.5% (2Θ = 55.412°)	99.5% (2Θ = 52.000°)	99.4% (2Θ = 55.412°)	100% (2Θ = 55.412°)	100% (2Θ = 50.698°)	99.7% (2Θ = 50.758°)
Data / Restraints / Parameters	4828 / 369 / 374	8004 / 90 / 374	8919 / 90 / 369	20283 / 0 / 469	6631 / 45 / 469	6677 / 45 / 469
Goodness of Fit on F^2	1.132	1.080	1.047	1.013	1.080	1.089
$R1^a$, $wR2^b$ (I>2σ(I))	0.0655, 0.1547	0.0241, 0.0545	0.0301, 0.0720	0.0356, 0.0642	0.0175, 0.0432	0.0176, 0.0429
$R1^a$, $wR2^b$ (all data)	0.0731, 0.1589	0.0278, 0.0564	0.0372, 0.0761	0.0621, 0.0710	0.0183, 0.437	0.0183, 0.0434
Largest Diff. Peak and Hole(e Å^{-3})	4.762 and –2.179	1.382 and –1.117	3.188 and –1.131	0.954 and –0.849	1.039 and –0.381	0.869 and –0.346

$^a R_1 = \sum||F_o| - |F_c||/\sum|F_o|$. $^b wR_2 = \{\sum[w(F_o^2 - F_c^2)^2]/\sum[w(F_o^2)^2]\}^{1/2}$.

Supplementary Table 9. Crystallographic Data.

	$Cp^{Me4H}{}_2$TbAllyl	$Cp^{Me4H}{}_2$DyAllyl	$Cp^{Me4H}{}_2$GdAllyl
Formula	$C_{21}H_{31}Tb$	$C_{21}H_{31}Dy$	$C_{21}H_{31}Gd$
Temperature (K)	100(2)	100(2)	100(2)
Crystal System	Triclinic	Monoclinic	Triclinic
Space Group	$P\bar{1}$	$P2_1/n$	$P\bar{1}$
a, b, c (Å)	8.8121(7), 15.0961(12), 15.1222(12)	8.7680(3), 15.0735(5), 14.3016(5)	8.7988(3), 15.1568(6), 15.2231(5)
α, β, γ (°)	95.663(4), 101.885(4), 102.973(4)	90, 91.1436(15), 90	95.8609(19), 101.7150(18), 102.6738(19)
V, (Å^3)	1896.0(3)	1889.79(11)	1916.46(12)
Z	4	4	4
Radiation, λ (Å)	MoKα, 0.71073	MoKα, 0.71073	Synchrotron, 0.6888
2Θ Range for Data Collection (°)	2.784 to 61.222	3.926 to 61.072	4.086 to 79.414
Completeness to 2Θ	99.7% (2Θ = 52.000°)	100% (2Θ = 52.000°)	99.8% (2Θ = 48.822°)
Data / Restraints / Parameters	11584 / 0 / 413	5777 / 0 / 207	25079 / 0 / 413
Goodness of Fit on F^2	1.091	1.148	1.010
R1[a], wR2[b] (I>2σ(I))	0.0229, 0.0564	0.0193, 0.0461	0.0385, 0.0621
R1[a], wR2[b] (all data)	0.0251, 0.0579	0.0209, 0.0469	0.0717, 0.0696
Largest Diff. Peak and Hole (e Å^{-3})	1.661 and –0.954	1.920 and –1.106	1.041 and –0.985

[a]$R_1 = \sum||F_o| - |F_c||/\sum|F_o|$. [b]$wR_2 = \{\sum[w(F_o^2 - F_c^2)^2]/\sum[w(F_o^2)^2]\}^{1/2}$.

Supplementary References

1. http://www.jcmeyer-solventsystems.com.
2. W. J. Evans, D. S. Lee, M. A. Johnston, J. W. Ziller, The Elusive $(C_5Me_4H)_3Lu$: Its Synthesis and $LnZ_3/K/N_2$ Reactivity. *Organometallics* **24**, 6393-6397 (2005).
3. B. J. Barker, P. G. Sears, Conductance behavior of some ammonium and partially substituted ammonium tetraphenylborates in 3-methyl-2-oxazolidone and 3-tert-butyl-2-oxazolidone at 25.deg. *The Journal of Physical Chemistry* **78**, 2687-2688.
4. Demir, S., Lorenz, S. E., Fang, M., Furche, F., Meyer, G., Ziller, J. W. & Evans, W. J. *J. Am. Chem. Soc.*, **132**, 11151–11158 (2010).
5. (a) Orbach, R. *Proc. R. Soc. London, Ser. A*, **264**, 458, (1961); (b) Orbach, R. *Proc. R. Soc. London, Ser. A,* **264**, 485, (1961); (c) Walker, M. B. *Can. J. Phys.*, **46**, 1347, (1968).
6. Eaton, G. R.; Eaton, S. S. In *Distance Measurements in Biological Systems by EPR*, Berliner, L. J., Ed.; Biological Magnetic Resonance, Vol. 19; Kluwer Academic/Plenum Publishers: New York, 2000.
7. Chibotaru, L. F.; Iwahara, N. Ising Exchange Interaction in Lanthanides and Actinides. *New J. Phys.* **17**, 103028 (2015).
8. K. L. Trojan, Strong Exchange Coupling in Lanthanide Bis(Phthalocyaninato) Sandwich Compounds. *J. Appl. Phys.* **69**, 6007–6009 (1991).
9. N.F. Chilton, *et. al.,* PHI: a Powerful New Program for the Analysis of Anisotropic Monomeric and Exchange-Coupled Polynuclear D- and F-Block Complexes. *J. Comput. Chem.* **34**, 1164–1175 (2013).
10. Sheldrick, G. M. *CELL NOW V2008/2*. (Bruker AXS Inc, 2008).
11. Bruker Analytical X-ray Systems, Inc. *SAINT and APEX 2 Software for CCD Diffractometers*. (Bruker Analytical X-ray Systems, Inc., 2000).
12. Sheldrick, G. M. *TWINABS, Version 2012/1*. (University of Göttingen, 2012).
13. Sheldrick, G. M. *SADABS*. (Bruker Analytical X-ray Systems, Inc., 2014).
14. Sheldrick, G. M. SHELXT - integrated space-group and crystal-structure determination. *Acta Crystallogr A Found Adv* **71,** 8 (2015).
15. Sheldrick, G. M. *SHELXL*. (University of Göttingen, Germany, 2014).
16. Dolomanov, O. V., Bourhis, L. J., Gildea, R. J., Howard, J. A. K. & Puschmann, H. OLEX2: a complete structure solution, refinement and analysis program. *Journal of Applied Crystallography* **42,** 339–341 (2009).
17. The crystal structures of $(Cp^{Me4H}{}_2Gd(THF))_2(\mu\text{-}N_2)$ and $(Cp^{Me4H}{}_2Dy(THF))_2(\mu\text{-}N_2)$ were published in: Megan E. Fieser, David H. Woen, Jordan F. Corbey, Thomas J. Mueller, Joseph W. Ziller, William J. Evans Raman spectroscopy of the N-N bond in rare earth dinitrogen complexes, *Dalton Trans.* **45**, 14634-14644 (2016). However, these compounds were synthesized by reduction of $(C_5Me_4H)_3Ln(THF)$. In this study, no magnetic data for these compounds were reported. $(Cp^{Me4H}{}_2Dy(THF))_2(\mu\text{-}N_2)$ was reported in a different cell.
